# The Effectiveness of Combining Nonmobile Interventions With the Use of Smartphone Apps With Various Features for Weight Loss: Systematic Review and Meta-analysis

**DOI:** 10.2196/35479

**Published:** 2022-04-08

**Authors:** Jumana Antoun, Hala Itani, Natally Alarab, Amir Elsehmawy

**Affiliations:** 1 American University of Beirut Beirut Lebanon

**Keywords:** obesity, weight loss, mobile app, self-monitoring, behavioral, tracker, behavioral coaching, coach, dietitian, mobile phone

## Abstract

**Background:**

The effectiveness of smartphone apps for weight loss is limited by the diversity of interventions that accompany such apps. This research extends the scope of previous systematic reviews by including 2 subgroup analyses based on nonmobile interventions that accompanied smartphone use and human-based versus passive behavioral interventions.

**Objective:**

The primary objective of this study is to systematically review and perform a meta-analysis of studies that evaluated the effectiveness of smartphone apps on weight loss in the context of other interventions combined with app use. The secondary objective is to measure the impact of different mobile app features on weight loss and mobile app adherence.

**Methods:**

We conducted a systematic review and meta-analysis of relevant studies after an extensive search of the PubMed, MEDLINE, and EBSCO databases from inception to January 31, 2022. Gray literature, such as abstracts and conference proceedings, was included. Working independently, 2 investigators extracted the data from the articles, resolving disagreements by consensus. All randomized controlled trials that used smartphone apps in at least 1 arm for weight loss were included. The weight loss outcome was the change in weight from baseline to the 3- and 6-month periods for each arm. Net change estimates were pooled across the studies using random-effects models to compare the intervention group with the control group. The risk of bias was assessed independently by 2 authors using the Cochrane Collaboration tool for assessing the risk of bias in randomized trials.

**Results:**

Overall, 34 studies were included that evaluated the use of a smartphone app in at least 1 arm. Compared with controls, the use of a smartphone app–based intervention showed a significant weight loss of –1.99 kg (95% CI –2.19 to –1.79 kg; *I*^2^=81%) at 3 months and –2.80 kg (95% CI –3.03 to –2.56 kg; *I*^2^=91%) at 6 months. In the subgroup analysis, based on the various intervention components that were added to the mobile app, the combination of the mobile app, tracker, and behavioral interventions showed a statistically significant weight loss of –2.09 kg (95% CI –2.32 to –1.86 kg; *I*^2^=91%) and –3.77 kg (95% CI –4.05 to –3.49 kg; *I*^2^=90%) at 3 and 6 months, respectively. When a behavioral intervention was present, only the combination of the mobile app with intensive behavior coaching or feedback by a human coach showed a statistically significant weight loss of –2.03 kg (95% CI –2.80 to –1.26 kg; *I*^2^=83%) and –2.63 kg (95% CI –2.97 to –2.29 kg; *I*^2^=91%) at 3 and 6 months, respectively. Neither the type nor the number of mobile app features was associated with weight loss.

**Conclusions:**

Smartphone apps have a role in weight loss management. Nevertheless, the human-based behavioral component remained key to higher weight loss results.

## Introduction

### Background

Obesity has become a major, rising health epidemic worldwide. As a complex multifactorial disease, the management of obesity is challenging because there is no single effective treatment. Of late, there has been great interest in using apps for weight loss. Mobile apps were effective for weight loss [1-6] by using different behavior change techniques to a certain extent [1-6]. These behavior change techniques include intention formation, goal setting, barrier identification, problem solving, planning, general encouragement, self-monitoring of behavior, feedback on performance, social support, and social comparison [4,7].

On the basis of recent research, mobile apps help users to adhere to self-monitoring and weight loss goals better than the traditional pen-and-paper methods and other mobile health interventions (web-based or PDA) [8-12]. In 2015, Mateo et al [13] conducted the first meta-analysis that focused on mobile apps and found a modest weight loss of –1.04 kg (95% CI –1.75 to –0.34 kg; *I*^2^=41%) among mobile app users. In 2014 and 2015, similar results were found by Khokhar et al [14], Hutchesson et al [15], and Liu et al [16] after they expanded the inclusion criteria to include email, SMS text messaging, monitoring devices, and smartphones. Cai et al [17] observed similar findings when they measured the effect of mobile apps in patients with diabetes mellitus. In 2020, Islam et al [18] updated the literature review and extended the scope of the previous meta-analysis performed by Mateo et al [13] by including more subgroup analyses.

Most of the interventions are smartphone apps combined with other behavioral nonmobile interventions; yet, it’s unclear whether the app’s effect on weight loss is due solely to its use or to the addition of the behavioral component. If the behavioral component relies on human coaches and personalized feedback by dietitians, this will affect the scalability of the mobile app used for weight loss. Personalized feedback provided by mobile apps has proven to be an essential feature of such apps because the feedback increases users’ logging-in frequency and engagement with the apps [19-23]. Personalized feedback from an interventionist or professional also affected the results positively [24-26]. Thus, the combination of mobile app use with in-person contacts such as coaching or counseling sessions, interventionist feedback, web-based chatting, or telephone calls with professionals was more effective than mobile app use alone [19,24,27-29].

### Objectives

The aim of this meta-analysis is to evaluate the effectiveness of mobile app interventions alone or in combination with other behavioral interventions on weight loss. Although Lyzwinski [30] analyzed the intervention components of mobile devices in a narrative review, the author did not compare the effect of the various components on weight loss. This research extends the scope of the previous systematic reviews by including 2 subgroup analyses based on nonmobile interventions that accompanied smartphone use and human-based versus passive behavioral interventions. The results are organized according to duration because it is inaccurate to compare weight loss at 3 months with weight loss at 6 or 12 months. The secondary outcomes of the study include the impact of different mobile app features on weight loss and mobile app adherence.

## Methods

This systematic review of the literature and quantitative meta-analysis was conducted following the PRISMA (Preferred Reporting Items for Systematic Reviews and Meta-Analyses) guidelines [31] to measure the effectiveness of mobile app interventions alone or in combination with other behavioral interventions on weight loss. The secondary outcomes of the study include the impact of different mobile app features on weight loss and adherence.

### Protocol and Registration

The protocol was not registered.

### Search Strategy

The PubMed, MEDLINE, and EBSCO databases were searched for relevant studies published between the database inception date and January 31, 2022. The search strategy incorporated keywords. The terms used included *weight loss*, *obesity*, *overweight*, *smartphone*, *mobile phone*, *cell phone*, *mHealth*, *eHealth*, and *adherence*. The search was then filtered to studies involving randomized controlled trials. All previous systematic reviews and meta-analyses were researched to find further missing studies. EndNote X9 (Clarivate) was used to remove duplicate publications and for screening purposes (for further information on the search strategy, please see the example provided in Multimedia Appendix 1). Multimedia Appendix 2 includes a summary of the reasons for excluding articles.

### Study Selection

To investigate the effectiveness of nonapp interventions combined with smartphone apps, studies were eligible if (1) the design included randomized controlled trials, (2) they included the use of a smartphone app in at least 1 arm, (3) weight loss was an outcome, and (4) the population consisted of adults. There was no restriction on the population regarding overweight versus obesity or being diagnosed with chronic diseases, the language or year of publication, length of interventions, or follow-up duration. Gray literature, such as abstracts and conference proceedings, was included. We also searched the lists of references of the articles that we included.

On the basis of the eligibility criteria, 2 research team members (JA and HI) independently screened all the articles by study title and abstract. If the information listed in the title or abstract was insufficient to determine the study’s relevance, the full text of the study was selected to be reassessed later. Next, each member further screened the selected studies at the full-text level. Any disagreements were resolved by consensus.

### Data Collection Process and Data Items

Working independently, 2 investigators (JA and HI) extracted the data from the articles, resolving disagreements by consensus. A form developed by using the KoBo toolbox (Kobo Inc) was used to extract data from eligible research papers, including digital object identifier, the title of the study, year of publication, type of article, country of study, population, sample size, trial name, trial period, number of arms, and details of each arm. Mean body weight changes were recorded from baseline to the end of the trial with SDs and adherence-related outcomes. SD or 95% CI was recorded if available. Neither authorship nor publication journal nor study results were blinded for data extraction.

### Risk of Bias in Individual Studies

The risk of bias was assessed independently by 2 authors (NA and AE) using the Cochrane Collaboration tool for assessing the risk of bias in randomized trials [31]. The tool covers the following bias domains: selection bias, performance bias, detection bias, attrition bias, and reporting bias. Each author independently judged each domain as having a low, unclear, or high risk of bias. When differences of opinion arose between the 2 evaluators, the item was discussed until a consensus was reached. JA randomly selected a few articles to assess the risk of bias and compared the results with those of the 2 primary evaluators to consolidate the assessments further.

### Measured Outcomes

The study’s primary outcome is the mean weight change (measured in kilograms) from baseline to 3, 6, and 12 months. All outcomes were recorded if the study measured the outcome at multiple points. Adherence measures were examined, and a systematic review of the literature was performed.

### Data Analysis and Synthesis of Results

The meta-analysis evaluated whether smartphone app interventions were effective on their own or whether other behavior interventions were necessary for weight loss. A fixed effect model was used to obtain the overall effect size across included studies and its associated 95% CI. On the basis of the studies chosen, the outcome of weight loss was measured as the weight change from baseline to the 3-, 6-, or 12-month periods. When SD was not mentioned, the variance was calculated from the 95% CI. We examined heterogeneity using the *I*^2^ test, which describes the percentage of variability in effect size estimates because of heterogeneity rather than sampling error. The statistical analyses were performed using Review Manager software (version 5.4; Cochrane Training).

In studies with more than one arm that included an app, inverse variance meta-analysis was used to produce an overall effect size across all treatment arms, creating a single intervention-versus-control comparison for each study. The exact process was performed when there was more than one control arm.

All the interventions used were reviewed by 2 authors (JA and HI), who then grouped them into the following categories: smartphone apps, trackers (weighing scale, step tracker, or bite counter), behavioral therapy or advice (podcasts, telephone calls, booklets, SMS text messages, and in-person meetings), feedback (SMS text messages, email, oral, or written by a coach), self-monitoring, social support (social media or web-based forum), meal replacement, and financial incentives. Human-based active behavioral coaching or feedback included in-person meetings, interaction with the interventionist through Twitter or chat feature of the app, and tailored feedback from a coach or interventionist using telephone calls, SMS text messages, or group sessions. The passive behavioral component included passive standardized behavioral messages as part of the app, Facebook, or podcasts. The apps were also classified according to the following features: self-monitoring, education, feedback, social support, rewards, and gamification. On the basis of these features, 2 associations were later analyzed. Using an independent 2-tailed *t* test, the first analysis examined the association between weight loss and each app feature. The second analysis, using 1-way analysis of variance, studied the association between weight loss and the number of features. We aim to measure the association between adherence percentage and app features. Nevertheless, the definition of adherence was not homogeneous, and we ultimately conducted a systematic review of the adherence outcome.

Concerning the intervention period, it included both active treatment and follow-up. If provided, the baseline weight of the intervention group was noted; however, if it was not available, the average weight of participants in the whole cohort was captured from the demographics table.

## Results

### Study Selection and Characteristics

**Figure 1:**
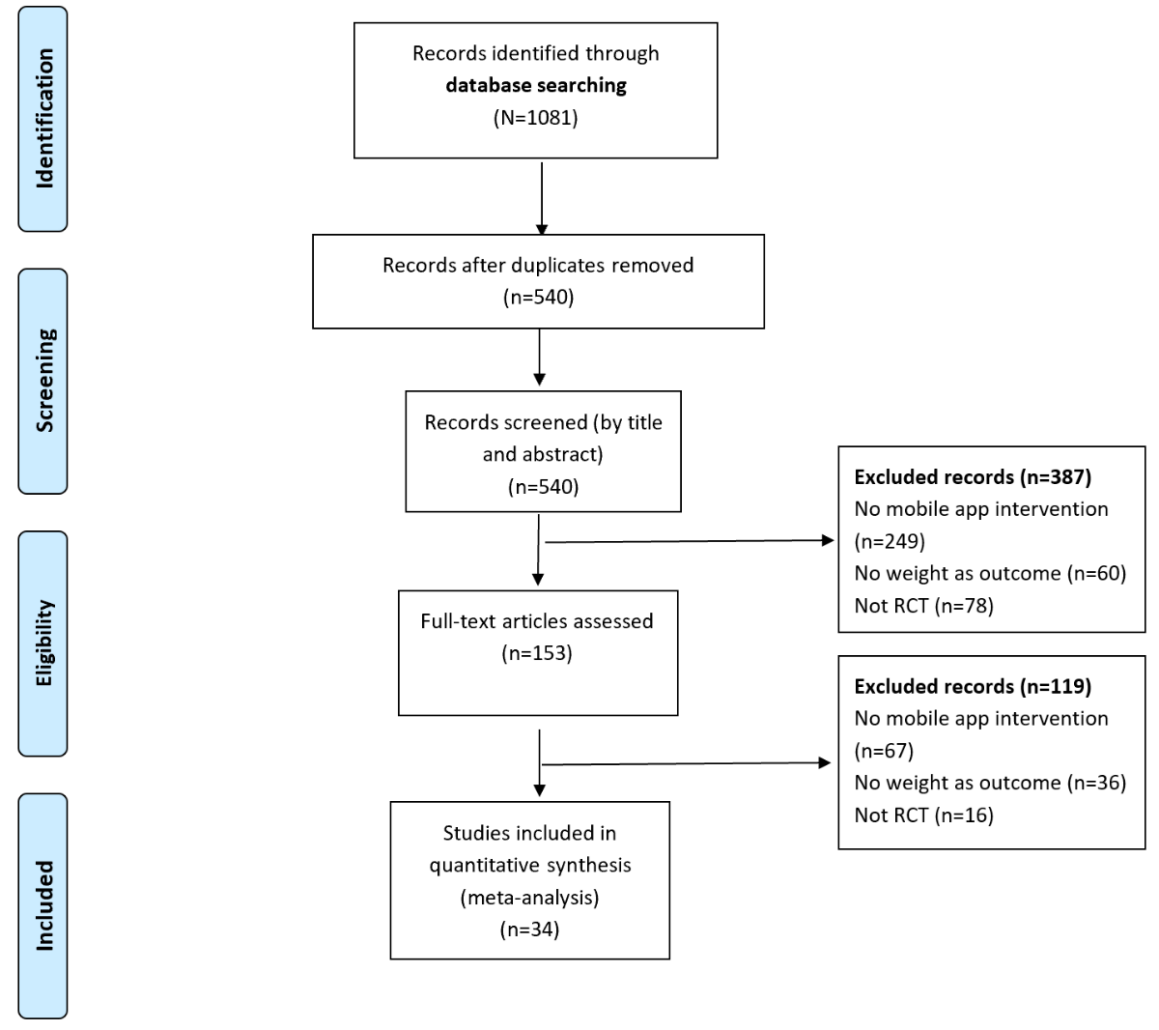
Flowchart of selection of the articles based on PRISMA (Preferred Reporting Items for Systematic Reviews and Meta-Analyses) guidelines. RCT: randomized controlled trial.

The search strategy enabled us to compile 1081 articles from different resources, of which 34 (3.14%) were included in this meta-analysis (Figure 1). The studies selected were published between 2011 and January 31, 2022; however, 68% (23/34) of the articles were published in the last 5 years (2017-2021). The studies were conducted in the United States (22/34, 65%), Australia and New Zealand (3/34, 9%), Europe (4/34, 12%; Germany: 1/4, 25%; Spain: 1/4, 25%; and the United Kingdom: 2/4, 50%), and Asia (5/34, 15%; Japan: 1/5, 20%; Singapore 1/5, 20%; and South Korea: 3/5, 60%). The sample size ranged from 16 to 440, with a mean of 113.09 (SD 94.1). The population in the studies ranged from men and women from the general population to adults at risk for diabetes as well as adults with diseases such as cardiology issues, diabetes, or metabolic syndrome. Of the 34 studies, 7 (20%) used a theoretical framework: social cognitive theory (3/7, 43%), social cognitive theory and transtheoretical model (2/7, 29%), and social cognitive theory and self-efficacy theory (2/7, 29%). Tables 1 and 2 present a summary of the characteristics of the included studies.

**Table 1:** Description of the population, sample size, and baseline demographics of the included studies (N=34).

Study	Country	Population of study	Sample size, n	Trial period (weeks)	Age (years), mean (SD) or mean (95% CI)	Baseline BMI (kg/m^2^), mean (SD) or mean (95% CI)	Baseline weight (kg). mean (SD) or mean (95% CI)
Bender and Cooper, 2017 [32]	United States	Adults with diabetes	45	24	57.6 (9.8)	30.1 (4.6)	75.8 (15.4)
Fukuoka et al, 2015 [33]	United States	Adults with prediabetes	61	20	55.2 (9.0)	33.3 (6.0)	86.2 (18.5)
Whitelock et al, 2019 [34]	United Kingdom	Adults	107	8	42.8 (10.5)	35.9 (6.8)	100.5 (20.4)
Vaz et al, 2018 [35]	United States	Adults	28	24	39.5 (3.71)	34.5 (1.3)	94.3 (3.42)
Thompson-Felty and Johnston, 2017 [26]	United States	Adults	30	8	—^a^	—	—
Rogers et al, 2016 [36]	United States	Adults	39	24	39.9 (11.5)	39.5 (2.8)	111.5 (11.5)
Svetkey et al, 2015 [29]	United States	Adults	365	96	29.4 (4.3)	35.2 (7.8)	101 (23.7)
Thomas et al, 2019 [37]	United States	Adults	276	72	55.1 (9.9)	35.2 (5)	95.9 (17.0)
Brindal et al, 2013 [38]	Australia	Adult women	58	8	42 (—)	34 (—)	92.4 (14.7)
Laing et al, 2014 [39]	United States	Adult primary care patients	212	24	43.3 (14.3)	33.4 (7.09)	—
Spring et al, 2017 [28]	United States	Adults	96	48	39.3 (11.7)	34.6 (3.0)	94.8 (12.4)
Shin et al, 2017 [40]	South Korea	Adult men	105	12	27.8 (—)	29.8 (—)	91.4 (10.6)
Ross and Wing, 2016 [27]	United States	Adults	80	24	51.1 (11.7)	33.0 (3.4)	—
Gilmore et al, 2017 [41]	United States	Postpartum women	40	16	26.0 (5.2)	31.3 (3.2)	83.8 (13.5)
Tanaka et al, 2018 [42]	Japan	Adults	112	12	45.6 (10.2)	28.0 (3.3)	83.1 (11.1)
Allen et al, 2013 [43]	United States	Adults	68	24	44.9 (11.1)	34.3 (3.9)	97.3 (16.2)
Stephens et al, 2017 [44]	United States	Adults	62	12	20.0 (18.0-25.0)	28.5 (25.0-40.4)	82.8 (61-117.5)
Hales et al, 2016 [45]	United States	Adults	51	12	46.2 (12.4)	34.7 (6.0)	102.1 (91.9-112.2)
Hartman et al, 2016 [46]	United States	Adult women with elevated risk for breast cancer	54	24	59.5 (5.6)	31.9 (3.5)	86.3 (10.2)
Haufe et al, 2019 [47]	Germany	Adults with metabolic syndrome	314	24	48.1 (8.1)	33.3 (5.4)	106.7 (19.1)
Turner-McGrievy et al, 2017 [48]	United States	Adults who were overweight	81	24	48.6 (11.7)	33.4 (4.8)	—
Jospe et al, 2017 [49]	New Zealand	Adults with overweight or obesity	250	48	44.4 (10.2)	33.5 (4.5)	99.1 (17.3)
Burke et al, 2017 [50]	United States	Adults	39	12	44.85 (12.75)	33.76 (4.28)	93.15 (15.89)
Lee et al, 2019 [51]	South Korea	Adults with metabolic syndrome	129	24	30.59 (—)	—	71.6 (12.2)
Turner-McGrievy and Tate, 2011 [52]	United States	Adults	96	24	42.6 (10.7)	32.9 (4.8)	—
Monroe et al, 2019 [53]	United States	Adults	36	48	44.67 (8.96)	36.22 (7.53)	97.78 (21.04)
Choi et al, 2019 [54]	United States	Adult patients with cardiology issues	100	24	57.2 (1.8)	29.5 (0.6)	84.8
Evangelista et al, 2018 [55]	United States	Adults with heart failure	16	12	52.3 (8.5)	—	—
Kurtzman et al, 2018 [56]	United States	Adults	196	36	42.3 (11.5)	36.0 (5.2)	102.0 (18.8)
Carter et al, 2013 [12]	United Kingdom	Adults	128	24	41.2 (8.5)	33.7 (4.2)	96.4 (16)
Duncan et al, 2020 [57]	Australia	Adults	116	48	44.5 (10.4)	31.7 (3.9)	90.7 (14.3)
Lim et al, 2021 [58]	Singapore	Adults with diabetes	204	24	51.2 (9.7)	84.0 (12.6)	30.6 (4.3)
Ahn et al, 2020 [59]	South Korea	Adults	50	6	26.0 (4.8)	77.1 (11.5)	26.7 (2.7)
Lugones-Sanchez et al, 2020 [60]	Spain	Adults	440	12	47.4 (10.0)	89.7 (13.1)	32.8 (3.3)

^a^Not available.

**Table 2:** Description of the study arms, app features, and theoretical frameworks (N=34).

Study	Number of arms	Intervention description	Control description	App features	Commercial app	Theoretical framework of the app
Bender and Cooper, 2017 [32]	2	App+tracker+social support (Facebook)+behavior or advice (in-person meetings)	Tracker+waitlist	Self-monitoring	—^a^	Social cognitive theory and transtheoretical model for health behavior change
Fukuoka et al, 2015 [33]	2	App+tracker+behavior or advice (in-person meetings)	Tracker+behavior or advice (booklet)	Self-monitoring+education+feedback+gamification	—	—
Whitelock et al, 2019 [34]	2	App+behavior or advice (booklet+SMS text messages)	Behavior or advice (booklet+SMS text messages)	Self-monitoring+education+feedback+rewards	—	—
Vaz et al, 2018 [35]	2	App+tracker	Behavior or advice (in-person meetings)	Self-monitoring+education+social support+rewards	—	—
Thompson-Felty and Johnston, 2017 [26]	3	All 3 arms included an app: arm 1: app; arm 2: app+feedback; arm 3: app	No control	Arm 1track pictures of foods; Arm 2: track picture of foods+feedback; arm3: track pictures of foods+education	—	—
Rogers et al, 2016 [36]	3	App+tracker+behavior or advice (telephone call+booklet)	One arm: tracker+behavior or advice (telephone call+ booklet)+web-based self-monitoring; second arm: behavior or advice (in-person meetings+booklet)+feedback (oral by coach)+paper-based self-monitoring	Self-monitoring	—	—
Svetkey et al, 2015 [29]	3	Arm 1: app+tracker; arm 2: app+tracker+social support (social buddy)+behavior or advice (in-person meetings)	Behavior or advice (booklet)	Arm 1: self-monitoring+feedback+social support+gamification; arm 2: self-monitoring	—	Social cognitive theory and transtheoretical model
Thomas et al, 2019 [37]	3	App+behavior or advice (in-person meetings)+feedback (oral and written by coach)	Arm 1: behavior or advice (in-person meetings)+feedback (oral and written by coach)+self-monitoring (paper diaries); arm 2: behavior or advice (dietary advice)+feedback (oral or written by coach)+self-monitoring (paper diaries)	Self-monitoring+education+social support	MyFitnessPal	—
Brindal et al, 2013 [38]	2	App+meal replacement	Meal replacement	Self-monitoring+education+feedback+rewards	—	—
Laing et al, 2014 [39]	2	App	Usual care	Self-monitoring+feedback+social support	MyFitnessPal	—
Spring et al, 2017 [28]	3	App+tracker+behavior or advice (in-person meetings)+feedback (telephone call by coach)	Arm 1: behavior or advice (in-person meetings)+feedback (telephone call by coach)+self-monitoring (paper diaries); arm 2: behavior or advice (DVDs)+self-monitoring (paper diaries)	Self-monitoring+social support	—	—
Shin et al, 2017 [40]	3	Arm 1: app+tracker+behavior or advice (in-person meetings); arm 2: app+tracker+behavior or advice (in-person meeting)	Behavior or advice (in-person meetings)	Arm 1: self-monitoring+feedback+rewards; arm 2: self-monitoring	FitLife	—
Ross and Wing, 2016 [27]	3	Arm 1: app+tracker+behavior or advice (in-person meetings); arm 2: app+tracker+behavior or advice (in-person meetings and telephone calls)	Behavior or advice (in-person meetings)+tracker+self-monitoring (paper diaries)	Arms 1 and 2: self-monitoring+feedback	Fitbit	—
Gilmore et al, 2017 [41]	2	App+tracker	Usual care	Self-monitoring+education+feedback	—	—
Tanaka et al, 2018 [42]	2	App	Usual care	Self-monitoring+education+feedback+social support	FiNC	—
Allen et al, 2013 [43]	4	Arm 1: app; arm 2: app+behavior or advice (intensive in-person meetings); arm 3: app+behavior or advice (less intensive in-person meetings)	Behavior or advice (in-person meetings)	Arms 1, 2, and 3: self-monitoring+feedback+social support	Lose it!	Social cognitive theory
Stephens et al, 2017 [44]	2	App+behavior or advice (in-person meetings)+feedback (SMS text messages by health coach)	Behavior or advice (in-person session)	Self-monitoring+social support	Lose It!	Social cognitive theory and self-efficacy theory
Hales et al, 2016 [45]	2	Arm 1: app+behavior or advice (podcasts); arm 2: app+behavior or advice	No control	Arm 1: self-monitoring+feedback+social support+rewards; arm 2: self-monitoring	FatSecret	Social cognitive theory
Hartman et al, 2016 [46]	2	App+tracker+behavior or advice (telephone calls with coach)	Behavior or advice (telephone calls)	Self-monitoring	MyFitnessPal	—
Haufe et al, 2019 [47]	2	App+tracker+behavior or advice (in-person meetings)	Waitlist	Self-monitoring+education+feedback	—	—
Turner-McGrievy et al, 2017 [48]	2	App+tracker+behavior or advice (podcasts)	Tracker (bite counter)+behavior or advice (podcasts)	Self-monitoring	FatSecret	—
Jospe et al, 2017 [49]	5	App	Arm 1: behavior or advice (in-person meetings)+feedback (email); arm 2: behavior or advice (in-person meetings)+self-monitoring (hunger using capillary glucose monitor); arm 3: behavior or advice (monthly in-person meetings); arm 4: behavior or advice (in-person session at baseline)	Self-monitoring	MyFitnessPal	—
Burke et al, 2017 [50]	3	Arm 1: app; arm 2: app+(in-person meetings)+social support (Facebook); arm 3: app+social support (Facebook)	No control	Self-monitoring+feedback	Lose It!	—
Lee et al, 2019 [51]	3	Arm 1: app; arm 2: app+behavior or advice (in-person meetings)	Usual care	Self-monitoring	—	—
Turner-McGrievy and Tate, 2011 [52]	2	App+behavior or advice (podcasts)+social support (Twitter)	Podcasts	Self-monitoring	FatSecret	Social cognitive theory
Monroe et al, 2019 [53]	2	Arm 1: app+tracker+behavior or advice (in-person meetings)+social support (support partners)+feedback (written by coach through website); arm 2: app+tracker+behavior or advice (in-person meetings)+feedback (written by coach through website)	No control	Self-monitoring+social support	MyFitnessPal	—
Choi et al, 2019 [54]	2	App+behavior or advice booklet (1-hour in-person meeting)	Behavior or advice (in-person meetings+telephone calls+booklets)	Self-monitoring+education+feedback+gamification	—	—
Evangelista et al, 2018 [55]	2	Arm 1: app+tracker+behavior or advice (in-person meetings)+feedback (SMS text messages by coach); arm 2: app+tracker+behavior or advice (in-person meetings)	No control	Self-monitoring	GetFit	—
Kurtzman et al, 2018 [56]	3	Arm 1: app+tracker+social support (support partners)+feedback (SMS text messages or email or both by primary care practitioner)+financial incentives; arm 2: app+tracker+social support (support partners)+financial incentives; arm 3: app+tracker+social support	No control	Self-monitoring	Withings Health Mate	—
Carter et al, 2013 [12]	3	App+social support (web-based forum)	Arm 1: social support (web-based forum)+self-monitoring (web-based); arm 2: social support (social forum)+self-monitoring (paper diaries)	Self-monitoring+feedback	My Meal Mate study mobile app	—
Duncan et al, 2020 [57]	3	Arm 1: app+tracker+advice or behavior (in-person counseling sessions); arm 2: same as arm 1+sleep goals	Waitlist	Self-monitoring+education+feedback	—	Social cognitive theory and self-efficacy theory
Lim et al, 2021 [58]	2	App+behavior or advice (dietitian contact through the app)	Not explained	Self-monitoring+education+feedback	—	—
Ahn et al, 2020 [59]	2	App	Paper diary	Self-monitoring	—	—
Lugones-Sanchez et al, 2020 [60]	2	App+tracker+behavior or advice (5-minute baseline session)	Standard counseling session (5-minute baseline session)	Self-monitoring	—	—

^a^Not available.

### Assessment of the Risk

**Figure 2:**
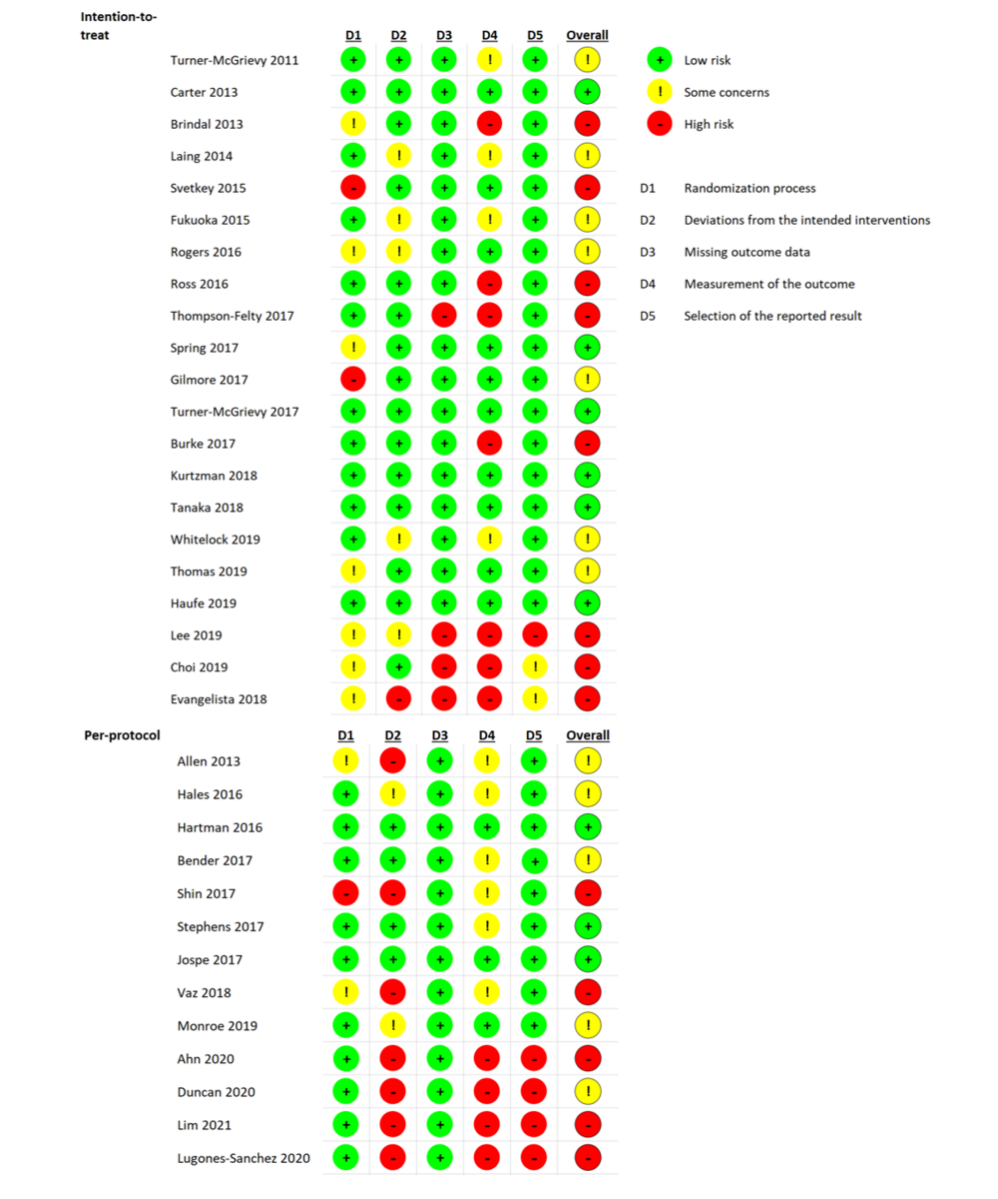
Summary of the risk of bias assessment of the included studies performed by using the Cochrane Collaboration tool [12,26-29,32-60].

The studies selected for the meta-analysis used the following two types of analyses for the intervention results: intention-to-treat and per-protocol analyses. The intention-to-treat studies (n=21) were assessed for risk, and the per-protocol studies (n=13) were similarly evaluated. Among the 21 intention-to-treat studies, 28% (6/21) were determined to be low risk, 33% (7/21) had some concerns, and 38% (8/21) were determined to be high risk. The highest risk was related to outcome measurement because of the nature of the app and the consequent lack of blinding. Similarly, among the 13 per-protocol studies, 23% (3/13) were determined to be low risk, 38% (5/13) had some concerns, and 38% (5/13) were determined to be high risk. In the per-protocol studies, high risk was due to deviation from the intended interventions. Figure 2 [12,26-29,32-60] shows a summary of the risk of bias assessment of the included studies.

### Smartphone App Intervention and Weight Loss

**Figure 3:**
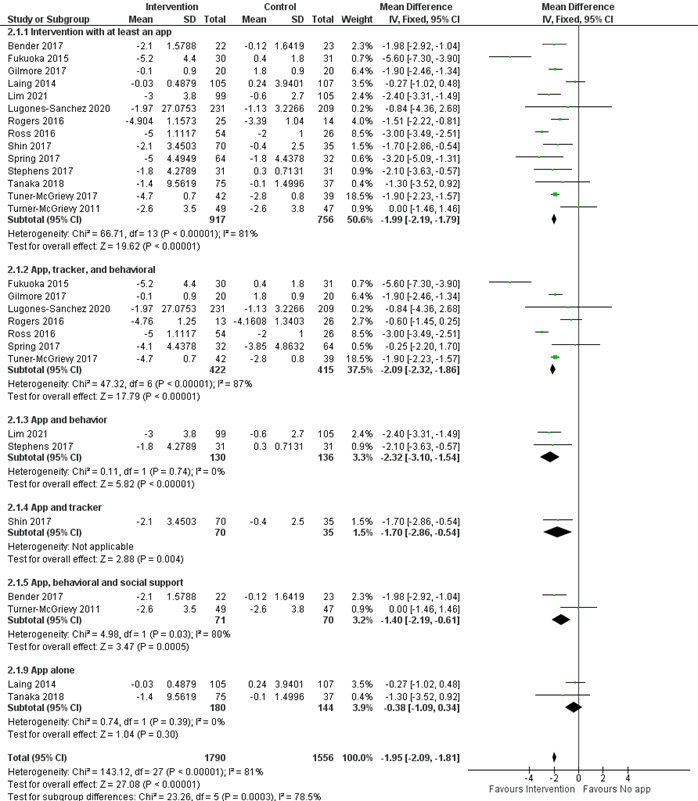
Forest plot of the effectiveness of mobile phone apps and the additional interventions on weight loss at 3 months [27,28,32,33,36,39-42,44,48,52,58,60]. IV: inverse variance method.

**Figure 4:**
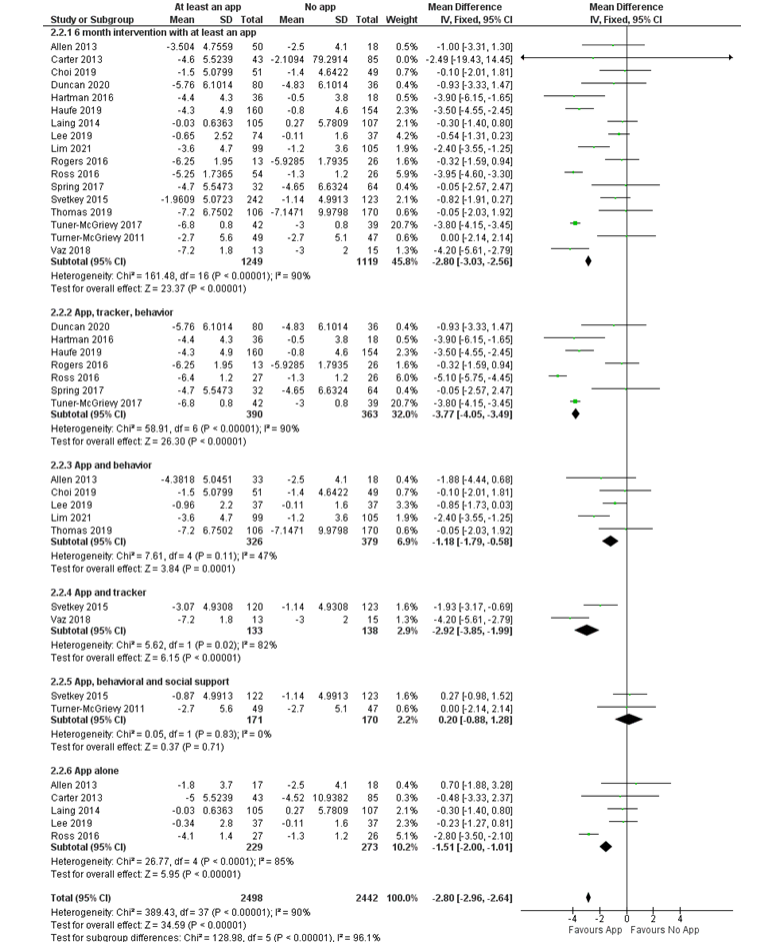
Forest plot of the effectiveness of mobile phone apps and the additional interventions on weight loss at 6 months [12,27-29,35-37,39,43,46-48,51,52,54,57,58]. IV: inverse variance method.

**Figure 5:**
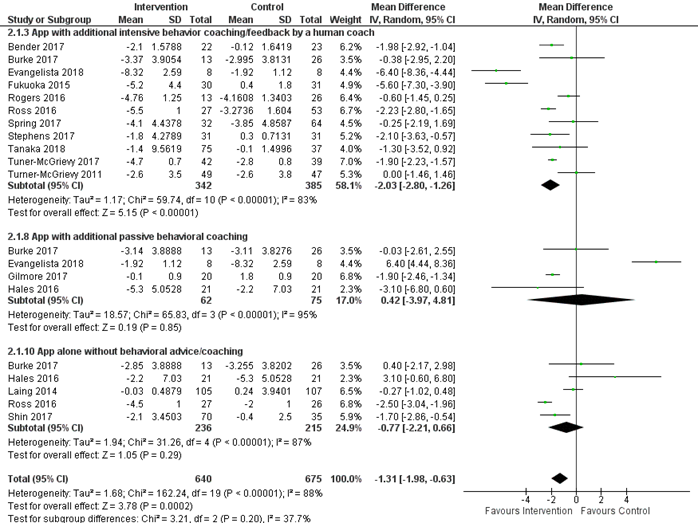
Subgroup analysis based on human-based versus passive behavioral interventions in combination with mobile app at 3 months [27,28,32,33,36,42,44,48,50,52,55]. IV: inverse variance method.

**Figure 6:**
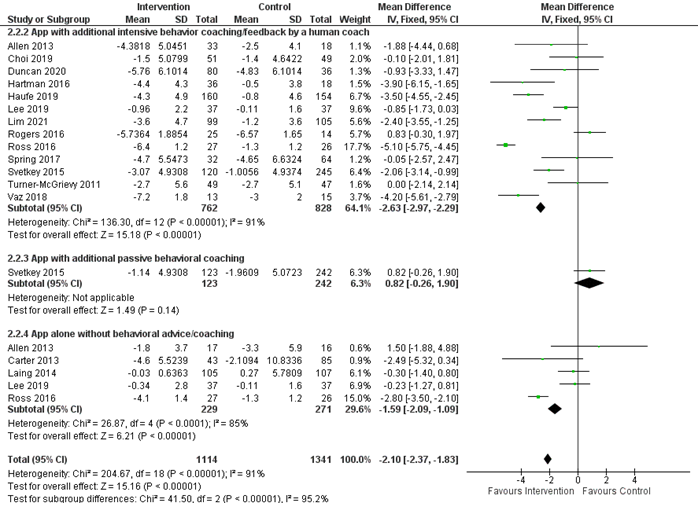
Subgroup analysis based on human-based versus passive behavioral interventions in combination with mobile app at 6 months [27-29,35,36,43,46,47,51,52,54,57]. IV: inverse variance method.

Of the 34 included studies, 24 (71%) examined the effectiveness of smartphone apps on weight loss at 3 and 6 months, whereas 5 (15%) measured the outcomes at 12 months; of these 5, 2 (40%) did not include SD or 95% CI [49,56] and 1 (20%) included an app in all arms [53]. Consequently, the meta-analysis and subgroup analysis were performed for the 3- and 6-month outcomes. Compared with the control group, smartphone apps resulted in a pooled net estimate weight loss of –1.99 kg (95% CI –2.19 to –1.79 kg; *I*^2^=81%) and –2.80 kg (95% CI –3.03 to –2.56 kg; *I*^2^=90%) at 3 and 6 months, respectively (Figure 3 [27,28,32,33,36,39-42,44,48,52,58,60] and Figure 4 [12,27-29,35-37,39,43,46-48,51,52,54,57,58]). Subgroup analysis was performed based on the different interventions that accompanied the use of the mobile app. When compared with control, the combination of the mobile app, tracker, and behavioral interventions showed a statistically significant weight loss of –2.09 kg (95% CI –2.32 to –1.86 kg; *I*^2^=87%) and –3.77 kg (95% CI –4.05 to –3.49 kg; *I*^2^=90%) at 3 and 6 months, respectively (Figures 3 and 4). Another subgroup analysis was performed based on the type of behavioral interventions, human-based versus passive. When compared with control, only the combination of the mobile app with intensive behavior coaching or feedback by a human coach showed a statistically significant weight loss of –2.03 kg (95% CI –2.80 to –1.26 kg; *I*^2^=83%) and –2.63 kg (95% CI –2.97 to –2.29 kg; *I*^2^=91%) at 3 and 6 months, respectively (Figure 5 [27,28,32,33,36,42,44,48,50,52,55] and Figure 6 [27-29,35,36,43,46,47,51,52,54,57]). The funnel plots (Multimedia Appendix 3) were symmetrical, suggesting that there was no publication bias.

### Characteristics of the Components of Both the Intervention and Control Arms

The studies included a total of 50 arms with an app and 35 control arms. Table 3 presents the characteristics of the various components of both the intervention and control arms. The app alone was used in 18% (9/50) of the intervention arms, whereas social and feedback interventions were not common, and financial incentives were the least used. The two most common combinations of interventions included the app, behavior component, and tracker (10/50, 20%) and app and behavior component (8/50, 16%). Among the 35 control arms, the behavior component was present in 21 (60%) and self-monitoring in 10 (29%), whereas no action was present in only 4 (11%). Multimedia Appendix 4 shows the details of the various components by study.

**Table 3:** Characteristics of the various components of both the intervention and control arms.

Components	Intervention arms (N=50), n (%)^a^	Control arms (N=35), n (%)^a^
App	50 (100)	N/A^b^
Behavior	32 (64)	21 (60)
Tracker	24 (50)	5 (14)
Social	10 (20)	2 (6)
Feedback	8 (16)	5 (14)
Financial incentives	2 (4)	0 (0)
Meal replacement	1 (2)	1 (3)
Self-monitoring	50 (100)	10 (29)
Usual care or waitlist	N/A	7 (20)

^a^Sum is more than 100% because arms could have more than 1 component.

^b^N/A: not applicable.

### Features of the Mobile App and Weight Loss

Commercial mobile apps were used in 56% (28/50) of the study arms. Key features of the mobile apps included self-monitoring (50/50, 100%), feedback (20/50, 40%), education (15/50, 30%), social support (12/50, 24%), rewards (7/50, 14%), and gamification (3/50, 6%; Table 4). Two-thirds of the mobile apps included 1-2 features. There was no association between weight loss and any specific feature; neither was there an association between weight loss and the number of app features (data not shown).

**Table 4:** Frequency of app features (N=50).

			Frequency, n (%)
**Feature**			
	Self-monitoring	50 (100)	
	Education	15 (30)	
	Feedback	20 (40)	
	Social support	12 (24)	
	Rewards	7 (14)	
	Gamification	3 (6)	
**Number of features per app**			
	1	22 (44)	
	2	9 (18)	
	3	12 (24)	
	4	7 (14)	

### Adherence to Smartphone Apps

In this meta-analysis, of the 34 included articles, 22 (65%) studied adherence to smartphone apps with diverse approaches to assessing adherence (Multimedia Appendix 5), leading to limitations in a direct comparison of their findings; thus, the results are described in a systematic review rather than a meta-analysis. Adherence data were extracted from each study, and a member of the research team (NA) coded the assessments based on the following four themes: (1) self-monitoring of weight, (2) self-monitoring of dietary intake, (3) self-monitoring of physical activity, and (4) interaction with the app. Each of these themes was defined differently in terms of measurement of adherence. Of the 22 articles that studied adherence to smartphone apps, 12 (55%) studies used more than one theme of adherence to weight loss apps; therefore, the total number of studies under each theme is not equal to the combined total number of studies included in this review.

Although dietary self-monitoring was the most commonly used adherence method among the studies (16/22, 73%), its measurement and analysis varied considerably. Studies defined adherence to dietary self-monitoring with a smartphone app either as recording any food or calorie intake [26,27,32,36,43,44,48,49,52,61] or a specific amount of calories [12,28,50] or a particular number of meals or entries [34,54] or both [37]. The frequency of dietary intake was based on the total number of days reported in percentages, ratios, or discrete numbers. Of the 22 studies, only 1 (5%) measured adherence as the percentage of participants logging food or calorie intake at least once per week [32]. The adherence rate to dietary intake ranged from 48% to 79% of the days when using self-monitoring.

App adherence for self-monitoring of weight and exercise was recorded in 36% (8/22) [27-29,32,37,38,49,54] and 27% (6/22) [27,28,36,44,52,54] of the studies, respectively. Common calculations included mean or percentage of daily recordings, recording at least once per week, or an average of days that participants recorded per week.

Of the 22 studies, 9 (41%) reported adherence as the frequency of the interaction with the app. Adherence was defined as wearing the wearable [32,33] or logging in [43,45] or opening the app, irrespective of the participant’s use [39]. The frequency of wearing the wearable ranged from at least 4-5 days per week [32,33] to at least 8 hours per day [33]. In contrast, a group of studies considered counting the daily interaction with specific app components or features as a sign of adherence [29,34,38,61]. For example, some considered completing entries immediately after taking photographs of the meal a sign of adherence [34].

Some studies reported positive associations between adherence and weight change by using combined adherence measurements [28], tracking adherence with dietary intake app recording [12,26,27,48], and measuring adherence with physical activity app recording [44]. In some of the studies [29,33,34,50,52], the adherence percentage showed evident decline throughout the study period.

## Discussion

### Principal Findings

This meta-analysis aims to measure the effectiveness of smartphone app–based interventions on weight loss, considering the additional components available in the mobile app. Similar to previous meta-analyses [13,18], the use of mobile apps resulted in a small significant weight loss of –2.03 kg (95% CI –2.57 to –1.5 kg; *I*^2^=83%) at 3 months. Although the mobile apps included different behavioral strategies, all relied on self-monitoring and only one-third included more than two features. It is important to note that there was no association between weight loss and mobile app features. A review of mobile app features revealed that self-monitoring was most commonly used, whereas social support and personalized feedback were less commonly used [62]. Subgroup analysis integrating additional nonapp intervention components with the mobile app showed that use of the tracker and behavioral components resulted in the most significant weight loss of –3.77 kg (95% CI –4.05 to –3.49 kg) at 6 months. Human-based behavioral interventions were associated with weight loss of –2.63 kg (95% CI –2.97 to –2.29 kg) at 6 months.

The various app features were not associated with weight loss. The meta-analysis has shown significant heterogeneity among the different apps used and the additional nonapp intervention components, reaching 90% in some forest plots. It is difficult to determine the role of a mobile app in weight loss management beyond self-monitoring. Only a few studies (7/36, 19%) based their work on theoretical frameworks such as social cognitive theory, transtheoretical models for health behavior change, and self-efficacy theory. Both the transtheoretical framework and self-efficacy theories rely on the individual, supporting the importance of self-monitoring as the main feature of mobile apps. Only social cognitive theory addresses the importance of support; the study results highlighted the importance of support by human coaches. The behavioral components of the included studies in this subgroup analysis were mainly in-person meetings [28,32,33,40], with additional feedback or telephone calls by a coach [28,36]. There were numerous smartphone app features such as self-monitoring with additional feedback [32,36,48], gamification and awards [33,40], and social support [28]. In the form of an in-person meeting or telephone call, the human component, in combination with the user app interactions through self-monitoring and feedback, is crucial for weight loss. The need for the human component raises the question of whether artificial intelligence would raise the mobile apps to a new level in the management of weight loss [63], such as the use of chatbots [64,65], and whether users would accept such a mode of coaching [66].

Weight maintenance is defined as losing 5%-10% of body weight and maintaining this loss for at least 1 year. In this meta-analysis, the studies analyzed were mainly short term and lasted for a maximum of 1 year. Of the 36 studies included in this systematic review, 2 (6%) had a longer intervention duration, and neither showed any difference in weight from baseline between the app-based intervention and the control at 18 months [37] and 24 months [29]. In a systematic review, Varkevisser et al [67] have provided strong evidence that behavioral determinants such as self-monitoring of weight and eating predict weight loss maintenance. Moreover, web-based interventions were effective for weight maintenance [68]. It would be helpful to examine further how mobile apps can be a form of self-regulation and adherence that can help users maintain weight loss. As obesity is a long-term relapsing disease and mobile apps are cost-effective, further research should address whether mobile apps could play a role in weight loss maintenance.

Some studies in this review and the literature [69-72] suggest that greater adherence to self-monitoring has been associated with greater weight loss. However, many articles do not provide detailed measurements of adherence to self-monitoring in weight loss apps. Furthermore, the results across studies could not be accurately compared because of the numerous variations in measurement methods and the definitions of themes used to assess adherence. Irrespective of the measurement method used, adherence to self-monitoring decreased with time, emphasizing the importance of studying different app features and associated interventions that could have affected the participants’ adherence. Of note, the variation in adherence measurements made it challenging to compare data across various studies. Hence, it is necessary to formulate a well-structured standard definition of adherence measures that can be used across future studies.

### Strengths and Limitations

To our knowledge, this is the only meta-analysis that has performed a subgroup analysis based on the add-on interventions to mobile apps. This meta-analysis also included gray literature such as conference abstracts, and the funnel plots showed good symmetry, excluding the possibility of publication bias. In contrast, the findings of this meta-analysis should be treated with caution because of the vast heterogeneity in the studies that would limit real-life applicability. Moreover, one-third of the articles had a high risk of bias; however, this bias could not have been avoided because of the nature of the app and its effect on blinding. Although some of the studies used commercial apps, the study team developed most of them. Another limitation is the heterogeneous behavioral component that ranged from simple booklets to in-person meetings and telephone calls. Finally, it is essential to note that the weight loss outcome was measured in kilograms rather than as a percentage of weight loss from baseline.

### Conclusions

Mobile phone apps have a role in weight loss management and result in modest weight loss compared with active control. Combining a mobile app, tracker, and human-delivered behavioral component led to the highest degree of weight loss at 6 months. Further research should use artificial intelligence to replace the human-delivered behavioral component for better mobile app use scalability.

## Appendix

Multimedia Appendix 1 Examples of the search strategy with keywords.

Multimedia Appendix 2 Summary of the excluded articles and the reasons for exclusion.

Multimedia Appendix 3 Funnel plots.

Multimedia Appendix 4 Summary of the components of the intervention and control arms of the included studies.

Multimedia Appendix 5 Summary of the adherence measures.

## Abbreviations

PRISMA: Preferred Reporting Items for Systematic Reviews and Meta-Analyses
