# Supplementary material for: The Effectiveness of Combining Nonmobile Interventions With the Use of Smartphone Apps With Various Features for Weight Loss: Systematic Review and Meta-analysis
Source: JMIR Mhealth Uhealth. 2022 Apr 8;10(4):e35479. doi: 10.2196/35479 (PMC9034427; doi:10.2196/35479)
Supplement: Multimedia Appendix 1 [file mhealth_v10i4e35479_app1.docx]

Multimedia Appendix 1: Examples of the search strategy with keywords

(((((("Smartphone"[Mesh]) OR "Cell Phone"[Mesh]) OR "Telemedicine"[Mesh]) OR ("Overweight"[Mesh]) OR "Obesity"[Mesh]) OR "Weight Loss"[Mesh]) OR "Weight Reduction Programs"[Mesh]

(“Smartphone"[Mesh] AND “Overweight"[Mesh]) OR (“Smartphone"[Mesh] AND "Obesity"[Mesh]) OR (“Smartphone”[Mesh] AND “ Weight Loss"[Mesh]) OR (“Smartphone”[Mesh] AND “ Weight Reduction Programs"[Mesh]) OR (“Cell Phone"[Mesh]” AND “Overweight"[Mesh]) OR (“Cell Phone"[Mesh]” AND "Obesity"[Mesh]) OR (“Cell Phone"[Mesh] AND "Weight Loss"[Mesh]) OR (“Cell Phone”[Mesh] AND “ Weight Reduction Programs"[Mesh])
