# Supplementary material for: The Effectiveness of Combining Nonmobile Interventions With the Use of Smartphone Apps With Various Features for Weight Loss: Systematic Review and Meta-analysis
Source: JMIR Mhealth Uhealth. 2022 Apr 8;10(4):e35479. doi: 10.2196/35479 (PMC9034427; doi:10.2196/35479)
Supplement: Multimedia Appendix 2 [file mhealth_v10i4e35479_app2.docx]

Multimedia Appendix 2: Summary of the excluded articles and the reason for exclusion

Reasons for excluded articles based on title/abstract

| Author, Date | No App | No weight loss as outcome | RCT |
| --- | --- | --- | --- |
| ("26th European Congress," 2019) | X |  |  |
| (Kennelly et al., 2019) | X |  |  |
| ("2013 SYR Accepted Poster Abstracts," 2013) | X |  |  |
| ("Abstracts: AAN 71st Annual Meeting," 2019) | X |  |  |
| (Zang et al., 2015) |  | X |  |
| (Manning et al., 2019) | X | X | X |
| (Adams et al., 2017) | X | X |  |
| (P. H. Lin et al., 2015) |  | X | X |
| (Adams et al., 2013) | X (text/email communication) |  |  |
| (Korinek et al., 2018) |  | X (steps) |  |
| (Lewis et al., 2019) | X ( text and phone call messages) |  |  |
| (Clark et al., 2018) | X (test messages) |  |  |
| (Mâsse et al., 2020) |  |  | X (protocol) |
| (Mensorio et al., 2019) | X (internet based program) |  |  |
| (Versteegden et al., 2018) |  |  | X (protocol) |
| (Hayot et al., 2019) |  |  | X (qualitative) |
| (Peter et al., 2012) | X |  |  |
| (Parekh et al., 2018) | X |  |  |
| (Manuvinakurike et al., 2014) |  | X | X |
| (Gorin et al., 2014) | X |  |  |
| (Sukumar et al., 2018) |  | X (exercise) | X (protocol) |
| (Olmos-Ochoa et al., 2019) | X (web based) | X |  |
| (Hutchesson et al., 2019) |  |  |  |
| (Graham Thomas & Bond, 2015) |  | X (walking) |  |
| (Bond et al., 2018) |  | X (migraine) |  |
| (Breyer et al., 2018) |  | X (urinary incontinence) |  |
| (Nicklas et al., 2016) |  |  | X |
| (Lewis et al., 2013) |  |  | X (protocol) |
| (Mayer et al., 2018) |  | X |  |
| (Beasley et al., 2019) |  |  | X (qualitative) |
| (Hawkes et al., 2010) | X (telephone based) | X |  |
| (Lycett et al., 2014) |  | X | X |
| (Fazzino et al., 2017) |  | X |  |
| (Newton et al., 2018) | X (text message) |  |  |
| (Merchant et al., 2014) | X (facebook internvetion) |  |  |
| (Handjieva-Darlenska et al., 2012) | X |  | X |
| (Ifejika et al., 2019) | X |  |  |
| (Kelly et al., 2020) | X ( text messages and phone calls) |  |  |
| (Rejeski et al., 2017) | X |  |  |
| (Appel et al., 2011) | X ( website, telephone |  |  |
| (Paranoan et al., 2018) | X |  |  |
| (Turner-McGrievy et al., 2013) | X (podcast) |  |  |
| (Reichard et al., 2015) | X |  |  |
| (Skau et al., 2016) |  |  | X (protocol) |
| (Fuller et al., 2014) | X |  |  |
| (Fleischman et al., 2016) | X ( tele visits) |  |  |
| (Pellegrini et al., 2018) | X |  |  |
| (Raynor et al., 2018) | X |  |  |
| (Steinberg, Levine, et al., 2013) | X (text messages) |  |  |
| (Shin et al., 2016) |  |  | X (design) |
| (Patrick et al., 2014) |  |  | X (protocol) |
| (Oreskovic et al., 2016) |  |  | X (protocol) |
| (Stein et al., 2019) |  |  | X (design) |
| (Scott et al., 2015) | X | X |  |
| (Price et al., 2015) | X (text messages) |  |  |
| (L. P. Smith et al., 2014) |  | X |  |
| (Faruqui et al., 2019) | X |  |  |
| (Stomby et al., 2015) | X |  |  |
| (Astbury et al., 2018) | X |  |  |
| (Warschburger & Zitzmann, 2019) | X |  |  |
| (Dennison et al., 2014) | X (web based) |  |  |
| (Harden et al., 2015) | X (internet based) |  |  |
| (Graham et al., 2017) | X (internet based) |  |  |
| (Mokhtari et al., 2018) | X (internet based) |  |  |
| (Morton et al., 2017) | X |  |  |
| (Helle et al., 2017) | X (website) |  |  |
| (Patel et al., 2019) |  | X |  |
| (Mason et al., 2019) | X |  |  |
| (Batra et al., 2013) | X |  |  |
| (Boh et al., 2016) |  |  | X (protocol) |
| (Ritzwoller et al., 2013) | X |  |  |
| (Rashad et al., 2019) | X |  |  |
| (Wang et al., 2018) | X (text and phone calls) | X (knee pain) |  |
| (Nelligan et al., 2019) | X (text messages) |  | X (protocol) |
| (De Niet, Timman, Bauer, Van Den Akker, Buijks, et al., 2012) | X (text messages) |  |  |
| (Livingstone et al., 2016) | X (internet based) |  |  |
| (Tene et al., 2018) | X |  |  |
| (Spadaro et al., 2017) | X |  |  |
| (Palmeira et al., 2019) | X (telephone calls) |  |  |
| (Chan et al., 2019) | X (text messages) |  |  |
| (Madjd et al., 2016) |  | X (exercise) |  |
| (Motie et al., 2017) | X |  |  |
| (Oshima et al., 2013) | X |  |  |
| (Williams et al., 2018) | X (telephone calls) |  |  |
| (Sangster et al., 2015) | X (telephone calls) |  |  |
| (Harries et al., 2016) |  | X (steps) |  |
| (Almeida et al., 2015) | X (email based) |  |  |
| (Rubinstein et al., 2016) | X (text messages and phone calls) |  |  |
| (Goulis et al., 2004) | X |  |  |
| (Wongrochananan et al., 2013) | X (text messages and email) |  |  |
| (Kim & Kim, 2008) |  | X (Diabetes) | X |
| (Hernández-Reyes, Molina-Recio, et al., 2020) |  |  | X (protocol) |
| (Palacios et al., 2018) |  | X (grocery) |  |
| (Kinoshita et al., 2014) | X |  |  |
| (Moldovan et al., 2016) | X |  |  |
| (Llanos et al., 2014) | X |  |  |
| (Järvelä-Reijonen et al., 2018) | X | X |  |
| (Swift et al., 2016) | X |  |  |
| (Knäuper et al., 2018) | X |  |  |
| (Volkova et al., 2014) | X |  |  |
| (Bachman & Raynor, 2012) | X |  |  |
| (King et al., 2016) |  | X (exercise) |  |
| (Østbye et al., 2016) |  | X |  |
| (Lindsay et al., 2019) | X |  |  |
| (Peters et al., 2014) | X |  |  |
| (Steinberg, Tate, et al., 2013) | X (emails) |  |  |
| (Chang et al., 2018) | X |  |  |
| (Young et al., 2017) | X (text messages and email) |  |  |
| (Limaye et al., 2017) | X (text messages) |  |  |
| (Martin et al., 2010) | X |  |  |
| (Timpel et al., 2018) |  |  | X (protocol) |
| (Kornman et al., 2010) | X (text messages) |  |  |
| (Pigeyre et al., 2016) | X |  |  |
| (Braden et al., 2016) | X |  |  |
| (Fanning et al., 2018) |  |  | X (protocol) |
| (West et al., 2019) | X (videos) |  |  |
| (Lavu et al., 2019) | X |  |  |
| (Graham et al., 2019) | X |  |  |
| (Donnelly et al., 2013) | X (phone calls) |  |  |
| (Yang et al., 2016) | X |  |  |
| (Rieder et al., 2013) | X |  |  |
| (Sidhu et al., 2016) | X (text messages) |  |  |
| (Helle et al., 2019) |  | X |  |
| (Unick et al., 2014) | X |  |  |
| (Armitage et al., 2014) | X |  |  |
| (Palmeira et al., 2017) | X (web based) |  |  |
| (Podina et al., 2017) |  |  | X (design) |
| (Recio-Rodriguez et al., 2018) |  |  | X (design) |
| (Schierberl Scherr et al., 2013) | X |  |  |
| (Duncan et al., 2018) |  |  | X (protocol) |
| (Raynor et al., 2019) |  | X |  |
| (Pawalia et al., 2017) |  | X |  |
| (Soureti et al., 2011) | X (text messages and web based) |  |  |
| (Werk et al., 2019) | X (telemedicine) |  |  |
| (Ekambareshwar et al., 2018) | X (telephone calls) |  |  |
| (Hadžiabdić et al., 2015) |  |  | X |
| (Al-Anqodi et al., 2018) |  | X (steps) |  |
| (Guerra et al., 2019) |  |  | X |
| (Haddock et al., 2014) |  |  |  |
| (Mendoza et al., 2017) | X (facebook) |  |  |
| (Burmeister et al., 2013) | X |  |  |
| (Wadden et al., 2011) | X |  |  |
| (Norman et al., 2013) |  |  |  |
| (Hansel et al., 2017) | X (web based) |  |  |
| (Garcia et al., 2018) | X (telephone calls) |  |  |
| (Svetkey et al., 2014) | X (internet based) | X |  |
| (Stewart et al., 2017) | X (website) |  |  |
| (Trief et al., 2016) | X (telephone calls) |  |  |
| (Russell-Minda et al., 2009) | X |  | X |
| (Sevick et al., 2018) |  |  | X |
| (Melchart et al., 2017) |  | X |  |
| (Nyström et al., 2015) | X | X |  |
| (Zwickert et al., 2016) | X (text messages) |  |  |
| (Rosas et al., 2018) | X (online videos) |  |  |
| (Staiano et al., 2018) | X (video gaming) |  |  |
| (Davis et al., 2019) |  |  | X (design) |
| (Tyson et al., 2013) |  | X |  |
| (Bui et al., 2019) | X | X |  |
| (Lee et al., 2017) | X | X |  |
| (Coughlin et al., 2016) | X |  |  |
| (Blomfield et al., 2014) | X (website) |  |  |
| (Krukowski et al., 2019) | X (online group) |  |  |
| (Martinez-Brockman et al., 2017) | X (text messages |  |  |
| (Holland-Carter et al., 2017) |  | X (psychological problems) |  |
| (Fernandez-Luque et al., 2017) |  | X | X |
| (Partridge et al., 2017) | X | X |  |
| (Collins et al., 2013) |  | X |  |
| (Choi et al., 2018) |  | X |  |
| (Jaén et al., 2019) |  | X |  |
| (Suso-Ribera et al., 2018) |  | X |  |
| (Sherry et al., 2018) | X |  |  |
| (Bennett et al., 2014) |  |  | X |
| (Spring et al., 2013) | X (personal assistant device) |  |  |
| (Hammersley et al., 2019) | X (internet based) |  |  |
| (Little et al., 2016) | X (web based) |  |  |
| (Mummah et al., 2016) |  |  | X |
| (West et al., 2011) | X |  |  |
| (Weinstock et al., 2011) | X (home videos) |  |  |
| (Ahmed et al., 2018) |  |  | X |
| (Stuckey et al., 2013) |  |  | X (protocol) |
| (Abraham et al., 2015) | X (text messages and phone calls) |  |  |
| (Niklowitz et al., 2018) | X |  |  |
| (Romero-Moraleda et al., 2015) |  | X (lipid) |  |
| (Theissing et al., 2013) | X |  |  |
| (Eakin et al., 2014) | X (phone calls) |  |  |
| (Stumm et al., 2016) | X (telemedicine) |  |  |
| (Pekkarinen et al., 2015) |  | X |  |
| (Carson et al., 2017) | X |  |  |
| (S. S. Martin et al., 2015) |  | X (exercise) |  |
| (Berli et al., 2018) |  | X (exercise) |  |
| (Lubans, Smith, Morgan, et al., 2016) |  | X (psychological well being) |  |
| (Senarath et al., 2019) | X (web based) |  |  |
| (Nezami et al., 2018) | X (text messages and web based) |  |  |
| (Miller et al., 2017) | X |  |  |
| (Finkelstein et al., 2015) |  | X (exercise) |  |
| (Schultz et al., 2013) | X (text messages) |  |  |
| (Lozano-Lozano et al., 2019) |  | X |  |
| (Willcox et al., 2017) |  | X |  |
| (Cherasard et al., 2018) | X |  |  |
| (Shaw et al., 2013) | X |  |  |
| (Petrella et al., 2014) | X | X |  |
| (Griauzde et al., 2018) |  |  | X (protocol) |
| (Fred Wen et al., 2019) | X |  |  |
| (Lim et al., 2016) | X |  |  |
| (Banna et al., 2017) | X (text messages) |  |  |
| (Höchsmann et al., 2019) |  | X |  |
| (Evans et al., 2015) |  |  | X (protocol) |
| (Wong et al., 2020) |  |  | X (protocol) |
| (Luque et al., 2019) | X |  |  |
| (Ahn & Choi, 2016) | X (text messages) |  |  |
| (Wister et al., 2007) | X (telehealth) |  |  |
| (Chai et al., 2019) | X (telehealth) |  |  |
| (Pellegrini et al., 2014) |  |  | X (protocol) |
| (Patrick et al., 2013) | X (website) |  |  |
| (Dalton et al., 2013) |  | X |  |
| (Cleo et al., 2018) |  |  | X (qualitative) |
| (Marcus et al., 2016) | X (internet) | X |  |
| (Funk et al., 2018) |  | X |  |
| (Wu et al., 2010) |  |  | X (qualitative) |
| (Aschbrenner et al., 2018) |  |  | X (protocol) |
| (Faghri et al., 2017) |  | X |  |
| (Rafols et al., 2014) | X |  |  |
| (van Grieken et al., 2017) | X (web based) |  |  |
| (Webber & Rose, 2013) | X (internet) |  |  |
| (Cheung et al., 2019) | X (text messages) |  |  |
| (Marra et al., 2019) | X (telephone call) |  |  |
| (Ventura Marra et al., 2019) | X (tele nutrition) |  |  |
| (Cassin et al., 2016) | X (telephone calls) | X |  |
| (Martin et al., 2018) | X (telephone calls)(Vinter et al., 2014) |  |  |
| (Vinter et al., 2014) | X |  |  |
| (Neumeier et al., 2017) | X |  |  |
| (Priscilla et al., 2015) | X |  |  |
| (Steegers-Theunissen, 2018) | X |  |  |
| (Santos et al., 2015) | X |  |  |
| (González et al., 2013) | X (website) |  |  |
| (Kennelly et al., 2018) |  | X |  |
| (Kennelly et al., 2016) | X |  |  |
| (Small et al., 2014) | X |  |  |
| (Parker et al., 2018) |  |  | X |
| (Sakane et al., 2019) | X (telephone calls) |  |  |
| (Partridge, Allman-Farinelli, et al., 2016) |  | X |  |
| (Gillison et al., 2015) | X |  |  |
| (Hult et al., 2018) |  |  | X (protocol) |
| (Sutherland, Brown, et al., 2019) | X |  |  |
| (Brantley et al., 2014) | X |  |  |
| (McRobbie et al., 2019) | X |  |  |
| (Shrewsbury et al., 2009) | X |  |  |
| (Ng et al., 2018) |  | X |  |
| (M. Kennelly et al., 2017) |  | X |  |
| (Fogarty et al., 2015) | X |  |  |
| (Lauti et al., 2018) | X (text messages) |  |  |
| (Leahey & Wing, 2013) | X |  |  |
| (Dutton et al., 2015) | X |  |  |
| (Gorin et al., 2018) | X |  |  |
| (Onoue et al., 2018) | X |  |  |
| (Becofsky et al., 2017) | X (internet) |  |  |
| (Wagner et al., 2016) | X (internet) |  |  |
| (Pagoto et al., 2013) | X |  |  |
| (Shikany et al., 2013) | X |  |  |
| (Sutherland, Nathan, et al., 2019) | X |  |  |
| (Tate et al., 2017) | X |  |  |
| (Dutton et al., 2014) | X |  |  |
| (Cadmus-Bertram et al., 2015) | X |  |  |
| (Haire-Joshu et al., 2018) |  |  | X |
| (Nezami et al., 2016) | X (text messages and website) |  |  |
| (Joseph et al., 2019) |  |  | X (protocol) |
| (Teong et al., 2020) |  |  | X (protocol) |
| (J. J. Smith, P. J. Morgan, R. C. Plotnikoff, K. A. Dally, J. Salmon, A. D. Okely, T. L. Finn, M. J. Babic, et al., 2014) |  |  | X (protocol) |
| (Wilczynska et al., 2016) |  |  | X (protocol) |
| (Pekmezi et al., 2017) |  |  | X (protocol) |
| (Partridge et al., 2014) | X |  |  |
| (Goldstein et al., 2020) | X |  |  |
| (Goode et al., 2015) | X (telephone calls) |  |  |
| (Ptomey et al., 2018) |  |  | X (protocol) |
| (Bollyky et al., 2018) | X |  |  |
| (Cato et al., 2014) | X |  |  |
| (Phelan et al., 2019) | X (internet) |  |  |
| (Laitner et al., 2016) | X |  |  |
| (VanWormer et al., 2010) | X |  |  |
| (Liu et al., 2019) |  |  | X |
| (Sweeney et al., 2019) |  |  | X |
| (Xu et al., 2017) | X |  |  |
| (Oshakbayev et al., 2019) | X |  |  |
| (Morgan et al., 2010) |  |  | X |
| (de Niet, Timman, Bauer, van den Akker, de Klerk, et al., 2012) | X (text messages) |  |  |
| (Yank et al., 2014) | X |  |  |
| (Truby et al., 2019) |  |  | X (protocol) |
| (Lutes et al., 2012) | X (phone calls) |  |  |
| (Ek et al., 2018) |  | X (exercise) |  |
| (Glynn et al., 2013) |  |  | X (protocol) |
| (Lim et al., 2019) |  |  | X (protocol) |
| (Pfammatter et al., 2019) |  |  | X (protocol) |
| (Burke et al., 2020) |  |  | X (protocol) |
| (O'Malley et al., 2014) |  |  |  |
| (J. J. Smith, P. J. Morgan, R. C. Plotnikoff, K. A. Dally, J. Salmon, A. D. Okely, T. L. Finn, & D. R. Lubans, 2014) | X (website) |  |  |
| (Ipjian & Johnston, 2017) |  | X |  |
| (Goswami et al., 2019) | X |  |  |
| (Sit et al., 2016) |  | X (exercise) |  |
| (Varnfield et al., 2014) | X |  |  |
| (Pellegrini et al., 2012) |  |  | X (protocol) |
| (Fiks et al., 2017) | X (facebook) |  |  |
| (Kiernan et al., 2012) | X |  |  |
| (Holmen et al., 2016) | X |  |  |
| (Castelnuovo, Manzoni, Villa, et al., 2011) |  |  | X (protocol) |
| (Henriksen et al., 2014) | X |  |  |
| (Voils et al., 2018) |  |  | X (protocol) |
| (Matthews et al., 2017) |  |  | X (protocol) |
| (Milsom et al., 2011) |  |  | X |
| (Pieniak et al., 2016) |  |  | X |
| (Ifejika et al., 2016) |  |  | X (protocol) |
| (Kjær et al., 2018) | X (telephone and email) |  |  |
| (M. Lin et al., 2015) | X (text messages) |  |  |
| (Kim & Song, 2008) | X (text messages and internet) |  |  |
| (Mousa et al., 2017) | X (telehealth) |  |  |
| (Sakakibara et al., 2014) | X (telehealth) |  |  |
| (Rimmer et al., 2013) | X (telehealth) |  |  |
| (O'Brien et al., 2018) | X (telephone calls) |  |  |
| (Lee et al., 2014) | X (telephone calls) |  |  |
| (Stuart et al., 2014) | X (telephone calls) |  |  |
| (Lyons et al., 2016) |  |  | X (protocol) |
| (Willcox et al., 2015) |  |  | X (protocol) |
| (Shapiro et al., 2012) | X (text messages) |  |  |
| (Fischer et al., 2016) | X (text messages) |  |  |
| (Patrick et al., 2009) | X (text messages) |  |  |
| (Silina et al., 2017) | X (text messages) |  |  |
| (Godino et al., 2019) | X (text messages) |  |  |
| (Axley et al., 2017) | X (text messages) |  |  |
| (Axley et al., 2018) | X (text messages) |  |  |
| (Lin et al., 2014) | X (text messages) |  |  |
| (Armstrong et al., 2018) | X (text messages) |  |  |
| (Jiskoot et al., 2017) |  |  | X (protocol) |
| (Hammersley et al., 2017) |  |  | X (protocol) |
| (Samuel-Hodge et al., 2013) | X |  |  |
| (Varnfield et al., 2016) | X (telemedicine) |  |  |
| (Davis et al., 2016) | X (telemedicine) |  |  |
| (Gallagher et al., 2011) | X (telemedicine) |  |  |
| (Davis et al., 2013) | X (telemedicine) |  |  |
| (Lindsay et al., 2018) |  | X |  |
| (Hebden et al., 2013) |  |  | X (protocol) |
| (Yoo et al., 2009) |  | X |  |
| (Ambeba et al., 2015) | X |  |  |
| (Bala et al., 2019) | X (text message and video calls) |  |  |
| (Davis et al., 2011) |  |  | X |
| (Napolitano et al., 2013) | X (facebook and text messages) |  |  |
| (Guan et al., 2015) |  | X (steps) |  |
| (Burke et al., 2012) | X |  |  |
| (Napolitano et al., 2017) |  |  | X |
| (Gerber et al., 2013) | X |  |  |
| (Abrahamsson et al., 2018) | X |  |  |
| (Azar et al., 2015) | X |  |  |
| (Hageman et al., 2017) | X (web based) |  |  |
| (Ashwell et al., 2014) | X (web based) |  |  |
| (Swencionis et al., 2013) |  | X |  |
| (Luley et al., 2014) | X |  |  |
| (Luley et al., 2011) | X |  |  |
| (Thomas et al., 2017) | X |  |  |
| (Mason et al., 2018) | X |  |  |
| (Ahern et al., 2014) |  |  | X |
| (Turner-McGrievy & Tate, 2013) | X (social media) |  |  |
| (Rock et al., 2014) | X |  |  |
| (Donnelly et al., 2016) | X (facetime) |  |  |
| (Barnason et al., 2019) | X (telehealth) |  |  |
| (Geiker et al., 2016) | X |  |  |
| (Murphy & Williams, 2013) |  |  | X |
| (Pollak et al., 2014) | X (text messages) |  |  |
| (Kumagai et al., 2018) | X |  |  |
| (Madigan et al., 2014) | X |  |  |
| (Fuller et al., 2013) | X |  |  |
| (Bond et al., 2017) | X |  |  |
| (Whittemore et al., 2019) | X (text messages) |  |  |
| (Nikolaou & Lean, 2017) | X |  |  |

Records excluded based on full-text

| Author | No mobile app intervention | No weight loss as outcome | Non RCT |
| --- | --- | --- | --- |
| ("Eight-year weight losses with an intensive lifestyle intervention: the look AHEAD study," 2014) | X |  |  |
| (Abraham et al., 2015) | X (video based) |  |  |
| (Abrams et al., 2013) | X |  |  |
| (Ainscough, Kennelly, Lindsay, et al., 2017) |  | X |  |
| (Ainscough et al., 2016) |  | X |  |
| (Ainscough, Kennelly, O'Sullivan, et al., 2017) |  | X |  |
| (Ainscough et al., 2018) |  | X |  |
| (Ainscough et al., 2020) |  | X |  |
| (Alencar et al., 2020) | X (website) |  |  |
| (Alencar et al., 2019) | X |  |  |
| (Alonso-Domínguez et al., 2019) |  | X |  |
| (Altazan et al., 2019) |  | X |  |
| (Annesi et al., 2016) | X |  |  |
| (Aschbrenner et al., 2019) |  | X |  |
| (Bartholomew et al., 2015) |  | X |  |
| (Batch et al., 2014) |  | X |  |
| (Bond et al., 2013) | X |  |  |
| (Bosworth et al., 2018) | X (telephone calls) |  |  |
| (Brown et al., 2014) | X |  |  |
| (Byrne et al., 2018) | X (tablet) |  |  |
| (Carels et al., 2014) | X |  |  |
| (Carter et al., 2015) |  | X |  |
| (Castelnuovo, Manzoni, Cuzziol, et al., 2011) |  | X |  |
| (Castelnuovo et al., 2010) |  | X |  |
| (Cattivelli et al., 2018) |  |  | X |
| (Chen et al., 2019) | X |  |  |
| (Chow et al., 2019) | X |  |  |
| (Cleo et al., 2019) | X |  |  |
| (Craigie et al., 2011) | X |  |  |
| (Crain et al., 2018) | X (phone calls) |  |  |
| (Crane et al., 2015) | X (phone calls) |  |  |
| (Creasy et al., 2018) | X (phone calls) |  |  |
| (de Vos et al., 2014) | x |  |  |
| (Delisle et al., 2015) |  |  | x |
| (Delisle Nyström et al., 2018) | X (web based) |  |  |
| (Desouza et al., 2012) | x |  |  |
| (Dodd et al., 2018) |  | X |  |
| (Dreyer Gillette et al., 2014) | X |  |  |
| (Eakin et al., 2010) | X (phone calls) |  |  |
| (Edney et al., 2019) |  | X (exercise) |  |
| (Evans et al., 2019) | X |  |  |
| (Ferrante et al., 2018) | X (website) |  |  |
| (Fisher et al., 2013) | X |  |  |
| (Foley et al., 2016) | X |  |  |
| (Forman et al., 2019) |  | X |  |
| (Forman et al., 2016) | X |  |  |
| (Frisch et al., 2009) | X (phone calls) |  |  |
| (Fukuoka et al., 2014)  Already included; this is the conference abstract of the study |  |  |  |
| (Fukuoka et al., 2018) |  |  | X |
| (Goldstein et al., 2020) | X |  |  |
| (Granado-Font et al., 2015) |  |  | X |
| (Greene et al., 2018) |  | X |  |
| (Haapala et al., 2009) | X (text messages) |  |  |
| (Haggerty et al., 2017) | X |  |  |
| (Haggerty et al., 2016) | X |  |  |
| (Heldt et al., 2018) |  | X |  |
| (Hernández-Reyes, Cámara-Martos, et al., 2020) | X (text messages) |  |  |
| (Holmen et al., 2015) |  | X |  |
| (Holzapfel et al., 2014) | X (internet) |  |  |
| (Huang et al., 2014) | X (web based) |  |  |
| (Huber et al., 2015) | X (phone calls) |  |  |
| (Huseinovic et al., 2014) | X |  |  |
| (Hutchesson et al., 2016) | X (web based) |  |  |
| (Ifejika et al., 2016) |  |  | X |
| (Ienca et al., 2018) |  |  | X |
| (Jeffery et al., 2003) | X |  |  |
| (Johansson & Danielsson, 2019) | X (web based) |  |  |
| (Johnston et al., 2019) | X |  |  |
| (Kakoschke et al., 2018) |  | X |  |
| (Kakoschke et al., 2019) | X |  |  |
| (M. A. Kennelly et al., 2017) |  | X |  |
| (Kim et al., 2019b) |  | X |  |
| (Kim et al., 2019a) |  | X |  |
| (Kurscheid et al., 2019) |  | X |  |
| (L'Allemand et al., 2018) |  | X |  |
| (Lawrence et al., 2018) | X |  |  |
| (Lee et al., 2018) | X |  |  |
| (Lewis, 2014) |  |  | X |
| (Lubans, Smith, Plotnikoff, et al., 2016) |  |  | X |
| (C. K. Martin et al., 2015) | X |  |  |
| (Martineta et al., 2019) |  | X |  |
| (Memon et al., 2018) | X |  |  |
| (Morgan et al., 2014) | X (website) |  |  |
| (Morrison et al., 2014) |  |  | X |
| (Mummah et al., 2017) |  | X |  |
| (Naets et al., 2020) | X |  |  |
| (Nakade et al., 2012) | X |  |  |
| (O'Brien et al., 2014) | X (web based) |  |  |
| (Oddone et al., 2018) | X (phone calls) |  |  |
| (Oddsson, 2017) |  |  | X |
| (Olson et al., 2018) |  | X |  |
| (Olson et al., 2017) |  | X |  |
| (O'Neil et al., 2012) | X |  |  |
| (O'Neill et al., 2016) |  | X |  |
| (Orsama et al., 2013) |  | X |  |
| (Padhye & Jing, 2016) |  | X |  |
| (Partridge, McGeechan, et al., 2016) |  | X |  |
| (Patrick et al., 2014) |  |  | X |
| (Petrella et al., 2017) | X |  |  |
| (Pinto et al., 2013) | X |  |  |
| (Sasai et al., 2017) | X |  |  |
| (Sherwood et al., 2006) | X (phone calls) |  |  |
| (Sniehotta et al., 2019) | X (text messages) |  |  |
| (Steinberg et al., 2014) | X |  |  |
| (Tanaka et al., 2010) | X |  |  |
| (Timpel et al., 2018) |  |  | X |
| (Turner-McGrievy et al., 2014) | X |  |  |
| (Turner-McGrievy & Tate, 2014) |  | X |  |
| (Van Horn et al., 2018) |  | X |  |
| (Versteegden, Scheerhoorn, et al., 2019) |  |  | X |
| (Versteegden, Van Himbeeck, et al., 2019) | X |  |  |
| (Vidmar et al., 2019) |  |  | X |
| (Voils et al., 2017) | X |  |  |
| (Widmer et al., 2017) | X |  |  |
| (Willcox et al., 2017) |  | X |  |
| (Wipfli et al., 2019) | X (web based) |  |  |
| (Yancy et al., 2018) | X |  |  |
| (Yank et al., 2013) | X |  |  |

**List of Articles**

26th European Congress. (2019). [Conference Review]. *Obes Facts*, *12*. http://www.embase.com/search/results?subaction=viewrecord&from=export&id=L628194312

2013 SYR Accepted Poster Abstracts. (2013). [Article]. *International journal of yoga therapy*, *23*, 32-53. http://www.embase.com/search/results?subaction=viewrecord&from=export&id=L611467725

Abraham, A. A., Chow, W. C., So, H. K., Yip, B. H., Li, A. M., Kumta, S. M., Woo, J., Chan, S. M., Lau, E. Y., & Nelson, E. A. (2015). Lifestyle intervention using an internet-based curriculum with cell phone reminders for obese Chinese teens: a randomized controlled study. *PLoS One*, *10*(5), e0125673. https://doi.org/10.1371/journal.pone.0125673

Abrahamsson, N., Ahlund, L., Ahrin, E., & Alfonsson, S. (2018). Video-based CBT-E improves eating patterns in obese patients with eating disorder: A single case multiple baseline study [Article]. *Journal of Behavior Therapy and Experimental Psychiatry*, *61*, 104-112. https://doi.org/10.1016/j.jbtep.2018.06.010

Abrams, P., Levitt Katz, L. E., Moore, R. H., Xanthopoulos, M. S., Bishop-Gilyard, C. T., Wadden, T. A., & Berkowitz, R. I. (2013). Threshold for improvement in insulin sensitivity with adolescent weight loss. *J Pediatr*, *163*(3), 785-790. https://doi.org/10.1016/j.jpeds.2013.04.003

Abstracts: AAN 71st Annual Meeting. (2019). [Conference Review]. *Neurology*, *92*(15). http://www.embase.com/search/results?subaction=viewrecord&from=export&id=L629492898

Adams, M. A., Hurley, J. C., Todd, M., Bhuiyan, N., Jarrett, C. L., Tucker, W. J., Hollingshead, K. E., & Angadi, S. S. (2017). Adaptive goal setting and financial incentives: a 2 × 2 factorial randomized controlled trial to increase adults' physical activity [Article]. *BMC Public Health*, *17*(1), 286. https://doi.org/10.1186/s12889-017-4197-8

Adams, M. A., Sallis, J. F., Norman, G. J., Hovell, M. F., Hekler, E. B., & Perata, E. (2013). An adaptive physical activity intervention for overweight adults: A randomized controlled trial [Article]. *PLoS One*, *8*(12). https://doi.org/10.1371/journal.pone.0082901

Ahern, A. L., Aveyard, P. N., Halford, J. C., Mander, A., Cresswell, L., Cohn, S. R., Suhrcke, M., Marsh, T., Thomson, A. M., & Jebb, S. A. (2014). Weight loss referrals for adults in primary care (WRAP): protocol for a multi-centre randomised controlled trial comparing the clinical and cost-effectiveness of primary care referral to a commercial weight loss provider for 12 weeks, referral for 52 weeks, and a brief self-help intervention [ISRCTN82857232]. *BMC Public Health*, *14*, 620. https://doi.org/10.1186/1471-2458-14-620

Ahmed, H. O., Hama Marif, M. A., sabah abid, a., Ali Omer, M. A., majeed nuri, D. A., Hamasur, A. F., Ahmed, S. H., & Abddalqadir, K. M. (2018). The life styles causing overweight or obesity: Based on 5 years of experience in two centers in Sulaimani Governorate, Kurdistan Region/Iraq [Article]. *International Journal of Surgery Open*, *11*, 22-29. https://doi.org/10.1016/j.ijso.2018.04.002

Ahn, A., & Choi, J. (2016). A one-way text messaging intervention for obesity [Article]. *J Telemed Telecare*, *22*(3), 148-152. https://doi.org/10.1177/1357633X15591129

Ainscough, K., Kennelly, M., Lindsay, K., ÓSullivan, E., Gibney, E., & McAulife, F. (2017). The impact of an mhealth-supported lifestyle intervention on dietary and physical activity outcomes among overweight and obese pregnant women: A randomised controlled trial [Conference Abstract]. *Obes Facts*, *10*, 211-212. https://doi.org/10.1159/000468958

Ainscough, K., Kennelly, M., Lindsay, K. L., O'Sullivan, E. J., & McAuliffe, F. M. (2016). Impact of an mHealth supported healthy lifestyle intervention on behavioural stage of change in overweight and obese pregnancy [Conference Abstract]. *Proceedings of the Nutrition Society*, *75*(OCE3), E85. https://doi.org/10.1017/S0029665116001002

Ainscough, K., Kennelly, M., O'Sullivan, E., Lindsay, K., & McAuliffe, F. (2017). Impact of an mHealth-supported behavioural lifestyle intervention on exercise stage-of-change and physical activity in overweight and obese pregnancy: PEARs randomised controlled trial (RCT) [Conference Abstract]. *BJOG: An International Journal of Obstetrics and Gynaecology*, *124*, 96-97. https://doi.org/10.1111/1471-0528.14588

Ainscough, K., Kennelly, M. A., O'Sullivan, E. J., Lindsay, K. L., Gibney, E. R., McCarthy, M., & McAuliffe, F. M. (2018). Impact of a smartphone app supporting a lifestyle intervention in overweight and obese pregnancy on on maternal health and lifestyle outcomes [Conference Abstract]. *American Journal of Obstetrics and Gynecology*, *218*(1), S598-S599. http://www.embase.com/search/results?subaction=viewrecord&from=export&id=L620310323

Ainscough, K. M., O'Brien, E. C., Lindsay, K. L., Kennelly, M. A., O'Sullivan, E. J., O'Brien, O. A., McCarthy, M., De Vito, G., & McAuliffe, F. M. (2020). Nutrition, Behavior Change and Physical Activity Outcomes From the PEARS RCT—An mHealth-Supported, Lifestyle Intervention Among Pregnant Women With Overweight and Obesity [Article]. *Frontiers in Endocrinology*, *10*. https://doi.org/10.3389/fendo.2019.00938

Al-Anqodi, N., McCullough, F., & Salter, A. M. (2018). Feasibility of smartphone application to promote physical activity in healthy Omani female adults [Conference Abstract]. *Proceedings of the Nutrition Society*, *77*(OCE4). https://doi.org/10.1017/S0029665118001556

Alencar, M., Johnson, K., Gray, V., Mullur, R., Gutierrez, E., & Dionico, P. (2020). Telehealth-Based Health Coaching Increases m-Health Device Adherence and Rate of Weight Loss in Obese Participants [Article]. *Telemed J E Health*, *26*(3), 365-368. https://doi.org/10.1089/tmj.2019.0017

Alencar, M. K., Johnson, K., Mullur, R., Gray, V., Gutierrez, E., & Korosteleva, O. (2019). The efficacy of a telemedicine-based weight loss program with video conference health coaching support [Article]. *J Telemed Telecare*, *25*(3), 151-157. https://doi.org/10.1177/1357633X17745471

Almeida, F. A., You, W., Harden, S. M., Blackman, K. C., Davy, B. M., Glasgow, R. E., Hill, J. L., Linnan, L. A., Wall, S. S., Yenerall, J., Zoellner, J. M., & Estabrooks, P. A. (2015). Effectiveness of a worksite-based weight loss randomized controlled trial: the worksite study. *Obesity (Silver Spring)*, *23*(4), 737-745. https://doi.org/10.1002/oby.20899

Alonso-Domínguez, R., García-Ortiz, L., Patino-Alonso, M. C., Sánchez-Aguadero, N., Gómez-Marcos, M. A., & Recio-Rodríguez, J. I. (2019). Effectiveness of a multifactorial intervention in increasing adherence to the mediterranean diet among patients with diabetes mellitus type 2: A controlled and randomized study (EMID study) [Article]. *Nutrients*, *11*(1). https://doi.org/10.3390/nu11010162

Altazan, A. D., Redman, L. M., Burton, J. H., Beyl, R. A., Cain, L. E., Sutton, E. F., & Martin, C. K. (2019). Mood and quality of life changes in pregnancy and postpartum and the effect of a behavioral intervention targeting excess gestational weight gain in women with overweight and obesity: A parallel-arm randomized controlled pilot trial [Article]. *BMC Pregnancy Childbirth*, *19*(1). https://doi.org/10.1186/s12884-019-2196-8

Ambeba, E. J., Ye, L., Sereika, S. M., Styn, M. A., Acharya, S. D., Sevick, M. A., Ewing, L. J., Conroy, M. B., Glanz, K., Zheng, Y., Goode, R. W., Mattos, M., & Burke, L. E. (2015). The use of mHealth to deliver tailored messages reduces reported energy and fat intake [Article]. *J Cardiovasc Nurs*, *30*(1), 35-43. https://doi.org/10.1097/JCN.0000000000000120

Annesi, J. J., Mareno, N., & McEwen, K. (2016). Psychosocial predictors of emotional eating and their weight-loss treatment-induced changes in women with obesity. *Eat Weight Disord*, *21*(2), 289-295. https://doi.org/10.1007/s40519-015-0209-9

Appel, L. J., Clark, J. M., Yeh, H. C., Wang, N. Y., Coughlin, J. W., Daumit, G., Miller, E. R., 3rd, Dalcin, A., Jerome, G. J., Geller, S., Noronha, G., Pozefsky, T., Charleston, J., Reynolds, J. B., Durkin, N., Rubin, R. R., Louis, T. A., & Brancati, F. L. (2011). Comparative effectiveness of weight-loss interventions in clinical practice. *New England Journal of Medicine*, *365*(21), 1959-1968.

Armitage, C. J., Norman, P., Noor, M., Alganem, S., & Arden, M. A. (2014). Evidence that a very brief psychological intervention boosts weight loss in a weight loss program. *Behav Ther*, *45*(5), 700-707. https://doi.org/10.1016/j.beth.2014.04.001

Armstrong, S., Mendelsohn, A., Bennett, G., Taveras, E. M., Kimberg, A., & Kemper, A. R. (2018). Texting Motivational Interviewing: A Randomized Controlled Trial of Motivational Interviewing Text Messages Designed to Augment Childhood Obesity Treatment. *Child Obes*, *14*(1), 4-10. https://doi.org/10.1089/chi.2017.0089

Aschbrenner, K., Naslund, J., Gorin, A., Mueser, K., & Bartels, S. (2019). Randomized trial of a lifestyle intervention for young adults with serious mental illness in community mental health centers [Conference Abstract]. *Schizophrenia Bulletin*, *45*, S135. https://doi.org/10.1093/schbul/sbz022.114

Aschbrenner, K. A., Naslund, J. A., Gorin, A. A., Mueser, K. T., Scherer, E. A., Viron, M., Kinney, A., & Bartels, S. J. (2018). Peer support and mobile health technology targeting obesity-related cardiovascular risk in young adults with serious mental illness: Protocol for a randomized controlled trial. *Contemp Clin Trials*, *74*, 97-106.

Ashwell, M., Howarth, E., Chesters, D., Allan, P., Hoyland, A., & Walton, J. (2014). A web-based weight loss programme including breakfast cereals results in greater loss of body mass than a standardised web-based programme in a randomised controlled trial. *Obes Facts*, *7*(6), 361-375. https://doi.org/10.1159/000369193

Astbury, N. M., Aveyard, P., Nickless, A., Hood, K., Corfield, K., Lowe, R., & Jebb, S. A. (2018). Doctor Referral of Overweight People to Low Energy total diet replacement Treatment (DROPLET): pragmatic randomised controlled trial. *Bmj*, *362*, k3760. https://doi.org/10.1136/bmj.k3760

Axley, P., Kodali, S., Kuo, Y. F., Ravi, S., Seay, T., Parikh, N. M., & Singal, A. K. (2018). Text messaging approach improves weight loss in patients with nonalcoholic fatty liver disease: A randomized study. *Liver Int*, *38*(5), 924-931. https://doi.org/10.1111/liv.13622

Axley, P. D., Kodali, S., Ravi, S., Seay, T., Parikh, N., & Singal, A. K. (2017). Text messaging approach improves weight loss and ALT levels in patients with NAFLD [Conference Abstract]. *Gastroenterology*, *152*(5), S1055. http://www.embase.com/search/results?subaction=viewrecord&from=export&id=L618671667

Azar, K. M. J., Aurora, M., Wang, E. J., Muzaffar, A., Pressman, A., & Palaniappan, L. P. (2015). Virtual small groups for weight management: an innovative delivery mechanism for evidence-based lifestyle interventions among obese men [Article]. *Transl Behav Med*, *5*(1), 37-44. https://doi.org/10.1007/s13142-014-0296-6

Bachman, J. L., & Raynor, H. A. (2012). Effects of manipulating eating frequency during a behavioral weight loss intervention: a pilot randomized controlled trial. *Obesity (Silver Spring)*, *20*(5), 985-992. https://doi.org/10.1038/oby.2011.360

Bala, N., Price, S. N., Horan, C. M., Gerber, M. W., & Taveras, E. M. (2019). Use of Telehealth to Enhance Care in a Family-Centered Childhood Obesity Intervention. *Clin Pediatr (Phila)*, *58*(7), 789-797. https://doi.org/10.1177/0009922819837371

Banna, J., Campos, M., Gibby, C., Graulau, R. E., Meléndez, M., Reyes, A., Lee, J. E., & Palacios, C. (2017). Multi-site trial using short mobile messages (SMS) to improve infant weight in low-income minorities: Development, implementation, lessons learned and future applications. *Contemp Clin Trials*, *62*, 56-60. https://doi.org/10.1016/j.cct.2017.08.011

Barnason, S., Zimmerman, L., Schulz, P., Pullen, C., & Schuelke, S. (2019). Weight management telehealth intervention for overweight and obese rural cardiac rehabilitation participants: A randomised trial [Article]. *J Clin Nurs*, *28*(9-10), 1808-1818. https://doi.org/10.1111/jocn.14784

Bartholomew, L. M., Soules, K., Church, K., Shaha, S., Burlingame, J., Graham, G., Sauvage, L., & Zalud, I. (2015). Managing diabetes in pregnancy using cell phone/internet technology [Article]. *Clinical Diabetes*, *33*(4), 169-174. https://doi.org/10.2337/diaclin.33.4.169

Batch, B. C., Tyson, C., Bagwell, J., Corsino, L., Intille, S., Lin, P. H., Lazenka, T., Bennett, G., Bosworth, H. B., Voils, C., Grambow, S., Sutton, A., Bordogna, R., Pangborn, M., Schwager, J., Pilewski, K., Caccia, C., Burroughs, J., & Svetkey, L. P. (2014). Weight loss intervention for young adults using mobile technology: Design and rationale of a randomized controlled trial - Cell Phone Intervention for You (CITY) [Article]. *Contemp Clin Trials*, *37*(2), 333-341. https://doi.org/10.1016/j.cct.2014.01.003

Batra, P., Das, S. K., Salinardi, T., Robinson, L., Saltzman, E., Scott, T., Pittas, A. G., & Roberts, S. B. (2013). Eating behaviors as predictors of weight loss in a 6 month weight loss intervention. *Obesity (Silver Spring)*, *21*(11), 2256-2263. https://doi.org/10.1002/oby.20404

Beasley, J. M., Kirshner, L., Wylie-Rosett, J., Sevick, M. A., Deluca, L., & Chodosh, J. (2019). BRInging the Diabetes prevention program to GEriatric populations (BRIDGE): A feasibility study [Article]. *Pilot and Feasibility Studies*, *5*(1). https://doi.org/10.1186/s40814-019-0513-7

Becofsky, K., Wing, E. J., McCaffery, J., Boudreau, M., & Wing, R. R. (2017). A Randomized Controlled Trial of a Behavioral Weight Loss Program for Human Immunodeficiency Virus-Infected Patients. *Clin Infect Dis*, *65*(1), 154-157. https://doi.org/10.1093/cid/cix238

Bennett, W. L., Gudzune, K. A., Appel, L. J., & Clark, J. M. (2014). Insights from the POWER practice-based weight loss trial: a focus group study on the PCP's role in weight management. *J Gen Intern Med*, *29*(1), 50-58. https://doi.org/10.1007/s11606-013-2562-6

Berli, C., Stadler, G., Shrout, P. E., Bolger, N., & Scholz, U. (2018). Mediators of Physical Activity Adherence: Results from an Action Control Intervention in Couples. *Ann Behav Med*, *52*(1), 65-76. https://doi.org/10.1007/s12160-017-9923-z

Blomfield, R. L., Collins, C. E., Hutchesson, M. J., Young, M. D., Jensen, M. E., Callister, R., & Morgan, P. J. (2014). Impact of self-help weight loss resources with or without online support on the dietary intake of overweight and obese men: the SHED-IT randomised controlled trial. *Obes Res Clin Pract*, *8*(5), e476-487. https://doi.org/10.1016/j.orcp.2013.09.004

Boh, B., Lemmens, L. H. J. M., Jansen, A., Nederkoorn, C., Kerkhofs, V., Spanakis, G., Weiss, G., & Roefs, A. (2016). An Ecological Momentary Intervention for weight loss and healthy eating via smartphone and Internet: Study protocol for a randomised controlled trial [Article]. *Trials*, *17*(1). https://doi.org/10.1186/s13063-016-1280-x

Bollyky, J. B., Bravata, D., Yang, J., Williamson, M., & Schneider, J. (2018). Remote Lifestyle Coaching Plus a Connected Glucose Meter with Certified Diabetes Educator Support Improves Glucose and Weight Loss for People with Type 2 Diabetes. *J Diabetes Res*, *2018*, 3961730. https://doi.org/10.1155/2018/3961730

Bond, D. S., O'Leary, K. C., Thomas, J. G., Lipton, R. B., Papandonatos, G. D., Roth, J., Rathier, L., Daniello, R., & Wing, R. R. (2013). Can weight loss improve migraine headaches in obese women? Rationale and design of the Women's Health and Migraine (WHAM) randomized controlled trial [Article]. *Contemp Clin Trials*, *35*(1), 133-144. https://doi.org/10.1016/j.cct.2013.03.004

Bond, D. S., Thomas, J. G., Lipton, R. B., Pavlovic, J. M., O'Leary, K. C., Roth, J., Rathier, L., Evans, E. W., & Wing, R. R. (2017). The women's health and migraine trial (WHAM): A randomized controlled trial of behavioral weight loss as a treatment for migraine in women with overweight/obesity [Conference Abstract]. *Cephalalgia*, *37*(1), 163-164. https://doi.org/10.1177/0333102417719573

Bond, D. S., Thomas, J. G., Lipton, R. B., Roth, J., Pavlovic, J. M., Rathier, L., O'Leary, K. C., Evans, E. W., & Wing, R. R. (2018). Behavioral Weight Loss Intervention for Migraine: A Randomized Controlled Trial [Article]. *Obesity*, *26*(1), 81-87. https://doi.org/10.1002/oby.22069

Bosworth, H. B., Olsen, M. K., McCant, F., Stechuchak, K. M., Danus, S., Crowley, M. J., Goldstein, K. M., Zullig, L. L., & Oddone, E. Z. (2018). Telemedicine cardiovascular risk reduction in veterans: The CITIES trial [Article]. *American Heart Journal*, *199*, 122-129. https://doi.org/10.1016/j.ahj.2018.02.002

Braden, A., Flatt, S. W., Boutelle, K. N., Strong, D., Sherwood, N. E., & Rock, C. L. (2016). Emotional eating is associated with weight loss success among adults enrolled in a weight loss program. *J Behav Med*, *39*(4), 727-732. https://doi.org/10.1007/s10865-016-9728-8

Brantley, P. J., Stewart, D. W., Myers, V. H., Matthews-Ewald, M. R., Ard, J. D., Coughlin, J. W., Jerome, G. J., Samuel-Hodge, C., Lien, L. F., Gullion, C. M., Hollis, J. F., Svetkey, L. P., & Stevens, V. J. (2014). Psychosocial predictors of weight regain in the weight loss maintenance trial. *J Behav Med*, *37*(6), 1155-1168. https://doi.org/10.1007/s10865-014-9565-6

Breyer, B. N., Creasman, J. M., Richter, H. E., Myers, D., Burgio, K. L., Wing, R. R., West, D. S., Kusek, J. W., & Subak, L. L. (2018). A Behavioral Weight Loss Program and Nonurinary Incontinence Lower Urinary Tract Symptoms in Overweight and Obese Women with Urinary Incontinence: A Secondary Data Analysis of PRIDE. *J Urol*, *199*(1), 215-222. https://doi.org/10.1016/j.juro.2017.07.087

Brown, C., Goetz, J., Hamera, E., & Gajewski, B. (2014). Treatment response to the RENEW weight loss intervention in schizophrenia: impact of intervention setting. *Schizophr Res*, *159*(2-3), 421-425. https://doi.org/10.1016/j.schres.2014.09.018

Bui, Q. T. H., Pham, K. X., Tran, T. H., Le, L. T. T., & Nguyen, H. N. (2019). Impact of a pharmacist-led educational intervention on quality of life among patients with asthma [Article]. *Asian Journal of Pharmaceutical and Clinical Research*, *12*(6), 307-312. https://doi.org/10.22159/ajpcr.2019.v12i6.33705

Burke, L. E., Sereika, S. M., Parmanto, B., Beatrice, B., Cajita, M., Loar, I., Pulantara, I. W., Wang, Y., Kariuki, J., Yu, Y., Cedillo, M., Cheng, J., & Conroy, M. B. (2020). The SMARTER Trial: Design of a trial testing tailored mHealth feedback to impact self-monitoring of diet, physical activity, and weight [Article]. *Contemp Clin Trials*, *91*. https://doi.org/10.1016/j.cct.2020.105958

Burke, L. E., Styn, M. A., Sereika, S. M., Conroy, M. B., Ye, L., Glanz, K., Sevick, M. A., & Ewing, L. J. (2012). Using mHealth technology to enhance self-monitoring for weight loss: A randomized trial [Article]. *Am J Prev Med*, *43*(1), 20-26. https://doi.org/10.1016/j.amepre.2012.03.016

Burmeister, J. M., Hinman, N., Koball, A., Hoffmann, D. A., & Carels, R. A. (2013). Food addiction in adults seeking weight loss treatment. Implications for psychosocial health and weight loss. *Appetite*, *60*(1), 103-110. https://doi.org/10.1016/j.appet.2012.09.013

Byrne, J. L. S., Cameron Wild, T., Maximova, K., Browne, N. E., Holt, N. L., Cave, A. J., Martz, P., Ellendt, C., & Ball, G. D. C. (2018). A brief eHealth tool delivered in primary care to help parents prevent childhood obesity: a randomized controlled trial [Article]. *Pediatr Obes*, *13*(11), 659-667. https://doi.org/10.1111/ijpo.12200

Cadmus-Bertram, L. A., Marcus, B. H., Patterson, R. E., Parker, B. A., & Morey, B. L. (2015). Randomized Trial of a Fitbit-Based Physical Activity Intervention for Women [Article]. *Am J Prev Med*, *49*(3), 414-418. https://doi.org/10.1016/j.amepre.2015.01.020

Carels, R. A., Burmeister, J. M., Koball, A. M., Oehlhof, M. W., Hinman, N., LeRoy, M., Bannon, E., Ashrafioun, L., Storfer-Isser, A., Darby, L. A., & Gumble, A. (2014). A randomized trial comparing two approaches to weight loss: differences in weight loss maintenance. *J Health Psychol*, *19*(2), 296-311. https://doi.org/10.1177/1359105312470156

Carson, T. L., Jackson, B. E., Nolan, T. S., Williams, A., & Baskin, M. L. (2017). Lower depression scores associated with greater weight loss among rural black women in a behavioral weight loss program. *Transl Behav Med*, *7*(2), 320-329. https://doi.org/10.1007/s13142-016-0452-2

Carter, M. C., Burley, V. J., & Cade, J. E. (2015). Frequency of electronic dietary self monitoring using a smartphone app and weight loss [Conference Abstract]. *Proceedings of the Nutrition Society*, *74*(OCE4). https://doi.org/10.1017/S0029665115003195

Cassin, S. E., Sockalingam, S., Du, C., Wnuk, S., Hawa, R., & Parikh, S. V. (2016). A pilot randomized controlled trial of telephone-based cognitive behavioural therapy for preoperative bariatric surgery patients. *Behav Res Ther*, *80*, 17-22. https://doi.org/10.1016/j.brat.2016.03.001

Castelnuovo, G., Manzoni, G. M., Cuzziol, P., Cesa, G. L., Corti, S., Tuzzi, C., Villa, V., Liuzzi, A., Petroni, M. L., & Molinari, E. (2011). TECNOB study: Ad interim results of a randomized controlled trial of a multidisciplinary telecare intervention for obese patients with type-2 diabetes [Article]. *Clinical Practice and Epidemiology in Mental Health*, *7*, 44-50. https://doi.org/10.2174/1745017901107010044

Castelnuovo, G., Manzoni, G. M., Cuzziol, P., Cesa, G. L., Tuzzi, C., Villa, V., Liuzzi, A., Petroni, M. L., & Molinari, E. (2010). TECNOB: study design of a randomized controlled trial of a multidisciplinary telecare intervention for obese patients with type-2 diabetes. *BMC Public Health*, *10*, 204.

Castelnuovo, G., Manzoni, G. M., Villa, V., Cesa, G. L., Pietrabissa, G., & Molinari, E. (2011). The STRATOB study: design of a randomized controlled clinical trial of Cognitive Behavioral Therapy and Brief Strategic Therapy with telecare in patients with obesity and binge-eating disorder referred to residential nutritional rehabilitation. *Trials [Electronic Resource]*, *12*, 114.

Cato, K., Hyun, S., & Bakken, S. (2014). Response to a mobile health decision-support system for screening and management of Tobacco use [Article]. *Oncology Nursing Forum*, *41*(2), 145-152. https://doi.org/10.1188/14.ONF.145-152

Cattivelli, R., Castelnuovo, G., Musetti, A., Varallo, G., Spatola, C. A. M., Riboni, F. V., Usubini, A. G., Tosolin, F., Manzoni, G. M., Capodaglio, P., Rossi, A., Pietrabissa, G., & Molinari, E. (2018). ACTonHEALTH study protocol: Promoting psychological flexibility with activity tracker and mHealth tools to foster healthful lifestyle for obesity and other chronic health conditions [Article]. *Trials*, *19*(1). https://doi.org/10.1186/s13063-018-2968-x

Chai, L., Collins, C., May, C., Holder, C., Brown, L. J., & Burrows, T. (2019). An online telehealth nutrition intervention to support parents in child weight management - A randomised feasibility controlled trial [Conference Abstract]. *Obes Facts*, *12*, 111-112. https://doi.org/10.1159/000489691

Chan, R., Nguyen, M., Smith, R., Spencer, S., & Pit, S. W. (2019). Effect of Serial Anthropometric Measurements and Motivational Text Messages on Weight Reduction Among Workers: Pilot Randomized Controlled Trial. *JMIR Mhealth Uhealth*, *7*(4), e11832. https://doi.org/10.2196/11832

Chang, Y., Liu, S., & Hsu, Y. (2018). Efficacy of a game-based mobile application intervention in physical activity promotion and health management [Conference Abstract]. *Obes Facts*, *11*, 326-327. https://doi.org/10.1159/000489691

Chen, J. L., Guedes, C. M., & Lung, A. E. (2019). Smartphone-based Healthy Weight Management Intervention for Chinese American Adolescents: Short-term Efficacy and Factors Associated With Decreased Weight [Article]. *Journal of Adolescent Health*, *64*(4), 443-449. https://doi.org/10.1016/j.jadohealth.2018.08.022

Cherasard, P. D., Brathwaite, C. E., Barkan, A., Hall, K., Brathwaite, B. M., Fazzari, M., Brand, D. A., Djokic, B. B., Granoff, M., Ritter, J., Schaefer, M., Familusi, O., Norowski, K., & Dessart, G. (2018). Mobile Health Interventions are Ineffective in the Bariatric Surgery Population [Conference Abstract]. *Surgery for Obesity and Related Diseases*, *14*(11), S68. https://doi.org/10.1016/j.soard.2018.09.107

Cheung, N. W., Blumenthal, C., Smith, B. J., Hogan, R., Thiagalingam, A., Redfern, J., Barry, T., Cinnadaio, N., & Chow, C. K. (2019). A pilot randomised controlled trial of a text messaging intervention with customisation using linked data from wireless wearable activity monitors to improve risk factors following gestational diabetes [Article]. *Nutrients*, *11*(3). https://doi.org/10.3390/nu11030590

Choi, M. D., Standridge, A., & Landis-Piwowar, K. (2018). Improving cardiac function and body composition through incentive-based smartphone application in sedentary overweight adults [Conference Abstract]. *FASEB Journal*, *32*(1). http://www.embase.com/search/results?subaction=viewrecord&from=export&id=L622547102

Chow, L. S., Manoogian, E., Alvear, A. C., Wang, Q., Panda, S., & Mashek, D. G. (2019). Time restricted eating (TRE) promotes weight loss, alters body composition, and improves metabolic parameters in overweight humans [Conference Abstract]. *Diabetes*, *68*. https://doi.org/10.2337/db19-2076-P

Clark, D. O., Srinivas, P., Bodke, K., Keith, N., Hood, S., & Tu, W. (2018). Addressing people and place microenvironments in weight loss disparities (APP-Me): Design of a randomized controlled trial testing timely messages for weight loss behavior in low income Black and White Women. *Contemp Clin Trials*, *67*, 74-80. https://doi.org/10.1016/j.cct.2018.01.006

Cleo, G., Glasziou, P., Beller, E., Isenring, E., & Thomas, R. (2019). Habit-based interventions for weight loss maintenance in adults with overweight and obesity: a randomized controlled trial. *Int J Obes (Lond)*, *43*(2), 374-383. https://doi.org/10.1038/s41366-018-0067-4

Cleo, G., Hersch, J., & Thomas, R. (2018). Participant experiences of two successful habit-based weight-loss interventions in Australia: a qualitative study. *BMJ Open*, *8*(5), e020146. https://doi.org/10.1136/bmjopen-2017-020146

Collins, C. E., Jensen, M. E., Young, M. D., Callister, R., Plotnikoff, R. C., & Morgan, P. J. (2013). Improvement in erectile function following weight loss in obese men: the SHED-IT randomized controlled trial. *Obes Res Clin Pract*, *7*(6), e450-454. https://doi.org/10.1016/j.orcp.2013.07.004

Coughlin, J. W., Brantley, P. J., Champagne, C. M., Vollmer, W. M., Stevens, V. J., Funk, K., Dalcin, A. T., Jerome, G. J., Myers, V. H., Tyson, C., Batch, B. C., Charleston, J., Loria, C. M., Bauck, A., Hollis, J. F., Svetkey, L. P., & Appel, L. J. (2016). The impact of continued intervention on weight: Five-year results from the weight loss maintenance trial. *Obesity (Silver Spring)*, *24*(5), 1046-1053. https://doi.org/10.1002/oby.21454

Craigie, A. M., Macleod, M., Barton, K. L., Treweek, S., & Anderson, A. S. (2011). Supporting postpartum weight loss in women living in deprived communities: design implications for a randomised control trial. *Eur J Clin Nutr*, *65*(8), 952-958. https://doi.org/10.1038/ejcn.2011.56

Crain, A. L., Sherwood, N. E., Martinson, B. C., & Jeffery, R. W. (2018). Mediators of Weight Loss Maintenance in the Keep It Off Trial. *Ann Behav Med*, *52*(1), 9-18. https://doi.org/10.1007/s12160-017-9917-x

Crane, M. M., Lutes, L. D., Ward, D. S., Bowling, J. M., & Tate, D. F. (2015). A randomized trial testing the efficacy of a novel approach to weight loss among men with overweight and obesity. *Obesity (Silver Spring)*, *23*(12), 2398-2405. https://doi.org/10.1002/oby.21265

Creasy, S. A., Lang, W., Tate, D. F., Davis, K. K., & Jakicic, J. M. (2018). Pattern of Daily Steps is Associated with Weight Loss: Secondary Analysis from the Step-Up Randomized Trial. *Obesity (Silver Spring)*, *26*(6), 977-984. https://doi.org/10.1002/oby.22171

Dalton, W. T., 3rd, Schetzina, K. E., McBee, M. T., Maphis, L., Fulton-Robinson, H., Ho, A. L., Tudiver, F., & Wu, T. (2013). Parent report of child's health-related quality of life after a primary-care-based weight management program. *Child Obes*, *9*(6), 501-508. https://doi.org/10.1089/chi.2013.0036

Davis, A. M., Beaver, G., Dreyer Gillette, M., Nelson, E. L., Fleming, K., Swinburne Romine, R., Sullivan, D. K., Lee, R., Pettee Gabriel, K., Dean, K., Murray, M., & Faith, M. (2019). iAmHealthy: Rationale, design and application of a family-based mHealth pediatric obesity intervention for rural children [Article]. *Contemp Clin Trials*, *78*, 20-26. https://doi.org/10.1016/j.cct.2019.01.001

Davis, A. M., James, R. L., Boles, R. E., Goetz, J. R., Belmont, J., & Malone, B. (2011). The use of TeleMedicine in the treatment of paediatric obesity: feasibility and acceptability *Matern Child Nutr*, *7*(1), 71-79.

Davis, A. M., Sampilo, M., Gallagher, K. S., Dean, K., Saroja, M. B., Yu, Q., He, J., & Sporn, N. (2016). Treating rural paediatric obesity through telemedicine vs. telephone: Outcomes from a cluster randomized controlled trial. *J Telemed Telecare*, *22*(2), 86-95. https://doi.org/10.1177/1357633x15586642

Davis, A. M., Sampilo, M., Gallagher, K. S., Landrum, Y., & Malone, B. (2013). Treating rural pediatric obesity through telemedicine: outcomes from a small randomized controlled trial. *J Pediatr Psychol*, *38*(9), 932-943.

De Niet, J., Timman, R., Bauer, S., Van Den Akker, E., Buijks, H., De Klerk, C., Kordy, H., & Passchier, J. (2012). The effect of a short message service maintenance treatment on body mass index and psychological well-being in overweight and obese children: A randomized controlled trial [Article]. *Pediatr Obes*, *7*(3), 205-219. https://doi.org/10.1111/j.2047-6310.2012.00048.x

de Niet, J., Timman, R., Bauer, S., van den Akker, E., de Klerk, C., Kordy, H., & Passchier, J. (2012). Short message service reduces dropout in childhood obesity treatment: a randomized controlled trial. *Health Psychol*, *31*(6), 797-805. https://doi.org/10.1037/a0027498

de Vos, B. C., Runhaar, J., & Bierma-Zeinstra, S. M. (2014). Effectiveness of a tailor-made weight loss intervention in primary care. *Eur J Nutr*, *53*(1), 95-104. https://doi.org/10.1007/s00394-013-0505-y

Delisle, C., Sandin, S., Forsum, E., Henriksson, H., Trolle-Lagerros, Y., Larsson, C., Maddison, R., Ortega, F. B., Ruiz, J. R., Silfvernagel, K., Timpka, T., & Löf, M. (2015). A web- and mobile phone-based intervention to prevent obesity in 4-year-olds (MINISTOP): a population-based randomized controlled trial [Article]. *BMC Public Health*, *15*, 95. https://doi.org/10.1186/s12889-015-1444-8

Delisle Nyström, C., Sandin, S., Henriksson, P., Henriksson, H., Maddison, R., & Löf, M. (2018). A 12-month follow-up of a mobile-based (mHealth) obesity prevention intervention in pre-school children: the MINISTOP randomized controlled trial [Article]. *BMC Public Health*, *18*(1), 658. https://doi.org/10.1186/s12889-018-5569-4

Dennison, L., Morrison, L., Lloyd, S., Phillips, D., Stuart, B., Williams, S., Bradbury, K., Roderick, P., Murray, E., Michie, S., Little, P., & Yardley, L. (2014). Does brief telephone support improve engagement with a web-based weight management intervention? Randomized controlled trial. *J Med Internet Res*, *16*(3), e95.

Desouza, C. V., Padala, P. R., Haynatzki, G., Anzures, P., Demasi, C., & Shivaswamy, V. (2012). Role of apathy in the effectiveness of weight management programmes. *Diabetes Obes Metab*, *14*(5), 419-423. https://doi.org/10.1111/j.1463-1326.2011.01544.x

Dodd, J. M., Louise, J., Cramp, C., Grivell, R. M., Moran, L. J., & Deussen, A. R. (2018). Evaluation of a smartphone nutrition and physical activity application to provide lifestyle advice to pregnant women: The SNAPP randomised trial [Article]. *Maternal and Child Nutrition*, *14*(1). https://doi.org/10.1111/mcn.12502

Donnelly, J. E., Goetz, J., Gibson, C., Sullivan, D. K., Lee, R., Smith, B. K., Lambourne, K., Mayo, M. S., Hunt, S., Lee, J. H., Honas, J. J., & Washburn, R. A. (2013). Equivalent weight loss for weight management programs delivered by phone and clinic. *Obesity (Silver Spring)*, *21*(10), 1951-1959. https://doi.org/10.1002/oby.20334

Donnelly, J. E., Ptomey, L. T., Goetz, J. R., Sullivan, D. K., Gibson, C. A., Greene, J. L., Lee, R. H., Mayo, M. S., Honas, J. J., & Washburn, R. A. (2016). Weight management for adolescents with intellectual and developmental disabilities: Rationale and design for an 18 month randomized trial [Article]. *Contemp Clin Trials*, *51*, 88-95. https://doi.org/10.1016/j.cct.2016.10.009

Dreyer Gillette, M. L., Odar Stough, C., Best, C. M., Beck, A. R., & Hampl, S. E. (2014). Comparison of a condensed 12-week version and a 24-week version of a family-based pediatric weight management program. *Child Obes*, *10*(5), 375-382. https://doi.org/10.1089/chi.2014.0037

Duncan, M. J., Brown, W. J., Burrows, T. L., Collins, C. E., Fenton, S., Glozier, N., Kolt, G. S., Morgan, P. J., Hensley, M., Holliday, E. G., Murawski, B., Plotnikoff, R. C., Rayward, A. T., Stamatakis, E., & Vandelanotte, C. (2018). Examining the efficacy of a multicomponent m-Health physical activity, diet and sleep intervention for weight loss in overweight and obese adults: Randomised controlled trial protocol [Article]. *BMJ Open*, *8*(10). https://doi.org/10.1136/bmjopen-2018-026179

Dutton, G. R., Fontaine, K. R., Alcorn, A. S., Dawson, J., Capers, P. L., & Allison, D. B. (2015). Randomized controlled trial examining expectancy effects on the accuracy of weight measurement. *Clin Obes*, *5*(1), 38-41. https://doi.org/10.1111/cob.12083

Dutton, G. R., Nackers, L. M., Dubyak, P. J., Rushing, N. C., Huynh, T. V., Tan, F., Anton, S. D., & Perri, M. G. (2014). A randomized trial comparing weight loss treatment delivered in large versus small groups. *Int J Behav Nutr Phys Act*, *11*, 123. https://doi.org/10.1186/s12966-014-0123-y

Eakin, E. G., Reeves, M. M., Marshall, A. L., Dunstan, D. W., Graves, N., Healy, G. N., Bleier, J., Barnett, A. G., O'Moore-Sullivan, T., Russell, A., & Wilkie, K. (2010). Living Well with Diabetes: a randomized controlled trial of a telephone-delivered intervention for maintenance of weight loss, physical activity and glycaemic control in adults with type 2 diabetes. *BMC Public Health*, *10*, 452. https://doi.org/10.1186/1471-2458-10-452

Eakin, E. G., Winkler, E. A., Dunstan, D. W., Healy, G. N., Owen, N., Marshall, A. M., Graves, N., & Reeves, M. M. (2014). Living well with diabetes: 24-month outcomes from a randomized trial of telephone-delivered weight loss and physical activity intervention to improve glycemic control [Article]. *Diabetes Care*, *37*(8), 2177-2185. https://doi.org/10.2337/dc13-2427

Edney, S., Ryan, J. C., Olds, T., Monroe, C., Fraysse, F., Vandelanotte, C., Plotnikoff, R., Curtis, R., & Maher, C. (2019). User Engagement and Attrition in an App-Based Physical Activity Intervention: Secondary Analysis of a Randomized Controlled Trial [Article]. *J Med Internet Res*, *21*(11), e14645. https://doi.org/10.2196/14645

Eight-year weight losses with an intensive lifestyle intervention: the look AHEAD study. (2014). *Obesity (Silver Spring)*, *22*(1), 5-13. https://doi.org/10.1002/oby.20662

Ek, A., Alexandrou, C., Delisle Nyström, C., Direito, A., Eriksson, U., Hammar, U., Henriksson, P., Maddison, R., Trolle Lagerros, Y., & Löf, M. (2018). The Smart City Active Mobile Phone Intervention (SCAMPI) study to promote physical activity through active transportation in healthy adults: a study protocol for a randomised controlled trial [Article]. *BMC Public Health*, *18*(1), 880. https://doi.org/10.1186/s12889-018-5658-4

Ekambareshwar, M., Mihrshahi, S., Wen, L. M., Taki, S., Bennett, G., Baur, L. A., & Rissel, C. (2018). Facilitators and challenges in recruiting pregnant women to an infant obesity prevention programme delivered via telephone calls or text messages. *Trials*, *19*(1), 494. https://doi.org/10.1186/s13063-018-2871-5

Evans, E. H., Araujo-Soares, V., Adamson, A., Batterham, A. M., Brown, H., Campbell, M., Dombrowski, S. U., Guest, A., Jackson, D., Kwasnicka, D., Ladha, K., McColl, E., Olivier, P., Rothman, A. J., Sainsbury, K., Steel, A. J., Steen, I. N., Vale, L., White, M., Wright, P., & Sniehotta, F. F. (2015). The NULevel trial of a scalable, technology-assisted weight loss maintenance intervention for obese adults after clinically significant weight loss: study protocol for a randomised controlled trial. *Trials [Electronic Resource]*, *16*, 421.

Evans, W. E., Raynor, H. A., Howie, W., Lipton, R. B., Thomas, G. J., Wing, R. R., Pavlovic, J., Farris, S. G., & Bond, D. S. (2019). Associations between lifestyle intervention-related changes in dietary targets and migraine headaches among women in the Women's Health and Migraine (WHAM) randomized controlled trial [Article in Press]. *Obesity Science and Practice*. https://doi.org/10.1002/osp4.376

Faghri, P. D., Simon, J., Huedo-Medina, T., & Gorin, A. (2017). Perceived Self-Efficacy and Financial Incentives: Factors Affecting Health Behaviors and Weight Loss in a Workplace Weight Loss Intervention. *J Occup Environ Med*, *59*(5), 453-460. https://doi.org/10.1097/jom.0000000000000987

Fanning, J., Opina, M. T., Leng, I., Lyles, M. F., Nicklas, B. J., & Rejeski, W. J. (2018). Empowered with Movement to Prevent Obesity & Weight Regain (EMPOWER): Design and methods. *Contemp Clin Trials*, *72*, 35-42.

Faruqui, S. H. A., Du, Y., Meka, R., Alaeddini, A., Li, C., Shirinkam, S., & Wang, J. (2019). Development of a Deep Learning Model for Dynamic Forecasting of Blood Glucose Level for Type 2 Diabetes Mellitus: Secondary Analysis of a Randomized Controlled Trial [Article]. *JMIR Mhealth Uhealth*, *7*(11), e14452. https://doi.org/10.2196/14452

Fazzino, T. L., Fabian, C., & Befort, C. A. (2017). Change in Physical Activity During a Weight Management Intervention for Breast Cancer Survivors: Association with Weight Outcomes [Randomized Controlled Trial]. *Obesity*, *25 Suppl 2*, S109-S115.

Fernandez-Luque, L., Singh, M., Ofli, F., Mejova, Y. A., Weber, I., Aupetit, M., Jreige, S. K., Elmagarmid, A., Srivastava, J., & Ahmedna, M. (2017). Implementing 360° Quantified Self for childhood obesity: feasibility study and experiences from a weight loss camp in Qatar [Article]. *BMC Med Inform Decis Mak*, *17*(1), 37. https://doi.org/10.1186/s12911-017-0432-6

Ferrante, J. M., Devine, K. A., Bator, A., Rodgers, A., Ohman-Strickland, P. A., Bandera, E. V., & Hwang, K. O. (2018). Feasibility and potential efficacy of commercial mHealth/eHealth tools for weight loss in African American breast cancer survivors: pilot randomized controlled trial [Article in Press]. *Transl Behav Med*. https://doi.org/10.1093/tbm/iby124

Fiks, A. G., Gruver, R. S., Bishop-Gilyard, C. T., Shults, J., Virudachalam, S., Suh, A. W., Gerdes, M., Kalra, G. K., DeRusso, P. A., Lieberman, A., Weng, D., Elovitz, M. A., Berkowitz, R. I., & Power, T. J. (2017). A Social Media Peer Group for Mothers To Prevent Obesity from Infancy: The Grow2Gether Randomized Trial [Article]. *Child Obes*, *13*(5), 356-368. https://doi.org/10.1089/chi.2017.0042

Finkelstein, J., Bedra, M., Li, X., Wood, J., & Ouyang, P. (2015). Mobile App to Reduce Inactivity in Sedentary Overweight Women. *Stud Health Technol Inform*, *216*, 89-92.

Fischer, H. H., Fischer, I. P., Pereira, R. I., Furniss, A. L., Rozwadowski, J. M., Moore, S. L., Durfee, M. J., Raghunath, S. G., Tsai, A. G., & Havranek, E. P. (2016). Text Message Support for Weight Loss in Patients With Prediabetes: A Randomized Clinical Trial. *Diabetes Care*, *39*(8), 1364-1370. https://doi.org/10.2337/dc15-2137

Fisher, W. A., Orsama, A. L., Lähteenmäki, J., Harno, K., Kulju, M., Wintergerst, E., Schachner, H., Stenger, P., Leppänen, J., Kaijanranta, H., Salaspuro, V., & Liukko, K. (2013). Remote patient reporting and automated mobile telephone feedback reduce HbA1c and weight in individuals with type 2 diabetes: Results of pilot research [Conference Abstract]. *Diabetes Technology and Therapeutics*, *15*, A8. https://doi.org/10.1089/dia.2012.1221

Fleischman, A., Hourigan, S. E., Lyon, H. N., Landry, M. G., Reynolds, J., Steltz, S. K., Robinson, L., Keating, S., Feldman, H. A., Antonelli, R. C., Ludwig, D. S., & Ebbeling, C. B. (2016). Creating an integrated care model for childhood obesity: a randomized pilot study utilizing telehealth in a community primary care setting [Article]. *Clin Obes*, *6*(6), 380-388. https://doi.org/10.1111/cob.12166

Fogarty, S., Stojanovska, L., Harris, D., Zaslawski, C., Mathai, M. L., & McAinch, A. J. (2015). A randomised cross-over pilot study investigating the use of acupuncture to promote weight loss and mental health in overweight and obese individuals participating in a weight loss program. *Eat Weight Disord*, *20*(3), 379-387. https://doi.org/10.1007/s40519-014-0175-7

Foley, P., Steinberg, D., Levine, E., Askew, S., Batch, B. C., Puleo, E. M., Svetkey, L. P., Bosworth, H. B., DeVries, A., Miranda, H., & Bennett, G. G. (2016). Track: A randomized controlled trial of a digital health obesity treatment intervention for medically vulnerable primary care patients. *Contemp Clin Trials*, *48*, 12-20.

Forman, E. M., Goldstein, S. P., Crochiere, R. J., Butryn, M. L., Juarascio, A. S., Zhang, F., & Foster, G. D. (2019). Randomized controlled trial of OnTrack, a just-in-time adaptive intervention designed to enhance weight loss [Article]. *Transl Behav Med*, *9*(6), 989-1001. https://doi.org/10.1093/tbm/ibz137

Forman, E. M., Shaw, J. A., Goldstein, S. P., Butryn, M. L., Martin, L. M., Meiran, N., Crosby, R. D., & Manasse, S. M. (2016). Mindful decision making and inhibitory control training as complementary means to decrease snack consumption [Article]. *Appetite*, *103*, 176-183. https://doi.org/10.1016/j.appet.2016.04.014

Fred Wen, C. K., Belcher, B. R., Chou, C. P., Weigensberg, M. J., Black, D. S., & Spruijt-Metz, D. (2019). The momentary affective states and diurnal cortisol rhythm in minority youth [Conference Abstract]. *Psychosom Med*, *81*(4), A123. https://doi.org/10.1097/PSY.0000000000000699

Frisch, S., Zittermann, A., Berthold, H. K., Götting, C., Kuhn, J., Kleesiek, K., Stehle, P., & Körtke, H. (2009). A randomized controlled trial on the efficacy of carbohydrate-reduced or fat-reduced diets in patients attending a telemedically guided weight loss program [Article]. *Cardiovasc Diabetol*, *8*. https://doi.org/10.1186/1475-2840-8-36

Fukuoka, Y., Gay, C., Joiner, K., & Vittinghoff, E. (2014). A novel mobile phone delivered diabetes prevention program in overweight adults at risk for type 2 diabetes-a randomized controlled trial [Conference Abstract]. *Circulation*, *130*. http://www.embase.com/search/results?subaction=viewrecord&from=export&id=L71710941

Fukuoka, Y., Vittinghoff, E., & Hooper, J. (2018). A weight loss intervention using a commercial mobile application in latino americans-adelgaza trial [Conference Abstract]. *Circulation*, *137*. http://www.embase.com/search/results?subaction=viewrecord&from=export&id=L621615224

Fuller, N. R., Carter, H., Schofield, D., Hauner, H., Jebb, S. A., Colagiuri, S., & Caterson, I. D. (2014). Cost effectiveness of primary care referral to a commercial provider for weight loss treatment, relative to standard care: a modelled lifetime analysis. *Int J Obes (Lond)*, *38*(8), 1104-1109. https://doi.org/10.1038/ijo.2013.227

Fuller, N. R., Colagiuri, S., Schofield, D., Olson, A. D., Shrestha, R., Holzapfel, C., Wolfenstetter, S. B., Holle, R., Ahern, A. L., Hauner, H., Jebb, S. A., & Caterson, I. D. (2013). A within-trial cost-effectiveness analysis of primary care referral to a commercial provider for weight loss treatment, relative to standard care--an international randomised controlled trial. *Int J Obes (Lond)*, *37*(6), 828-834. https://doi.org/10.1038/ijo.2012.139

Funk, L. M., Grubber, J. M., McVay, M. A., Olsen, M. K., Yancy, W. S., & Voils, C. I. (2018). Patient predictors of weight loss following a behavioral weight management intervention among US Veterans with severe obesity. *Eat Weight Disord*, *23*(5), 587-595. https://doi.org/10.1007/s40519-017-0425-6

Gallagher, K. S., Davis, A. M., Malone, B., Landrum, Y., & Black, W. (2011). Treating rural pediatric obesity through telemedicine: baseline data from a randomized controlled trial. *J Pediatr Psychol*, *36*(6), 687-695.

Garcia, D. O., Valdez, L. A., Bell, M. L., Humphrey, K., Hingle, M., McEwen, M., & Hooker, S. P. (2018). A gender- and culturally-sensitive weight loss intervention for Hispanic males: The ANIMO randomized controlled trial pilot study protocol and recruitment methods [Article]. *Contemporary Clinical Trials Communications*, *9*, 151-163. https://doi.org/10.1016/j.conctc.2018.01.010

Geiker, N. R., Ritz, C., Pedersen, S. D., Larsen, T. M., Hill, J. O., & Astrup, A. (2016). A weight-loss program adapted to the menstrual cycle increases weight loss in healthy, overweight, premenopausal women: a 6-mo randomized controlled trial. *Am J Clin Nutr*, *104*(1), 15-20.

Gillison, F., Stathi, A., Reddy, P., Perry, R., Taylor, G., Bennett, P., Dunbar, J., & Greaves, C. (2015). Processes of behavior change and weight loss in a theory-based weight loss intervention program: a test of the process model for lifestyle behavior change. *Int J Behav Nutr Phys Act*, *12*, 2. https://doi.org/10.1186/s12966-014-0160-6

Glynn, L. G., Hayes, P. S., Casey, M., Glynn, F., Alvarez-Iglesias, A., Newell, J., ÓLaighin, G., Heaney, D., & Murphy, A. W. (2013). SMART MOVE - a smartphone-based intervention to promote physical activity in primary care: Study protocol for a randomized controlled trial [Article]. *Trials*, *14*(1). https://doi.org/10.1186/1745-6215-14-157

Godino, J. G., Golaszewski, N. M., Norman, G. J., Rock, C. L., Griswold, W. G., Arredondo, E., Marshall, S., Kolodziejczyk, J., Dillon, L., Raab, F., Jain, S., Crawford, M., Merchant, G., & Patrick, K. (2019). Text messaging and brief phone calls for weight loss in overweight and obese English- and Spanish-speaking adults: A 1-year, parallel-group, randomized controlled trial. *PLoS Med*, *16*(9), e1002917. https://doi.org/10.1371/journal.pmed.1002917

Goldstein, S. P., Thomas, J. G., Foster, G. D., Turner-McGrievy, G., Butryn, M. L., Herbert, J. D., Martin, G. J., & Forman, E. M. (2020). Refining an algorithm-powered just-in-time adaptive weight control intervention: A randomized controlled trial evaluating model performance and behavioral outcomes [Article in Press]. *Health informatics journal*, 1460458220902330. https://doi.org/10.1177/1460458220902330

González, C., Herrero, P., Cubero, J. M., Iniesta, J. M., Hernando, M. E., García-Sáez, G., Serrano, A. J., Martinez-Sarriegui, I., Perez-Gandia, C., Gómez, E. J., Rubinat, E., Alcantara, V., Brugués, E., Chico, A., Mato, E., Bell, O., Corcoy, R., & de Leiva, A. (2013). PREDIRCAM eHealth platform for individualized telemedical assistance for lifestyle modification in the treatment of obesity, diabetes, and cardiometabolic risk prevention: a pilot study (PREDIRCAM 1). *J Diabetes Sci Technol*, *7*(4), 888-897. https://doi.org/10.1177/193229681300700411

Goode, A. D., Winkler, E. A., Reeves, M. M., & Eakin, E. G. (2015). Relationship between intervention dose and outcomes in living well with diabetes--a randomized trial of a telephone-delivered lifestyle-based weight loss intervention. *Am J Health Promot*, *30*(2), 120-129. https://doi.org/10.4278/ajhp.140206-QUAN-62

Gorin, A. A., Lenz, E. M., Cornelius, T., Huedo-Medina, T., Wojtanowski, A. C., & Foster, G. D. (2018). Randomized Controlled Trial Examining the Ripple Effect of a Nationally Available Weight Management Program on Untreated Spouses. *Obesity (Silver Spring)*, *26*(3), 499-504. https://doi.org/10.1002/oby.22098

Gorin, A. A., Powers, T. A., Koestner, R., Wing, R. R., & Raynor, H. A. (2014). Autonomy support, self-regulation, and weight loss. *Health Psychol*, *33*(4), 332-339. https://doi.org/10.1037/a0032586

Goswami, U., Black, A., Krohn, B., Meyers, W., & Iber, C. (2019). Smartphone-based delivery of oropharyngeal exercises for treatment of snoring: a randomized controlled trial [Article]. *Sleep and Breathing*, *23*(1), 243-250. https://doi.org/10.1007/s11325-018-1690-y

Goulis, D. G., Giaglis, G. D., Boren, S. A., Lekka, I., Bontis, E., Balas, E. A., Maglaveras, N., & Avramides, A. (2004). Effectiveness of home-centered care through telemedicine applications for overweight and obese patients: a randomized controlled trial [Clinical Trial

Randomized Controlled Trial]. *International Journal of Obesity & Related Metabolic Disorders: Journal of the International Association for the Study of Obesity*, *28*(11), 1391-1398.

Graham, J., Tudor, K., Jebb, S. A., Lewis, A., Tearne, S., Adab, P., Begh, R., Jolly, K., Daley, A., Farley, A., Lycett, D., Nickless, A., & Aveyard, P. (2019). The equity impact of brief opportunistic interventions to promote weight loss in primary care: secondary analysis of the BWeL randomised trial. *BMC Med*, *17*(1), 51. https://doi.org/10.1186/s12916-019-1284-y

Graham, M. L., Strawderman, M. S., Demment, M., & Olson, C. M. (2017). Does Usage of an eHealth Intervention Reduce the Risk of Excessive Gestational Weight Gain? Secondary Analysis From a Randomized Controlled Trial [Randomized Controlled Trial]. *J Med Internet Res*, *19*(1), e6.

Graham Thomas, J., & Bond, D. S. (2015). Behavioral response to a just-in-time adaptive intervention (JITAI) to reduce sedentary behavior in obese adults: Implications for JITAI optimization [Article]. *Health Psychology*, *34*, 1261-1267. https://doi.org/10.1037/hea0000304

Granado-Font, E., Flores-Mateo, G., Sorli-Aguilar, M., Montana-Carreras, X., Ferre-Grau, C., Barrera-Uriarte, M. L., Oriol-Colominas, E., Rey-Renones, C., Caules, I., Satue-Gracia, E. M., & Group, O. S. (2015). Effectiveness of a Smartphone application and wearable device for weight loss in overweight or obese primary care patients: protocol for a randomised controlled trial. *BMC Public Health*, *15*, 531.

Greene, E. M., Ainscough, K. M., Kennelly, M. A., O'Brien, E. C., McAuliffe, F. M., & Geraghty, A. (2018). Do pregnant women with overweight and obesity find a nutrition and exercise intervention with smartphone app support acceptable? Findings from the PEARs randomised control trial [Conference Abstract]. *BJOG: An International Journal of Obstetrics and Gynaecology*, *125*, 74-75. https://doi.org/10.1111/1471-0528.15191

Griauzde, D. H., Kullgren, J. T., Liestenfeltz, B., Richardson, C., & Heisler, M. (2018). A mobile phone-based program to promote healthy behaviors among adults with prediabetes: Study protocol for a pilot randomized controlled trial [Article]. *Pilot and Feasibility Studies*, *4*(1). https://doi.org/10.1186/s40814-018-0246-z

Guan, Y. Y., Chen, C., & Yoong, J. S. (2015). Using financial incentives to motivate stair use in a workplace setting: Results from a randomized controlled trial [Conference Abstract]. *Value in Health*, *18*(7), A568. http://www.embase.com/search/results?subaction=viewrecord&from=export&id=L72084136

Guerra, N., Neumeier, W. H., Breslin, L., Geer, B., Thirumalai, M., Ervin, D. A., & Rimmer, J. H. (2019). Feedback and Strategies From People With Intellectual Disability Completing a Personalized Online Weight Loss Intervention: A Qualitative Analysis [Article]. *Intellectual and developmental disabilities*, *57*(6), 527-544. https://doi.org/10.1352/1934-9556-57.6.527

Haapala, I., Barengo, N. C., Biggs, S., Surakka, L., & Manninen, P. (2009). Weight loss by mobile phone: a 1-year effectiveness study [Article]. *Public Health Nutr*, *12*(12), 2382-2391. http://www.embase.com/search/results?subaction=viewrecord&from=export&id=L355869494

Haddock, C. K., Poston, W. S., Lagrotte, C., Klotz, A. A., Oliver, T. L., Vander Veur, S. S., Foster, G. D., Jebb, S. A., Moore, C., Roberts, S. A., Reeves, R. S., Bolton, M. P., & Foreyt, J. P. (2014). Findings from an online behavioural weight management programme provided with or without a fortified diet beverage. *Br J Nutr*, *111*(2), 372-379. https://doi.org/10.1017/s0007114513002377

Hadžiabdić, M. O., Mucalo, I., Hrabač, P., Matić, T., Rahelić, D., & Božikov, V. (2015). Factors predictive of drop-out and weight loss success in weight management of obese patients. *J Hum Nutr Diet*, *28 Suppl 2*, 24-32. https://doi.org/10.1111/jhn.12270

Hageman, P. A., Pullen, C. H., Hertzog, M., Pozehl, B., Eisenhauer, C., & Boeckner, L. S. (2017). Web-based interventions alone or supplemented with peer-led support or professional email counseling for weight loss and weight maintenance in women from rural communities: results of a clinical trial [Article]. *J Obes*, *2017*. https://doi.org/10.1155/2017/1602627

Haggerty, A. F., Hagemann, A., Barnett, M., Thornquist, M., Neuhouser, M. L., Horowitz, N., Colditz, G. A., Sarwer, D. B., Ko, E. M., & Allison, K. C. (2017). A Randomized, Controlled, Multicenter Study of Technology-Based Weight Loss Interventions among Endometrial Cancer Survivors. *Obesity*, *25 Suppl 2*, S102-S108.

Haggerty, A. F., Huepenbecker, S., Sarwer, D. B., Spitzer, J., Raggio, G., Chu, C. S., Ko, E., & Allison, K. C. (2016). The use of novel technology-based weight loss interventions for obese women with endometrial hyperplasia and cancer. *Gynecol Oncol*, *140*(2), 239-244.

Haire-Joshu, D., Schwarz, C. D., Steger-May, K., Lapka, C., Schechtman, K., Brownson, R. C., & Tabak, R. G. (2018). A Randomized Trial of Weight Change in a National Home Visiting Program. *Am J Prev Med*, *54*(3), 341-351. https://doi.org/10.1016/j.amepre.2017.12.012

Hammersley, M. L., Jones, R. A., & Okely, A. D. (2017). Time2bHealthy – An online childhood obesity prevention program for preschool-aged children: A randomised controlled trial protocol [Article]. *Contemp Clin Trials*, *61*, 73-80. https://doi.org/10.1016/j.cct.2017.07.022

Hammersley, M. L., Okely, A. D., Batterham, M. J., & Jones, R. A. (2019). An Internet-Based Childhood Obesity Prevention Program (Time2bHealthy) for Parents of Preschool-Aged Children: Randomized Controlled Trial. *J Med Internet Res*, *21*(2), e11964. https://doi.org/10.2196/11964

Handjieva-Darlenska, T., Holst, C., Grau, K., Blaak, E., Martinez, J. A., Oppert, J. M., Taylor, M. A., Sørensen, T. I., & Astrup, A. (2012). Clinical correlates of weight loss and attrition during a 10-week dietary intervention study: results from the NUGENOB project. *Obes Facts*, *5*(6), 928-936. https://doi.org/10.1159/000345951

Hansel, B., Giral, P., Gambotti, L., Lafourcade, A., Peres, G., Filipecki, C., Kadouch, D., Hartemann, A., Oppert, J. M., Bruckert, E., Marre, M., Bruneel, A., Duchene, E., & Roussel, R. (2017). A Fully Automated Web-Based Program Improves Lifestyle Habits and HbA1c in Patients With Type 2 Diabetes and Abdominal Obesity: Randomized Trial of Patient E-Coaching Nutritional Support (The ANODE Study). *J Med Internet Res*, *19*(11), e360. https://doi.org/10.2196/jmir.7947

Harden, S. M., You, W., Almeida, F. A., Hill, J. L., Linnan, L. A., Allen, K. C., & Estabrooks, P. A. (2015). Does Successful Weight Loss in an Internet-Based Worksite Weight Loss Program Improve Employee Presenteeism and Absenteeism? *Health Educ Behav*, *42*(6), 769-774. https://doi.org/10.1177/1090198115578751

Harries, T., Eslambolchilar, P., Rettie, R., Stride, C., Walton, S., & van Woerden, H. C. (2016). Effectiveness of a smartphone app in increasing physical activity amongst male adults: a randomised controlled trial [Article]. *BMC Public Health*, *16*, 925. https://doi.org/10.1186/s12889-016-3593-9

Hawkes, A., Pakenham, K., Courneya, K., Peter, B., & Chambers, S. (2010). 'Canchange': A trial of a telephone-delivered lifestyle intervention for colorectal cancer (CRC) survivors [Conference Abstract]. *Asia-Pacific Journal of Clinical Oncology*, *6*, 193. https://doi.org/10.1111/j.1743-7563.2010.01349.x

Hayot, M., Ologeanu-Taddei, R., Bouaynaya, W., Ayoub, B., & Bughin, F. (2019). Assessment by Patients of a Connected System for Telerehabilitation: Lessons Learned from a Randomized Qualitative Study [Randomized Controlled Trial]. *Studies in Health Technology & Informatics*, *264*, 1931-1932.

Hebden, L., Balestracci, K., McGeechan, K., Denney-Wilson, E., Harris, M., Bauman, A., & Allman-Farinelli, M. (2013). 'TXT2BFiT' a mobile phone-based healthy lifestyle program for preventing unhealthy weight gain in young adults: Study protocol for a randomized controlled trial [Article]. *Trials*, *14*(1). https://doi.org/10.1186/1745-6215-14-75

Heldt, K., Büchter, D. J., Brogle, B., Shih, C. I., Rüegger, D., Filler, A., Gindrat, P., Durrer, D., Farpour-Lambert, N., & Kowatsch, T. (2018). Telemedicine Therapy for Overweight Adolescents: First Results of a Novel Smartphone App Intervention using a Behavioural Health Platform [Conference Abstract]. *Obes Facts*, *11*, 214-215. https://doi.org/10.1159/000489691

Helle, C., Hillesund, E. R., Omholt, M. L., & Øverby, N. C. (2017). Early food for future health: a randomized controlled trial evaluating the effect of an eHealth intervention aiming to promote healthy food habits from early childhood. *BMC Public Health*, *17*(1), 729. https://doi.org/10.1186/s12889-017-4731-8

Helle, C., Hillesund, E. R., Wills, A. K., & Øverby, N. C. (2019). Evaluation of an eHealth intervention aiming to promote healthy food habits from infancy -the Norwegian randomized controlled trial Early Food for Future Health [Article]. *International Journal of Behavioral Nutrition and Physical Activity*, *16*(1). https://doi.org/10.1186/s12966-018-0763-4

Henriksen, M., Christensen, R., Hunter, D. J., Gudbergsen, H., Boesen, M., Lohmander, L. S., & Bliddal, H. (2014). Structural changes in the knee during weight loss maintenance after a significant weight loss in obese patients with osteoarthritis: a report of secondary outcome analyses from a randomized controlled trial. *Osteoarthritis Cartilage*, *22*(5), 639-646. https://doi.org/10.1016/j.joca.2014.03.003

Hernández-Reyes, A., Cámara-Martos, F., Molina Recio, G., Molina-Luque, R., Romero-Saldaña, M., & Moreno Rojas, R. (2020). Push Notifications From a Mobile App to Improve the Body Composition of Overweight or Obese Women: Randomized Controlled Trial [Article]. *JMIR Mhealth Uhealth*, *8*(2), e13747. https://doi.org/10.2196/13747

Hernández-Reyes, A., Molina-Recio, G., Molina-Luque, R., Romero-Saldaña, M., Cámara-Martos, F., & Moreno-Rojas, R. (2020). Effectiveness of PUSH notifications from a mobile app for improving the body composition of overweight or obese women: a protocol of a three-armed randomized controlled trial [Article]. *BMC Med Inform Decis Mak*, *20*(1), 40. https://doi.org/10.1186/s12911-020-1058-7

Höchsmann, C., Müller, O., Ambühl, M., Klenk, C., Königstein, K., Infanger, D., Walz, S. P., & Schmidt-Trucksäss, A. (2019). Novel Smartphone Game Improves Physical Activity Behavior in Type 2 Diabetes [Article]. *Am J Prev Med*, *57*(1), 41-50. https://doi.org/10.1016/j.amepre.2019.02.017

Holland-Carter, L., Tuerk, P. W., Wadden, T. A., Fujioka, K. N., Becker, L. E., Miller-Kovach, K., Hollander, P. L., Garvey, W. T., Weiss, D., Rubino, D. M., Kushner, R. F., Malcolm, R. J., Raum, W. J., Hermayer, K. L., Veliko, J. L., Rost, S. L., Sora, N. D., Salyer, J. L., & O'Neil, P. M. (2017). Impact on psychosocial outcomes of a nationally available weight management program tailored for individuals with type 2 diabetes: Results of a randomized controlled trial. *Journal of Diabetes & its Complications*, *31*(5), 891-897.

Holmen, H., Torbjørnsen, A., & Ribu, L. (2015). Patient-reported outcomes and the use of a diabetes diary mobile application to attain lifestyle changes for persons with type 2 diabetes [Conference Abstract]. *Quality of Life Research*, *24*(1), 69. https://doi.org/10.1007/s11136-015-1078-4

Holmen, H., Wahl, A., Torbjørnsen, A., Jenum, A. K., Småstuen, M. C., & Ribu, L. (2016). Stages of change for physical activity and dietary habits in persons with type 2 diabetes included in a mobile health intervention: The Norwegian study in RENEWING HEALTH [Article]. *BMJ Open Diabetes Research and Care*, *4*(1). https://doi.org/10.1136/bmjdrc-2016-000193

Holzapfel, C., Cresswell, L., Ahern, A. L., Fuller, N. R., Eberhard, M., Stoll, J., Mander, A. P., Jebb, S. A., Caterson, I. D., & Hauner, H. (2014). The challenge of a 2-year follow-up after intervention for weight loss in primary care. *Int J Obes (Lond)*, *38*(6), 806-811. https://doi.org/10.1038/ijo.2013.180

Huang, J. S., Dillon, L., Terrones, L., Schubert, L., Roberts, W., Finklestein, J., Swartz, M. C., Norman, G. J., & Patrick, K. (2014). Fit4Life: A weight loss intervention for children who have survived childhood leukemia [Article]. *Pediatric Blood and Cancer*, *61*(5), 894-900. https://doi.org/10.1002/pbc.24937

Huber, J. M., Shapiro, J. S., Wieland, M. L., Croghan, I. T., Vickers Douglas, K. S., Schroeder, D. R., Hathaway, J. C., & Ebbert, J. O. (2015). Telecoaching plus a portion control plate for weight care management: a randomized trial. *Trials [Electronic Resource]*, *16*, 323.

Hult, M., Bonn, S. E., Andersson, E., Spetz, K., & Lagerros, Y. T. (2018). The PromMera study – an RCT evaluating the effect of a smartphone application to improve lifestyle after bariatric surgery [Conference Abstract]. *Surgery for Obesity and Related Diseases*, *14*(11), S160-S162. https://doi.org/10.1016/j.soard.2018.09.367

Huseinovic, E., Winkvist, A., Bertz, F., & Brekke, H. K. (2014). Changes in food choice during a successful weight loss trial in overweight and obese postpartum women. *Obesity (Silver Spring)*, *22*(12), 2517-2523. https://doi.org/10.1002/oby.20895

Hutchesson, M., Taylor, R., Vincze, L., Shrewsbury, V., Park, F., Campbell, L., Callister, R., & Collins, C. (2019). Be Healthe for your Heart Study Protocol: Preventing cardiovascular disease among women with a history of pre-eclampsia [Conference Abstract]. *Obesity Research and Clinical Practice*, *13*(3), 319. https://doi.org/10.1016/j.orcp.2018.11.228

Hutchesson, M. J., Tan, C. Y., Morgan, P., Callister, R., & Collins, C. (2016). Enhancement of Self-Monitoring in a Web-Based Weight Loss Program by Extra Individualized Feedback and Reminders: Randomized Trial [Article]. *Journal of medical Internet research*, *18*(4), e82. https://doi.org/10.2196/jmir.4100

Ienca, R., Giardiello, C., & Schiano, R. (2018). Virtual follow-up (FU) program enhances weight-loss results post Elipse balloon: An innovative, digital patient-friendly approach [Conference Abstract]. *Obes Facts*, *11*, 49. https://doi.org/10.1159/000489691

Ifejika, N. L., Bhadane, M., Cai, C., Noser, E. A., & Savitz, S. I. (2019). Cluster Enrollment: A Screening Tool for Stroke Risk Factors in Minority Women Caregivers [Article]. *Journal of the National Medical Association*, *111*(3), 281-284. https://doi.org/10.1016/j.jnma.2018.10.013

Ifejika, N. L., Noser, E. A., Grotta, J. C., & Savitz, S. I. (2016). Swipe out Stroke: Feasibility and efficacy of using a smart-phone based mobile application to improve compliance with weight loss in obese minority stroke patients and their carers. *International Journal of Stroke*, *11*(5), 593-603.

Ipjian, M. L., & Johnston, C. S. (2017). Smartphone technology facilitates dietary change in healthy adults [Randomized Controlled Trial]. *Nutrition*, *33*, 343-347.

Jaén, I., Suso-Ribera, C., Castilla, D., Zaragoza, I., García-Palacios, A., & Gómez Palones, J. L. (2019). Improving chronic pain management with eHealth and mHealth: study protocol for a randomised controlled trial [Article]. *BMJ Open*, *9*(12). https://doi.org/10.1136/bmjopen-2019-033586

Järvelä-Reijonen, E., Karhunen, L., Sairanen, E., Muotka, J., Lindroos, S., Laitinen, J., Puttonen, S., Peuhkuri, K., Hallikainen, M., Pihlajamäki, J., Korpela, R., Ermes, M., Lappalainen, R., & Kolehmainen, M. (2018). The effects of acceptance and commitment therapy on eating behavior and diet delivered through face-to-face contact and a mobile app: A randomized controlled trial [Article]. *International Journal of Behavioral Nutrition and Physical Activity*, *15*(1). https://doi.org/10.1186/s12966-018-0654-8

Jeffery, R. W., Sherwood, N. E., Brelje, K., Pronk, N. P., Boyle, R., Boucher, J. L., & Hase, K. (2003). Mail and phone interventions for weight loss in a managed-care setting: Weigh-To-Be one-year outcomes. *Int J Obes Relat Metab Disord*, *27*(12), 1584-1592. https://doi.org/10.1038/sj.ijo.0802473

Jiskoot, G., Benneheij, S. H., Beerthuizen, A., De Niet, J. E., De Klerk, C., Timman, R., Busschbach, J. J., & Laven, J. S. E. (2017). A three-component cognitive behavioural lifestyle program for preconceptional weight-loss in women with polycystic ovary syndrome (PCOS): A protocol for a randomized controlled trial [Article]. *Reproductive Health*, *14*(1). https://doi.org/10.1186/s12978-017-0295-4

Johansson, L., & Danielsson, P. (2019). A new web-based childhood obesity treatment with objective self-monitoring of weight, physical activity and continuous support from the clinic - A randomized controlled pilot study [Conference Abstract]. *Obes Facts*, *12*, 56. https://doi.org/10.1159/000489691

Johnston, C. A., Moreno, J. P., Hernandez, D. C., Link, B. A., Chen, T. A., Wojtanowski, A. C., Foster, G. D., & Foreyt, J. P. (2019). Levels of adherence needed to achieve significant weight loss. *Int J Obes (Lond)*, *43*(1), 125-131. https://doi.org/10.1038/s41366-018-0226-7

Joseph, R. P., Ainsworth, B. E., Vega-López, S., Adams, M. A., Hollingshead, K., Hooker, S. P., Todd, M., Gaesser, G. A., & Keller, C. (2019). Rationale and design of Smart Walk: A randomized controlled pilot trial of a smartphone-delivered physical activity and cardiometabolic risk reduction intervention for African American women [Article]. *Contemp Clin Trials*, *77*, 46-60. https://doi.org/10.1016/j.cct.2018.12.011

Kakoschke, N., Hawker, C., Castine, B., de Courten, B., & Verdejo-Garcia, A. (2018). Smartphone-based cognitive bias modification training improves healthy food choice in obesity: A pilot study [Article]. *European Eating Disorders Review*, *26*(5), 526-532. https://doi.org/10.1002/erv.2622

Kakoschke, N., Hawker, C., Castine, B., De Courten, B., & Verdejo-Garcia, A. (2019). Modification of cognitive biases in overweight and obesity [Conference Abstract]. *Obesity Research and Clinical Practice*, *13*(3), 268. https://doi.org/10.1016/j.orcp.2018.11.094

Kelly, J. T., Conley, M., Hoffmann, T., Craig, J. C., Tong, A., Reidlinger, D. P., Reeves, M. M., Howard, K., Krishnasamy, R., Kurtkoti, J., Palmer, S. C., Johnson, D. W., & Campbell, K. L. (2020). A Coaching Program to Improve Dietary Intake of Patients with CKD: ENTICE-CKD [Article]. *Clinical journal of the American Society of Nephrology : CJASN*, *15*(3), 330-340. https://doi.org/10.2215/CJN.12341019

Kennelly, M., Ainscough, K., O'Sullivan, E., Lindsay, K., McCarthy, M., Gibney, E., & McAuliffe, F. (2017). A randomised controlled trial of an M-health behavioural lifestyle intervention to prevent gestational diabetes in overweight and obese pregnancy: PEARS trial [Conference Abstract]. *BJOG: An International Journal of Obstetrics and Gynaecology*, *124*, 14. https://doi.org/10.1111/1471-0528.14585

Kennelly, M. A., Ainscough, K., Lindsay, K., Gibney, E., Mc Carthy, M., & McAuliffe, F. M. (2016). Pregnancy, exercise and nutrition research study with smart phone app support (Pears): Study protocol of a randomized controlled trial [Article]. *Contemp Clin Trials*, *46*, 92-99. https://doi.org/10.1016/j.cct.2015.11.018

Kennelly, M. A., Ainscough, K., Lindsay, K. L., OʼSullivan, E., Gibney, E. R., McCarthy, M., Segurado, R., DeVito, G., Maguire, O., Smith, T., Hatunic, M., & McAuliffe, F. M. (2018). Pregnancy Exercise and Nutrition With Smartphone Application Support: A Randomized Controlled Trial. *Obstet Gynecol*, *131*(5), 818-826. https://doi.org/10.1097/aog.0000000000002582

Kennelly, M. A., Ainscough, K., Philips, C. M., Alberdi, G., Lindsay, K. L., & McAuliffe, F. M. (2019). 1012: Maternal Inflammation: potential mediators and effects on pregnancy outcomes [Conference Abstract]. *American Journal of Obstetrics and Gynecology*, *220*(1), S650-S651. https://doi.org/10.1016/j.ajog.2018.11.1036

Kennelly, M. A., Ainscough, K. M., O'Sullivan, E. J., Lindsay, K. L., & McAuliffe, F. M. (2017). A randomized controlled trial of an M-health behavioural lifestyle intervention to prevent gestational diabetes in overweight and obese pregnancy: Pears [Conference Abstract]. *Reproductive Sciences*, *24*(1), 83A. https://doi.org/10.1177/1933719117699773

Kiernan, M., Moore, S. D., Schoffman, D. E., Lee, K., King, A. C., Taylor, C. B., Kiernan, N. E., & Perri, M. G. (2012). Social support for healthy behaviors: scale psychometrics and prediction of weight loss among women in a behavioral program. *Obesity (Silver Spring)*, *20*(4), 756-764. https://doi.org/10.1038/oby.2011.293

Kim, H. S., & Song, M. S. (2008). Technological intervention for obese patients with type 2 diabetes [Article]. *Applied Nursing Research*, *21*(2), 84-89. https://doi.org/10.1016/j.apnr.2007.01.007

Kim, S. I., & Kim, H. S. (2008). Effectiveness of mobile and internet intervention in patients with obese type 2 diabetes. *Int J Med Inform*, *77*(6), 399-404.

Kim, Y., Sysko, R., Michaeledes, A., Ramos, T., & Hildebrandt, T. (2019a). 92 Effects of Smartphone Coaching Intervention on Dietary Intake for Bariatric Surgery Candidates: A Pilot Randomized Controlled Trial [Article]. *CNS Spectrums*, *24*(1), 220-221. https://doi.org/10.1017/S1092852919000683

Kim, Y., Sysko, R., Michaeledes, A., Ramos, T., & Hildebrandt, T. (2019b). Effects of smartphone coaching intervention on dietary intake for bariatric surgery candidates: A pilot randomized controlled trial [Conference Abstract]. *CNS Spectrums*, *24*(1), 220-221. https://doi.org/10.1017/S1092852919000014

King, A. C., Hekler, E. B., Grieco, L. A., Winter, S. J., Sheats, J. L., Buman, M. P., Banerjee, B., Robinson, T. N., & Cirimele, J. (2016). Effects of Three Motivationally Targeted Mobile Device Applications on Initial Physical Activity and Sedentary Behavior Change in Midlife and Older Adults: A Randomized. *PLoS ONE [Electronic Resource]*, *11*(6), e0156370.

Kinoshita, S., Ryuzaki, M., Sone, M., Nishida, E., Nakamoto, H., & Group, F. S. (2014). Effectiveness of using long-acting angiotensin II type 1 receptor blocker in Japanese obese patients with metabolic syndrome on morning hypertension monitoring by using telemedicine system (FUJIYAMA study). *Clinical & Experimental Hypertension (New York)*, *36*(7), 508-516.

Kjær, I. G. H., Anderssen, S. A., & Torstveit, M. K. (2018). A tailored telephone and email based exercise intervention induced reductions in various measures of body composition in physically inactive adults: A randomized controlled trial [Article]. *Preventive Medicine Reports*, *11*, 160-168. https://doi.org/10.1016/j.pmedr.2018.06.011

Knäuper, B., Carrière, K., Frayn, M., Ivanova, E., Xu, Z., Ames-Bull, A., Islam, F., Lowensteyn, I., Sadikaj, G., Luszczynska, A., & Grover, S. (2018). The Effects of If-Then Plans on Weight Loss: Results of the McGill CHIP Healthy Weight Program Randomized Controlled Trial. *Obesity (Silver Spring)*, *26*(8), 1285-1295. https://doi.org/10.1002/oby.22226

Korinek, E. V., Phatak, S. S., Martin, C. A., Freigoun, M. T., Rivera, D. E., Adams, M. A., Klasnja, P., Buman, M. P., & Hekler, E. B. (2018). Adaptive step goals and rewards: a longitudinal growth model of daily steps for a smartphone-based walking intervention [Article]. *J Behav Med*, *41*(1), 74-86. https://doi.org/10.1007/s10865-017-9878-3

Kornman, K. P., Shrewsbury, V. A., Chou, A. C., Nguyen, B., Lee, A., O'Connor, J., Steinbeck, K. S., Hill, A. J., Kohn, M. R., Shah, S., & Baur, L. A. (2010). Electronic therapeutic contact for adolescent weight management: the Loozit study. *Telemedicine Journal & E-Health*, *16*(6), 678-685.

, T., Naud, S., & Harvey, J. R. (2019). The impact of the interventionist-participant relationship on treatment adherence and weight loss. *Transl Behav Med*, *9*(2), 368-372.

Kumagai, H., Zempo-Miyaki, A., Yoshikawa, T., Eto, M., So, R., Tsujimoto, T., Nishiyasu, T., Tanaka, K., & Maeda, S. (2018). Which cytokine is the most related to weight loss-induced decrease in arterial stiffness in overweight and obese men? *Endocr J*, *65*(1), 53-61. https://doi.org/10.1507/endocrj.EJ17-0117

Kurscheid, T., Redaélli, M., Heinen, A., Hahmann, P., Behle, K., & Froböse, I. (2019). App-controlled feedback devices can support sustainability of weight loss. Multicentre QUANT-study shows additional weight loss and gain of QoL via multiple feedback-devices in OPTIFAST®52-program [Article]. *Z Psychosom Med Psychother*, *65*(3), 224-238. https://doi.org/10.13109/zptm.2019.65.3.224

L'Allemand, D., Shih, C. H., Heldt, K., Büchter, D., Brogle, B., Rüegger, D., Filler, A., Gin-Drat, P., Durrer, D., Farpour-Lambert, N., & Kowatsch, T. (2018). Design and interim evaluation of a smartphone app for overweight adolescents using a behavioural health intervention platform [Conference Abstract]. *Obesity Reviews*, *19*, 102. https://doi.org/10.1111/(ISSN)1467-789X

Laitner, M. H., Minski, S. A., & Perri, M. G. (2016). The role of self-monitoring in the maintenance of weight loss success. *Eat Behav*, *21*, 193-197. https://doi.org/10.1016/j.eatbeh.2016.03.005

Lauti, M., Kularatna, M., Pillai, A., Hill, A. G., & MacCormick, A. D. (2018). A Randomised Trial of Text Message Support for Reducing Weight Regain Following Sleeve Gastrectomy [Article]. *Obes Surg*, *28*(8), 2178-2186. https://doi.org/10.1007/s11695-018-3176-1

Lavu, H., McCall, N. S., Winter, J. M., Burkhart, R. A., Pucci, M., Leiby, B. E., Yeo, T. P., Cannaday, S., & Yeo, C. J. (2019). Enhancing Patient Outcomes while Containing Costs after Complex Abdominal Operation: A Randomized Controlled Trial of the Whipple Accelerated Recovery Pathway [Article]. *Journal of the American College of Surgeons*, *228*(4), 415-424. https://doi.org/10.1016/j.jamcollsurg.2018.12.032

Lawrence, N. S., Van Beurden, S., Javaid, M., & Mostazir, M. M. (2018). Mass dissemination of web and smartphone-delivered food response inhibition training to reduce unhealthy snacking [Conference Abstract]. *Appetite*, *130*, 309. https://doi.org/10.1016/j.appet.2018.05.207

Leahey, T. M., & Wing, R. R. (2013). A randomized controlled pilot study testing three types of health coaches for obesity treatment: Professional, peer, and mentor. *Obesity (Silver Spring)*, *21*(5), 928-934. https://doi.org/10.1002/oby.20271

Lee, C. H., Cheung, B., Yi, G. H., Oh, B., & Oh, Y. H. (2018). Mobile health, physical activity, and obesity: Subanalysis of a randomized controlled trial [Article]. *Medicine (United States)*, *97*(38). https://doi.org/10.1097/MD.0000000000012309

Lee, H., Kane, I., Brar, J., & Sereika, S. (2014). Telephone-delivered physical activity intervention for individuals with serious mental illness: a feasibility study. *J Am Psychiatr Nurses Assoc*, *20*(6), 389-397. https://doi.org/10.1177/1078390314561497

Lee, R. L., Leung, C., Chen, H., Louie, L. H. T., Brown, M., Chen, J. L., Cheung, G., & Lee, P. H. (2017). The Impact of a School-Based Weight Management Program Involving Parents via mHealth for Overweight and Obese Children and Adolescents with Intellectual Disability: A Randomized Controlled Trial. *Int J Environ Res Public Health*, *14*(10). https://doi.org/10.3390/ijerph14101178

Lewis, A., Jolly, K., Adab, P., Daley, A., Farley, A., Jebb, S., Lycett, D., Clarke, S., Christian, A., Jin, J., Thompson, B., & Aveyard, P. (2013). A brief intervention for weight management in primary care: study protocol for a randomized controlled trial. *Trials*, *14*, 393. https://doi.org/10.1186/1745-6215-14-393

Lewis, E., Huang, H. C., Hassmen, P., Welvaert, M., & Pumpa, K. L. (2019). Adding Telephone and Text Support to an Obesity Management Program Improves Behavioral Adherence and Clinical Outcomes. A Randomized Controlled Crossover Trial [Randomized Controlled Trial]. *Int J Behav Med*, *26*(6), 580-590.

Lewis, K. (2014). Weight watchers for the facebook era-how does it compare to the do-it-yourself approach? [Note]. *Journal of Clinical Outcomes Management*, *21*(3), 102-105. http://www.embase.com/search/results?subaction=viewrecord&from=export&id=L373595072

Lim, K., Chi, C., Chan, S. Y., Lim, S. L., Ang, S. M., Yoong, J. S., Tsai, C., Wong, S. R., Yew, T. W., Tai, E. S., & Yong, E. L. (2019). Smart Phone APP to Restore Optimal Weight (SPAROW): protocol for a randomised controlled trial for women with recent gestational diabetes [Randomized Controlled Trial]. *BMC Public Health*, *19*(1), 1287.

Lim, S., Kang, S. M., Kim, K. M., Moon, J. H., Choi, S. H., Hwang, H., Jung, H. S., Park, K. S., Ryu, J. O., & Jang, H. C. (2016). Multifactorial intervention in diabetes care using real-time monitoring and tailored feedback in type 2 diabetes [Article]. *Acta Diabetologica*, *53*(2), 189-198. https://doi.org/10.1007/s00592-015-0754-8

Limaye, T., Kumaran, K., Joglekar, C., Bhat, D., Kulkarni, R., Nanivadekar, A., & Yajnik, C. (2017). Efficacy of a virtual assistance-based lifestyle intervention in reducing risk factors for Type 2 diabetes in young employees in the information technology industry in India: LIMIT, a randomized controlled trial [Article]. *Diabetic Medicine*, *34*(4), 563-568. https://doi.org/10.1111/dme.13258

Lin, M., Mahmooth, Z., Dedhia, N., Frutchey, R., Mercado, C. E., Epstein, D. H., Preston, K. L., Gibbons, M. C., Bowie, J. V., Labrique, A. B., & Cheskin, L. J. (2015). Tailored, interactive text messages for enhancing weight loss among African American adults: the TRIMM randomized controlled trial. *Am J Med*, *128*(8), 896-904. https://doi.org/10.1016/j.amjmed.2015.03.013

Lin, P. H., Intille, S., Bennett, G., Bosworth, H. B., Corsino, L., Voils, C., Grambow, S., Lazenka, T., Batch, B. C., Tyson, C., & Svetkey, L. P. (2015). Adaptive intervention design in mobile health: Intervention design and development in the Cell Phone Intervention for You trial [Article]. *Clinical Trials*, *12*(6), 634-645. https://doi.org/10.1177/1740774515597222

Lin, P. H., Wang, Y., Levine, E., Askew, S., Lin, S., Chang, C., Sun, J., Foley, P., Wang, H., Li, X., & Bennett, G. G. (2014). A text messaging-assisted randomized lifestyle weight loss clinical trial among overweight adults in Beijing [Article]. *Obesity*, *22*(5), E29-E37. https://doi.org/10.1002/oby.20686

Lindsay, E. K., Marsland, A. L., Young, S., Smyth, J. M., Brown, K. W., Gray, K., Walsh, C. P., & Creswell, J. D. (2019). Effects of two-week smartphone-based mindfulness training on markers of inflammation: A randomized controlled trial [Conference Abstract]. *Brain, Behavior, and Immunity*, *76*, e33. https://doi.org/10.1016/j.bbi.2018.11.277

Lindsay, E. K., Marsland, A. L., Young, S., Smyth, J. M., Brown, K. W., Gray, K., Walsh, C. P., & David Creswell, J. (2018). Two-week smartphone-based mindfulness training does not impact markers of inflammation: A randomized controlled trial [Conference Abstract]. *Psychosom Med*, *80*(3), A66. https://doi.org/10.1097/PSY.0000000000000578

Little, P., Stuart, B., Hobbs, F. R., Kelly, J., Smith, E. R., Bradbury, K. J., Hughes, S., Smith, P. W., Moore, M. V., Lean, M. E., Margetts, B. M., Byrne, C. D., Griffin, S., Davoudianfar, M., Hooper, J., Yao, G., Zhu, S., Raftery, J., & Yardley, L. (2016). An internet-based intervention with brief nurse support to manage obesity in primary care (POWeR+): a pragmatic, parallel-group, randomised controlled trial. *The Lancet Diabetes & Endocrinology*, *4*(10), 821-828.

Liu, Z., Wu, Y., Niu, W. Y., Feng, X., Lin, Y., Gao, A., Zhang, F., Fang, H., Gao, P., Li, H. J., & Wang, H. (2019). A school-based, multi-faceted health promotion programme to prevent obesity among children: Protocol of a cluster-randomised controlled trial (the DECIDE-Children study) [Article]. *BMJ Open*, *9*(11). https://doi.org/10.1136/bmjopen-2018-027902

Livingstone, K. M., Celis-Morales, C., Navas-Carretero, S., San-Cristoba, R., MacReady, A. L., Fallaize, R., Forster, H., Woolhead, C., O'Donovan, C. B., Marsaux, C. F. M., Kolossa, S., Tsirigoti, L., Lambrinou, C. P., Moschonis, G., Godlewska, M., Surwio, A., Drevon, C. A., Manios, Y., Traczyk, I., Gibney, E. R., Brennan, L., Walsh, M. C., Lovegrove, J. A., Saris, W. H., Daniel, H., Gibney, M., Martinez, J. A., & Mathers, J. C. (2016). Effect of an Internet-based, personalized nutrition randomized trial on dietary changes associated with the Mediterranean diet: The Food4Me Study [Article]. *American Journal of Clinical Nutrition*, *104*(2), 288-297. https://doi.org/10.3945/ajcn.115.129049

Llanos, A. A. M., Krok, J. L., Peng, J., Pennell, M. L., Vitolins, M. Z., Degraffinreid, C. R., & Paskett, E. D. (2014). Effects of a Walking Intervention Using Mobile Technology and Interactive Voice Response on Serum Adipokines Among Postmenopausal Women at Increased Breast Cancer Risk [Article]. *Hormones and Cancer*, *5*(2), 98-103. https://doi.org/10.1007/s12672-013-0168-4

Lozano-Lozano, M., Martín-Martín, L., Galiano-Castillo, N., Fernández-Lao, C., Cantarero-Villanueva, I., López-Barajas, I. B., & Arroyo-Morales, M. (2019). Mobile health and supervised rehabilitation versus mobile health alone in breast cancer survivors: Randomized controlled trial [Article in Press]. *Annals of Physical and Rehabilitation Medicine*. https://doi.org/10.1016/j.rehab.2019.07.007

Lubans, D. R., Smith, J. J., Morgan, P. J., Beauchamp, M. R., Miller, A., Lonsdale, C., Parker, P., & Dally, K. (2016). Mediators of psychological well-being in adolescent boys [Article]. *Journal of Adolescent Health*, *58*(2), 230-236. https://doi.org/10.1016/j.jadohealth.2015.10.010

Lubans, D. R., Smith, J. J., Plotnikoff, R. C., Dally, K. A., Okely, A. D., Salmon, J., & Morgan, P. J. (2016). Assessing the sustained impact of a school-based obesity prevention program for adolescent boys: The ATLAS cluster randomized controlled trial [Article]. *International Journal of Behavioral Nutrition and Physical Activity*, *13*(1). https://doi.org/10.1186/s12966-016-0420-8

Luley, C., Blaik, A., Götz, A., Kicherer, F., Kropf, S., Isermann, B., Stumm, G., & Westphal, S. (2014). Weight loss by telemonitoring of nutrition and physical activity in patients with metabolic syndrome for 1 year. *J Am Coll Nutr*, *33*(5), 363-374. https://doi.org/10.1080/07315724.2013.875437

Luley, C., Blaik, A., Reschke, K., Klose, S., & Westphal, S. (2011). Weight loss in obese patients with type 2 diabetes: effects of telemonitoring plus a diet combination - the Active Body Control (ABC) Program [Randomized Controlled Trial]. *Diabetes Research & Clinical Practice*, *91*(3), 286-292.

Luque, V., Feliu, A., Escribano, J., Ferré, N., Flores, G., Monné, R., Gutiérrez-Marín, D., Guillen, N., Muñoz-Hernando, J., Zaragoza-Jordana, M., Gispert-Llauradó, M., Rubio-Torrents, C., Núñez-Roig, M., Alcázar, M., Ferré, R., Basora, J. M., Hsu, P., Alegret-Basora, C., Arasa, F., Venables, M., Singh, P., & Closa-Monasterolo, R. (2019). The Obemat2.0 Study: A Clinical Trial of a Motivational Intervention for Childhood Obesity Treatment. *Nutrients*, *11*(2). https://doi.org/10.3390/nu11020419

Lutes, L. D., Daiss, S. R., Barger, S. D., Read, M., Steinbaugh, E., & Winett, R. A. (2012). Small changes approach promotes initial and continued weight loss with a phone-based follow-up: nine-month outcomes from ASPIRES II. *Am J Health Promot*, *26*(4), 235-238. https://doi.org/10.4278/ajhp.090706-QUAN-216

Lycett, K., Wittert, G., Gunn, J., Hutton, C., Clifford, S. A., & Wake, M. (2014). The challenges of real-world implementation of web-based shared care software: the HopSCOTCH Shared-Care Obesity Trial in Children. *BMC Med Inform Decis Mak*, *14*, 61. https://doi.org/10.1186/1472-6947-14-61

Lyons, E. J., Baranowski, T., Basen-Engquist, K. M., Lewis, Z. H., Swartz, M. C., Jennings, K., & Volpi, E. (2016). Testing the effects of narrative and play on physical activity among breast cancer survivors using mobile apps: Study protocol for a randomized controlled trial [Article]. *BMC Cancer*, *16*(1). https://doi.org/10.1186/s12885-016-2244-y

Madigan, C. D., Daley, A. J., Lewis, A. L., Jolly, K., & Aveyard, P. (2014). Which weight-loss programmes are as effective as Weight Watchers(R)?: non-inferiority analysis. *Br J Gen Pract*, *64*(620), e128-136. https://doi.org/10.3399/bjgp14X677491

Madjd, A., Taylor, M. A., Shafiei Neek, L., Delavari, A., Malekzadeh, R., Macdonald, I. A., & Farshchi, H. R. (2016). Effect of weekly physical activity frequency on weight loss in healthy overweight and obese women attending a weight loss program: a randomized controlled trial. *Am J Clin Nutr*, *104*(5), 1202-1208. https://doi.org/10.3945/ajcn.116.136408

Manning, P., Munasinghe, P. E., Bellae Papannarao, J., Gray, A. R., Sutherland, W., & Katare, R. (2019). Acute Weight Loss Restores Dysregulated Circulating MicroRNAs in Individuals Who Are Obese. *J Clin Endocrinol Metab*, *104*(4), 1239-1248. https://doi.org/10.1210/jc.2018-00684

Manuvinakurike, R., Velicer, W. F., & Bickmore, T. W. (2014). Automated indexing of Internet stories for health behavior change: weight loss attitude pilot study. *J Med Internet Res*, *16*(12), e285. https://doi.org/10.2196/jmir.3702

Marcus, B. H., Hartman, S. J., Larsen, B. A., Pekmezi, D., Dunsiger, S. I., Linke, S., Marquez, B., Gans, K. M., Bock, B. C., Mendoza-Vasconez, A. S., Noble, M. L., & Rojas, C. (2016). Pasos Hacia La Salud: a randomized controlled trial of an internet-delivered physical activity intervention for Latinas. *International Journal of Behavioral Nutrition & Physical Activity*, *13*, 62.

Marra, M. V., Lilly, C. L., Nelson, K. R., Woofter, D. R., & Malone, J. (2019). A pilot randomized controlled trial of a telenutrition weight loss intervention in middle-aged and older men with multiple risk factors for cardiovascular disease [Article]. *Nutrients*, *11*(2). https://doi.org/10.3390/nu11020229

Martin, C. K., Chellino, A., Correa, J. B., Johnson, W. D., & Church, T. S. (2010). Efficacy of an e-Health intervention at promoting weight loss through remote delivery of services: Preliminary results from a randomized controlled trial [Conference Abstract]. *Obesity Reviews*, *11*, 240. https://doi.org/10.1111/j.1467-789X.2010.00763-7.x

Martin, C. K., Miller, A. C., Thomas, D. M., Champagne, C. M., Han, H., & Church, T. (2015). Efficacy of SmartLoss, a smartphone-based weight loss intervention: results from a randomized controlled trial [Randomized Controlled Trial. *Obesity*, *23*(5), 935-942.

Martin, L., Coughlin, J. W., Clark, J. M., Dalcin, A., & Bennett, W. L. (2018). A pilot randomized controlled trial ofa remotely-delivered behavioral health coaching program to limit weight gain in pregnancy and reduce postpar-tum weight retention [Conference Abstract]. *J Gen Intern Med*, *33*(2), 94-95. http://www.embase.com/search/results?subaction=viewrecord&from=export&id=L622329789

Martin, S. S., Feldman, D. I., Blumenthal, R. S., Jones, S. R., Post, W. S., McKibben, R. A., Michos, E. D., Ndumele, C. E., Ratchford, E. V., Coresh, J., & Blaha, M. J. (2015). mActive: A randomized clinical trial of an automated mHealth intervention for physical activity promotion [Article]. *Journal of the American Heart Association*, *4*(11). https://doi.org/10.1161/JAHA.115.002239

Martineta, M., Agustina, R., Febriyanti, E., Putri, M., & Purnamasari, D. (2019). A balanced-sustainable calorie-restricted diet effect using “eats up”application on waist circumference and inflammatory marker among Indonesian obese women: Randomized clinical trial [Conference Abstract]. *Annals of Nutrition and Metabolism*, *75*(3), 62. https://doi.org/10.1159/000501751

Martinez-Brockman, J. L., Harari, N., Segura-Perez, S., Goeschel, L., & Perez-Escamilla, R. (2017). Impact of the lactation advice through texting can help (LATCH) randomized controlled trial [Conference Abstract]. *FASEB Journal*, *31*(1). http://www.embase.com/search/results?subaction=viewrecord&from=export&id=L616958985

Mason, A. E., Hecht, F. M., Daubenmier, J. J., Sbarra, D. A., Lin, J., Moran, P. J., Schleicher, S. G., Acree, M., Prather, A. A., & Epel, E. S. (2018). Weight Loss Maintenance and Cellular Aging in the Supporting Health Through Nutrition and Exercise Study. *Psychosom Med*, *80*(7), 609-619. https://doi.org/10.1097/psy.0000000000000616

Mason, C., de Dieu Tapsoba, J., Duggan, C., Wang, C. Y., Alfano, C. M., & McTiernan, A. (2019). Eating behaviors and weight loss outcomes in a 12-month randomized trial of diet and/or exercise intervention in postmenopausal women. *Int J Behav Nutr Phys Act*, *16*(1), 113. https://doi.org/10.1186/s12966-019-0887-1

Mâsse, L. C., Vlaar, J., Macdonald, J., Bradbury, J., Warshawski, T., Buckler, E. J., Hamilton, J., Ho, J., Buchholz, A., Morrison, K. M., & Ball, G. D. C. (2020). Aim2Be mHealth intervention for children with overweight and obesity: study protocol for a randomized controlled trial [Article]. *Trials*, *21*(1), 132. https://doi.org/10.1186/s13063-020-4080-2

Matthews, L., Pugmire, J., Moore, L., Kelson, M., McConnachie, A., McIntosh, E., Morgan-Trimmer, S., Murphy, S., Hughes, K., Coulman, E., Utkina-Macaskill, O., & Simpson, S. A. (2017). Study protocol for the 'HelpMeDoIt!' randomised controlled feasibility trial: An app, web and social support-based weight loss intervention for adults with obesity [Article]. *BMJ Open*, *7*(10). https://doi.org/10.1136/bmjopen-2017-017159

Mayer, J. S., Hees, K., Medda, J., Grimm, O., Asherson, P., Bellina, M., Colla, M., Ibáñez, P., Koch, E., Martinez-Nicolas, A., Muntaner-Mas, A., Rommel, A., Rommelse, N., de Ruiter, S., Ebner-Priemer, U. W., Kieser, M., Ortega, F. B., Thome, J., Buitelaar, J. K., Kuntsi, J., Ramos-Quiroga, J. A., Reif, A., & Freitag, C. M. (2018). Bright light therapy versus physical exercise to prevent co-morbid depression and obesity in adolescents and young adults with attention-deficit / hyperactivity disorder: Study protocol for a randomized controlled trial [Article]. *Trials*, *19*(1). https://doi.org/10.1186/s13063-017-2426-1

McRobbie, H., Hajek, P., Peerbux, S., Kahan, B. C., Eldridge, S., Trépel, D., Parrott, S., Griffiths, C., Snuggs, S., & Smith, K. M. (2019). Randomised controlled trial and economic evaluation of a task-based weight management group programme. *BMC Public Health*, *19*(1), 365. https://doi.org/10.1186/s12889-019-6679-3

Melchart, D., Wühr, E., & Weidenhammer, W. (2017). High and Low Responders in a Comprehensive Lifestyle Program for Weight Loss - Secondary Analysis of a Randomized Controlled Trial. *Complement Med Res*, *24*(4), 232-239. https://doi.org/10.1159/000475663

Memon, A. R., Masood, T., Awan, W. A., & Waqas, A. (2018). The effectiveness of an incentivized physical activity programme (Active student) among female medical students in Pakistan: A randomized controlled trial [Article]. *Journal of the Pakistan Medical Association*, *68*(10), 1438-1445. http://www.embase.com/search/results?subaction=viewrecord&from=export&id=L623955255

Mendoza, J. A., Baker, K. S., Moreno, M. A., Whitlock, K., Abbey-Lambertz, M., Waite, A., Colburn, T., & Chow, E. J. (2017). A Fitbit and Facebook mHealth intervention for promoting physical activity among adolescent and young adult childhood cancer survivors: A pilot study [Article]. *Pediatric Blood and Cancer*, *64*(12). https://doi.org/10.1002/pbc.26660

Mensorio, M. S., Cebolla-Martí, A., Rodilla, E., Palomar, G., Lisón, J. F., Botella, C., Fernández-Aranda, F., Jimenez-Murcia, S., & Baños, R. M. (2019). Analysis of the efficacy of an internet-based self-administered intervention (“Living Better”) to promote healthy habits in a population with obesity and hypertension: An exploratory randomized controlled trial [Article]. *Int J Med Inform*, *124*, 13-23. https://doi.org/10.1016/j.ijmedinf.2018.12.007

Merchant, G., Weibel, N., Patrick, K., Fowler, J. H., Norman, G. J., Gupta, A., Servetas, C., Calfas, K., Raste, K., Pina, L., Donohue, M., Griswold, W. G., & Marshall, S. (2014). Click "like" to change your behavior: a mixed methods study of college students' exposure to and engagement with Facebook content designed for weight loss. *J Med Internet Res*, *16*(6), e158.

Miller, K., Turró, R., Greve, J. W., Bakker, C. M., Buchwald, J. N., & Espinós, J. C. (2017). MILEPOST Multicenter Randomized Controlled Trial: 12-Month Weight Loss and Satiety Outcomes After pose (SM) vs. Medical Therapy. *Obes Surg*, *27*(2), 310-322. https://doi.org/10.1007/s11695-016-2295-9

Milsom, V. A., Middleton, K. M., & Perri, M. G. (2011). Successful long-term weight loss maintenance in a rural population. *Clin Interv Aging*, *6*, 303-309. https://doi.org/10.2147/cia.S25389

Mokhtari, F., Rejeski, W. J., Zhu, Y., Wu, G., Simpson, S. L., Burdette, J. H., & Laurienti, P. J. (2018). Dynamic fMRI networks predict success in a behavioral weight loss program among older adults. *Neuroimage*, *173*, 421-433. https://doi.org/10.1016/j.neuroimage.2018.02.025

Moldovan, C. P., Weldon, A. J., Daher, N. S., Schneider, L. E., Bellinger, D. L., Berk, L. S., Hermé, A. C., Aréchiga, A. L., Davis, W. L., & Peters, W. R. (2016). Effects of a meal replacement system alone or in combination with phentermine on weight loss and food cravings. *Obesity (Silver Spring)*, *24*(11), 2344-2350. https://doi.org/10.1002/oby.21649

Morgan, P. J., Collins, C. E., Plotnikoff, R. C., McElduff, P., Burrows, T., Warren, J. M., Young, M. D., Berry, N., Saunders, K. L., Aguiar, E. J., & Callister, R. (2010). The SHED-IT community trial study protocol: a randomised controlled trial of weight loss programs for overweight and obese men [Article]. *BMC Public Health*, *10*, 701. http://www.embase.com/search/results?subaction=viewrecord&from=export&id=L360278131

Morgan, P. J., Scott, H. A., Young, M. D., Plotnikoff, R. C., Collins, C. E., & Callister, R. (2014). Associations between program outcomes and adherence to Social Cognitive theory tasks: process evaluation of the SHED-IT community weight loss trial for men. *Int J Behav Nutr Phys Act*, *11*, 89. https://doi.org/10.1186/s12966-014-0089-9

Morrison, L. G., Hargood, C., Lin, S. X., Dennison, L., Joseph, J., Hughes, S., Michaelides, D. T., Johnston, D., Johnston, M., Michie, S., Little, P., Smith, P. W., Weal, M. J., & Yardley, L. (2014). Understanding usage of a hybrid website and smartphone app for weight management: a mixed-methods study [Randomized Controlled Trial. *J Med Internet Res*, *16*(10), e201.

Morton, J., Khoury, H., Azagury, D., & Rivas, H. (2017). Early experience with low-dose phentermine for preoperative bariatric weight loss: A prospective randomized trial [Conference Abstract]. *Surgery for Obesity and Related Diseases*, *13*(10), S63. http://www.embase.com/search/results?subaction=viewrecord&from=export&id=L619618683

Motie, M., Evangelista, L. S., Lombardo, D., Hoi, J., Horwich, T. B., Hamilton, M., & Fonarow, G. C. (2017). Effect of weight loss on renal function in overweight and obese patients with heart failure. *Diabetes Metab Syndr*, *11*(2), 95-98. https://doi.org/10.1016/j.dsx.2016.06.026

Mousa, A. Y., Broce, M., Davis, E., McKee, B., & Yacoub, M. (2017). Telehealth electronic monitoring to reduce postdischarge complications and surgical site infections after arterial revascularization with groin incision [Article]. *Journal of Vascular Surgery*, *66*(6), 1902-1908. https://doi.org/10.1016/j.jvs.2017.07.063

Mummah, S., Robinson, T. N., Mathur, M., Farzinkhou, S., Sutton, S., & Gardner, C. D. (2017). Effect of a mobile app intervention on vegetable consumption in overweight adults: A randomized controlled trial [Article]. *International Journal of Behavioral Nutrition and Physical Activity*, *14*(1). https://doi.org/10.1186/s12966-017-0563-2

Mummah, S. A., King, A. C., Gardner, C. D., & Sutton, S. (2016). Iterative development of Vegethon: a theory-based mobile app intervention to increase vegetable consumption. *International Journal of Behavioral Nutrition & Physical Activity*, *13*, 90.

Murphy, P. J., & Williams, R. L. (2013). Weight-loss study in African-American Women: lessons learned from project take HEED and future, technologically enhanced directions. *Perm J*, *17*(2), 55-59. https://doi.org/10.7812/tpp/12-094

Naets, T., Vervoort, L., Tanghe, A., & Braet, C. (2020). Adherence and barriers in e-health self-control training for enhancing childhood multidisciplinary obesity treatment [Article]. *Clinical psychology & psychotherapy*, *27*(1), 42-51. https://doi.org/10.1002/cpp.2405

Nakade, M., Aiba, N., Suda, N., Morita, A., Miyachi, M., Sasaki, S., Watanabe, S., & Group, S. (2012). Behavioral change during weight loss program and one-year follow-up: Saku Control Obesity Program (SCOP) in Japan. *Asia Pac J Clin Nutr*, *21*(1), 22-34.

Napolitano, M. A., Hayes, S., Bennett, G. G., Ives, A. K., & Foster, G. D. (2013). Using Facebook and text messaging to deliver a weight loss program to college students. *Obesity (Silver Spring)*, *21*(1), 25-31. https://doi.org/10.1002/oby.20232

Napolitano, M. A., Whiteley, J. A., Mavredes, M. N., Faro, J., DiPietro, L., Hayman, L. L., Neighbors, C. J., & Simmens, S. (2017). Using social media to deliver weight loss programming to young adults: Design and rationale for the Healthy Body Healthy U (HBHU) trial. *Contemp Clin Trials*, *60*, 1-13. https://doi.org/10.1016/j.cct.2017.06.007

Nelligan, R. K., Hinman, R. S., Kasza, J., Schwartz, S., Kimp, A., Atkins, L., & Bennell, K. L. (2019). Effect of a short message service (SMS) intervention on adherence to a physiotherapist-prescribed home exercise program for people with knee osteoarthritis and obesity: Protocol for the ADHERE randomised controlled trial [Article]. *BMC Musculoskeletal Disorders*, *20*(1). https://doi.org/10.1186/s12891-019-2801-z

Neumeier, W. H., Guerra, N., Thirumalai, M., Geer, B., Ervin, D., & Rimmer, J. H. (2017). POWERS forID: Personalized Online Weight and Exercise Response System for Individuals with Intellectual Disability: Study protocol for a randomized controlled trial [Article]. *Trials*, *18*(1). https://doi.org/10.1186/s13063-017-2239-2

Newton, R. L., Carter, L. A., Johnson, W., Zhang, D., Larrivee, S., Kennedy, B. M., Harris, M., & Hsia, D. S. (2018). A Church-Based Weight Loss Intervention in African American Adults using Text Messages (LEAN Study): Cluster Randomized Controlled Trial [Article]. *J Med Internet Res*, *20*(8), e256. https://doi.org/10.2196/jmir.9816

Nezami, B. T., Lytle, L. A., & Tate, D. F. (2016). A randomized trial to reduce sugar-sweetened beverage and juice intake in preschool-aged children: description of the Smart Moms intervention trial [Article]. *BMC Public Health*, *16*(1), 837. https://doi.org/10.1186/s12889-016-3533-8

Nezami, B. T., Ward, D. S., Lytle, L. A., Ennett, S. T., & Tate, D. F. (2018). A mHealth randomized controlled trial to reduce sugar-sweetened beverage intake in preschool-aged children [Article]. *Pediatr Obes*, *13*(11), 668-676. https://doi.org/10.1111/ijpo.12258

Ng, K. Y. B., Wellstead, S., Cheong, Y., & Macklon, N. (2018). A randomised controlled trial of a personalised lifestyle coaching application in modifying periconceptional behaviours in women suffering from reproductive failures (iPLAN trial) [Article]. *BMC Women's Health*, *18*(1). https://doi.org/10.1186/s12905-018-0689-7

Nicklas, J., Leiferman, J., Bull, S., Hazel, C., Gilbert, S., Hovey, D., & Barbour, L. (2016). Beta-testing a mobile health program designed to increase postpartum weight loss in women at elevated risk for cardiometabolic disease [Conference Abstract]. *Journal of Alternative and Complementary Medicine*, *22*(6), A140. https://doi.org/10.1089/acm.2016.29003.abstracts

Niklowitz, P., Rothermel, J., Lass, N., Barth, A., & Reinehr, T. (2018). Link between chemerin, central obesity, and parameters of the Metabolic Syndrome: findings from a longitudinal study in obese children participating in a lifestyle intervention. *Int J Obes (Lond)*, *42*(10), 1743-1752. https://doi.org/10.1038/s41366-018-0157-3

Nikolaou, C., & Lean, M. (2017). Young people's experiences and attitudes on the use and retention of lifestyle apps in six countries. A qualitative study [Conference Abstract]. *Obes Facts*, *10*, 155. https://doi.org/10.1159/000468958

Norman, G. J., Kolodziejczyk, J. K., Adams, M. A., Patrick, K., & Marshall, S. J. (2013). Fruit and vegetable intake and eating behaviors mediate the effect of a randomized text-message based weight loss program. *Prev Med*, *56*(1), 3-7. https://doi.org/10.1016/j.ypmed.2012.10.012

Nyström, E., Asklund, I., Sjöström, M., Stenlund, H., & Samuelsson, E. (2015). High expectations for pelvic floor muscle training with mobile application predicts successful treatment in women with stress urinary incontinence [Conference Abstract]. *Neurourology and Urodynamics*, *34*, S448-S449. https://doi.org/10.1002/nau.22830

O'Brien, K. M., Hutchesson, M. J., Jensen, M., Morgan, P., Callister, R., & Collins, C. E. (2014). Participants in an online weight loss program can improve diet quality during weight loss: a randomized controlled trial. *Nutr J*, *13*, 82. https://doi.org/10.1186/1475-2891-13-82

O'Brien, K. M., Wiggers, J., Williams, A., Campbell, E., Hodder, R. K., Wolfenden, L., Yoong, S. L., Robson, E. K., Haskins, R., Kamper, S. J., Rissel, C., & Williams, C. M. (2018). Telephone-based weight loss support for patients with knee osteoarthritis: a pragmatic randomised controlled trial. *Osteoarthritis Cartilage*, *26*(4), 485-494. https://doi.org/10.1016/j.joca.2018.01.003

O'Malley, G., Clarke, M., Burls, A., Murphy, S., Murphy, N., & Perry, I. J. (2014). A smartphone intervention for adolescent obesity: study protocol for a randomised controlled non-inferiority trial *Trials [Electronic Resource]*, *15*, 43.

O'Neil, P. M., Theim, K. R., Boeka, A., Johnson, G., & Miller-Kovach, K. (2012). Changes in weight control behaviors and hedonic hunger during a 12-week commercial weight loss program. *Eat Behav*, *13*(4), 354-360. https://doi.org/10.1016/j.eatbeh.2012.06.002

O'Neill, J., Daniel, T. O., & Epstein, L. H. (2016). Episodic future thinking reduces eating in a food court *Eat Behav*, *20*, 9-13.

Oddone, E. Z., Gierisch, J. M., Sanders, L. L., Fagerlin, A., Sparks, J., McCant, F., May, C., Olsen, M. K., & Damschroder, L. J. (2018). A Coaching by Telephone Intervention on Engaging Patients to Address Modifiable Cardiovascular Risk Factors: a Randomized Controlled Trial. *J Gen Intern Med*, *33*(9), 1487-1494. https://doi.org/10.1007/s11606-018-4398-6

Oddsson, S. (2017). Effects of a gamified mobile application to support a lifestyle-change program in adults: A controlled pilot [Conference Abstract]. *Diabetes*, *66*, A42. http://www.embase.com/search/results?subaction=viewrecord&from=export&id=L616960707

Olmos-Ochoa, T. T., Niv, N., Hellemann, G., Cohen, A. N., Oberman, R., Goldberg, R., & Young, A. S. (2019). Barriers to participation in web-based and in-person weight management interventions for serious mental illness. *Psychiatr Rehabil J*, *42*(3), 220-228. https://doi.org/10.1037/prj0000363

Olson, C. M., Groth, S. W., Graham, M. L., Reschke, J. E., Strawderman, M. S., & Fernandez, I. D. (2018). The effectiveness of an online intervention in preventing excessive gestational weight gain: The e-moms roc randomized controlled trial [Article]. *BMC Pregnancy Childbirth*, *18*(1). https://doi.org/10.1186/s12884-018-1767-4

Olson, C. M., Strawderman, M. S., & Graham, M. L. (2017). Association between consistent weight gain tracking and gestational weight gain: Secondary analysis of a randomized trial [Randomized Controlled Trial]. *Obesity*, *25*(7), 1217-1227. http://ovidsp.ovid.com/ovidweb.cgi?T=JS&CSC=Y&NEWS=N&PAGE=fulltext&D=med14&AN=28573669

http://vp9py7xf3h.search.serialssolutions.com/?sid=OVID:medline&id=pmid:28573669&id=doi:10.1002%2Foby.21873&issn=1930-7381&isbn=&volume=25&issue=7&spage=1217&pages=1217-1227&date=2017&title=Obesity&atitle=Association+between+consistent+weight+gain+tracking+and+gestational+weight+gain%3A+Secondary+analysis+of+a+randomized+trial.&aulast=Olson&pid=%3Cauthor%3EOlson+CM%3BStrawderman+MS%3BGraham+ML%3C%2Fauthor%3E%3CAN%3E28573669%3C%2FAN%3E%3CDT%3EJournal+Article%3C%2FDT%3E

Onoue, T., Goto, M., Kobayashi, T., Tominaga, T., Ando, M., Honda, H., Yoshida, Y., Tosaki, T., Yokoi, H., Kato, S., Maruyama, S., & Arima, H. (2018). Randomized controlled trial for assessment of internet of things system to guide intensive glucose control in diabetes outpatients: Nagoya health navigator study [Conference Abstract]. *Endocrine Reviews*, *39*(2). http://www.embase.com/search/results?subaction=viewrecord&from=export&id=L623113107

Oreskovic, N. M., Fletcher, R., Sharifi, M., Knutsen, J. D., Chilingirian, A., & Taveras, E. M. (2016). Design and rationale of the STRIVE trial to improve cardiometabolic health among children and families. *Contemp Clin Trials*, *49*, 149-154. https://doi.org/10.1016/j.cct.2016.07.012

Orsama, A. L., Lahteenmaki, J., Harno, K., Kulju, M., Wintergerst, E., Schachner, H., Stenger, P., Leppanen, J., Kaijanranta, H., Salaspuro, V., & Fisher, W. A. (2013). Active assistance technology reduces glycosylated hemoglobin and weight in individuals with type 2 diabetes: results of a theory-based randomized trial. *Diabetes Technol Ther*, *15*(8), 662-669.

Oshakbayev, K., Bimbetov, B., Manekenova, K., Bedelbayeva, G., Mustafin, K., & Dukenbayeva, B. (2019). Severe nonalcoholic steatohepatitis and type 2 diabetes: liver histology after weight loss therapy in a randomized clinical trial. *Curr Med Res Opin*, *35*(1), 157-165. https://doi.org/10.1080/03007995.2018.1547696

Oshima, Y., Matsuoka, Y., & Sakane, N. (2013). Effect of weight-loss program using self-weighing twice a day and feedback in overweight and obese subject: a randomized controlled trial. *Obes Res Clin Pract*, *7*(5), e361-366. https://doi.org/10.1016/j.orcp.2012.01.003

Østbye, T., Stroo, M., Eisenstein, E. L., & Dement, J. M. (2016). The Effects of Two Workplace Weight Management Programs and Weight Loss on Health Care Utilization and Costs. *J Occup Environ Med*, *58*(2), 162-169. https://doi.org/10.1097/jom.0000000000000586

Padhye, N. S., & Jing, W. (2016). Effect of self-monitoring clusters on weight and hemoglobin A1c [Article]. *Conference proceedings : ... Annual International Conference of the IEEE Engineering in Medicine and Biology Society. IEEE Engineering in Medicine and Biology Society. Annual Conference*, *2016*, 275-278. https://doi.org/10.1109/EMBC.2016.7590693

Pagoto, S., Schneider, K. L., Whited, M. C., Oleski, J. L., Merriam, P., Appelhans, B., Ma, Y., Olendzki, B., Waring, M. E., Busch, A. M., Lemon, S., Ockene, I., & Crawford, S. (2013). Randomized controlled trial of behavioral treatment for comorbid obesity and depression in women: the Be Active Trial. *Int J Obes (Lond)*, *37*(11), 1427-1434. https://doi.org/10.1038/ijo.2013.25

Palacios, C., Torres, M., López, D., Trak-Fellermeier, M. A., Coccia, C., & Pérez, C. M. (2018). Effectiveness of the nutritional app “MyNutriCart” on food choices related to purchase and dietary behavior: A pilot randomized controlled trial [Article]. *Nutrients*, *10*(12). https://doi.org/10.3390/nu10121967

Palmeira, A. L., Marques, M. M., Encantado, J., Carvalho, S., Duarte, C., Ermes, M., Evans, E., Harjumaa, M., Heitmann, B. L., Huotari, V., Kinnunen, T., Matos, M., Palmeira, L., Scott, S., Sniehotta, F. F., Stubbs, R. J., & Teixeira, P. J. (2017). Evidence-based behaviour change in a weight loss maintenance e-Health program: The H2020 NoHoW intervention toolkit [Conference Abstract]. *Obes Facts*, *10*, 28-29. https://doi.org/10.1159/000468958

Palmeira, C. S., Mussi, F. C., Santos, C., Lima, M. L., Ladeia, A. M. T., & Silva, L. C. J. (2019). Effect of remote nursing monitoring on overweight in women: clinical trial. *Rev Lat Am Enfermagem*, *27*, e3129. https://doi.org/10.1590/1518-8345.2651.3129

Paranoan, A. N., Jutamulia, J., Prasetya, S. I., Mudjihartini, N., & Witjaksono, F. (2018). Comparison of hs-crp level between low calorie high protein to standard protein diet in obese individuals with weight cycling - a randomised trial [version 1; peer review: 1 not approved] [Article]. *F1000Research*, *7*. https://doi.org/10.12688/F1000RESEARCH.13342.1

Parekh, N., Henriksson, P., Delisle Nyström, C., Silfvernagel, K., Ruiz, J. R., Ortega, F. B., Pomeroy, J., & Löf, M. (2018). Associations of Parental Self-Efficacy With Diet, Physical Activity, Body Composition, and Cardiorespiratory Fitness in Swedish Preschoolers: Results From the MINISTOP Trial [Article]. *Health Educ Behav*, *45*(2), 238-246. https://doi.org/10.1177/1090198117714019

Parker, S. M., Stocks, N., Nutbeam, D., Thomas, L., Denney-Wilson, E., Zwar, N., Karnon, J., Lloyd, J., Noakes, M., Liaw, S. T., Lau, A., Osborne, R., & Harris, M. F. (2018). Preventing chronic disease in patients with low health literacy using eHealth and teamwork in primary healthcare: Protocol for a cluster randomised controlled trial [Article]. *BMJ Open*, *8*(6). https://doi.org/10.1136/bmjopen-2018-023239

Partridge, S. R., Allman-Farinelli, M., McGeechan, K., Balestracci, K., Wong, A. T. Y., Hebden, L., Harris, M. F., Bauman, A., & Phongsavan, P. (2016). Process evaluation of TXT2BFiT: A multi-component mHealth randomised controlled trial to prevent weight gain in young adults [Article]. *International Journal of Behavioral Nutrition and Physical Activity*, *13*(1). https://doi.org/10.1186/s12966-016-0329-2

Partridge, S. R., Hebdren, L., Balestracci, K., Wong, A., Phongsavan, P., Denney-Wilson, E., Harris, M., McGeechan, K., Bauman, A., & Allman-Farinelli, M. (2014). Recruiting young adults into the TXT2BFiT trial for prevention of weight gain: Effectiveness and cost strategies [Conference Abstract]. *Obesity Research and Clinical Practice*, *8*, 77. https://doi.org/org/10.1016/j.orcp.2014.10.139

Partridge, S. R., McGeechan, K., Bauman, A., Phongsavan, P., & Allman-Farinelli, M. (2016). Improved eating behaviours mediate weight gain prevention of young adults: Moderation and mediation results of a randomised controlled trial of TXT2BFiT, mHealth program [Article]. *International Journal of Behavioral Nutrition and Physical Activity*, *13*(1). https://doi.org/10.1186/s12966-016-0368-8

Partridge, S. R., McGeechan, K., Bauman, A., Phongsavan, P., & Allman-Farinelli, M. (2017). Improved confidence in performing nutrition and physical activity behaviours mediates behavioural change in young adults: Mediation results of a randomised controlled mHealth intervention [Article]. *Appetite*, *108*, 425-433. https://doi.org/10.1016/j.appet.2016.11.005

Patel, M. L., Hopkins, C. M., & Bennett, G. G. (2019). Early weight loss in a standalone mHealth intervention predicting treatment success [Article]. *Obesity Science and Practice*, *5*(3), 231-237. https://doi.org/10.1002/osp4.329

Patrick, K., Marshall, S. J., Davila, E. P., Kolodziejczyk, J. K., Fowler, J. H., Calfas, K. J., Huang, J. S., Rock, C. L., Griswold, W. G., Gupta, A., Merchant, G., Norman, G. J., Raab, F., Donohue, M. C., Fogg, B. J., & Robinson, T. N. (2014). Design and implementation of a randomized controlled social and mobile weight loss trial for young adults (project SMART) *Contemp Clin Trials*, *37*(1), 10-18.

Patrick, K., Norman, G. J., Davila, E. P., Calfas, K. J., Raab, F., Gottschalk, M., Sallis, J. F., Godbole, S., & Covin, J. R. (2013). Outcomes of a 12-month technology-based intervention to promote weight loss in adolescents at risk for type 2 diabetes *Journal of Diabetes Science & Technology*, *7*(3), 759-770.

Patrick, K., Raab, F., Adams, M. A., Dillon, L., Zabinski, M., Rock, C. L., Griswold, W. G., & Norman, G. J. (2009). A text message-based intervention for weight loss: randomized controlled trial [Article]. *J Med Internet Res*, *11*(1), e1. https://doi.org/10.2196/jmir.1100

Pawalia, A., Kulandaivelan, S., Savant, S., & Yadav, V. S. (2017). Exercise in pregnancy: Effect on obesity parameters in indian women - A randomized controlled trial [Article]. *Romanian Journal of Diabetes, Nutrition and Metabolic Diseases*, *24*(4), 315-323. https://doi.org/10.1515/rjdnmd-2017-0037

Pekkarinen, T., Kaukua, J., & Mustajoki, P. (2015). Long-term weight maintenance after a 17-week weight loss intervention with or without a one-year maintenance program: a randomized controlled trial. *J Obes*, *2015*, 651460. https://doi.org/10.1155/2015/651460

Pekmezi, D., Ainsworth, C., Holly, T., Williams, V., Benitez, T., Wang, K., Rogers, L. Q., Marcus, B., & Demark-Wahnefried, W. (2017). Rationale, design, and baseline findings from a pilot randomized trial of an IVR-Supported physical activity intervention for cancer prevention in the Deep South: The DIAL study [Article]. *Contemporary Clinical Trials Communications*, *8*, 218-226. https://doi.org/10.1016/j.conctc.2017.10.008

Pellegrini, C. A., Conroy, D. E., Phillips, S. M., Pfammatter, A. F., McFadden, H. G., & Spring, B. (2018). Daily and Seasonal Influences on Dietary Self-monitoring Using a Smartphone Application [Article]. *Journal of nutrition education and behavior*, *50*(1), 56-61. https://doi.org/10.1016/j.jneb.2016.12.004

Pellegrini, C. A., Duncan, J. M., Moller, A. C., Buscemi, J., Sularz, A., DeMott, A., Pictor, A., Pagoto, S., Siddique, J., & Spring, B. (2012). A smartphone-supported weight loss program: design of the ENGAGED randomized controlled trial [Article]. *BMC Public Health*, *12*, 1041. http://www.embase.com/search/results?subaction=viewrecord&from=export&id=L366387487

Pellegrini, C. A., Hoffman, S. A., Collins, L. M., & Spring, B. (2014). Optimization of remotely delivered intensive lifestyle treatment for obesity using the Multiphase Optimization Strategy: Opt-IN study protocol *Contemp Clin Trials*, *38*(2), 251-259.

Peter, I., McCaffery, J. M., Kelley-Hedgepeth, A., Hakonarson, H., Reis, S., Wagenknecht, L. E., Kopin, A. S., & Huggins, G. S. (2012). Association of type 2 diabetes susceptibility loci with one-year weight loss in the look AHEAD clinical trial. *Obesity (Silver Spring)*, *20*(8), 1675-1682. https://doi.org/10.1038/oby.2012.11

Peters, J. C., Wyatt, H. R., Foster, G. D., Pan, Z., Wojtanowski, A. C., Vander Veur, S. S., Herring, S. J., Brill, C., & Hill, J. O. (2014). The effects of water and non-nutritive sweetened beverages on weight loss during a 12-week weight loss treatment program. *Obesity (Silver Spring)*, *22*(6), 1415-1421. https://doi.org/10.1002/oby.20737

Petrella, R. J., Gill, D. P., Zou, G., A, D. E. C., Riggin, B., Bartol, C., Danylchuk, K., Hunt, K., Wyke, S., Gray, C. M., Bunn, C., & Zwarenstein, M. (2017). Hockey Fans in Training: A Pilot Pragmatic Randomized Controlled Trial [Article]. *Medicine and science in sports and exercise*, *49*(12), 2506-2516. https://doi.org/10.1249/MSS.0000000000001380

Petrella, R. J., Stuckey, M. I., Shapiro, S., & Gill, D. P. (2014). Mobile health, exercise and metabolic risk: a randomized controlled trial [Article]. *BMC Public Health*, *14*, 1082. https://doi.org/10.1186/1471-2458-14-1082

Pfammatter, A. F., Nahum-Shani, I., DeZelar, M., Scanlan, L., McFadden, H. G., Siddique, J., Hedeker, D., & Spring, B. (2019). SMART: Study protocol for a sequential multiple assignment randomized controlled trial to optimize weight loss management [Article]. *Contemp Clin Trials*, *82*, 36-45. https://doi.org/10.1016/j.cct.2019.05.007

Phelan, S., Hagobian, T. A., Ventura, A., Brannen, A., Erickson-Hatley, K., Schaffner, A., Muñoz-Christian, K., Mercado, A., & Tate, D. F. (2019). 'Ripple' effect on infant zBMI trajectory of an internet-based weight loss program for low-income postpartum women. *Pediatr Obes*, *14*(1). https://doi.org/10.1111/ijpo.12456

Pieniak, Z., Żakowska-Biemans, S., Kostyra, E., & Raats, M. (2016). Sustainable healthy eating behaviour of young adults: towards a novel methodological approach [Article]. *BMC Public Health*, *16*, 577. https://doi.org/10.1186/s12889-016-3260-1

Pigeyre, M., Lelorain, S., Couturier, E., Guilbert, E., Deghilage, S., & Deruelle, P. (2016). Electronic-personalized program for obesity during pregnancy (ePPOP) [Conference Abstract]. *Obesity Reviews*, *17*, 175-176. https://doi.org/10.1111/obr.12403

Pinto, A. M., Fava, J. L., Hoffmann, D. A., & Wing, R. R. (2013). Combining behavioral weight loss treatment and a commercial program: a randomized clinical trial. *Obesity (Silver Spring)*, *21*(4), 673-680. https://doi.org/10.1002/oby.20044

Podina, I. R., Fodor, L. A., Cosmoiu, A., & Boian, R. (2017). An evidence-based gamified mHealth intervention for overweight young adults with maladaptive eating habits: Study protocol for a randomized controlled trial [Article]. *Trials*, *18*(1). https://doi.org/10.1186/s13063-017-2340-6

Pollak, K. I., Alexander, S. C., Bennett, G., Lyna, P., Coffman, C. J., Bilheimer, A., Farrell, D., Bodner, M. E., Swamy, G. K., & Østbye, T. (2014). Weight-related SMS texts promoting appropriate pregnancy weight gain: A pilot study [Article]. *Patient Educ Couns*, *97*(2), 256-260. https://doi.org/10.1016/j.pec.2014.07.030

Price, S., Ferisin, S., Sharifi, M., Steinberg, D., Bennett, G., Wolin, K. Y., Horan, C., Koziol, R., Marshall, R., & Taveras, E. M. (2015). Development and Implementation of an Interactive Text Messaging Campaign to Support Behavior Change in a Childhood Obesity Randomized Controlled Trial. *J Health Commun*, *20*(7), 843-850. https://doi.org/10.1080/10810730.2015.1018582

Priscilla, S., Nanditha, A., Simon, M., Satheesh, K., Kumar, S., Shetty, A. S., Snehalatha, C., Johnston, D. G., Godsland, I. F., Wareham, N. J., & Ramachandran, A. (2015). A pragmatic and scalable strategy using mobile technology to promote sustained lifestyle changes to prevent type 2 diabetes in India-Outcome of screening [Randomized Controlled Trial]. *Diabetes Research & Clinical Practice*, *110*(3), 335-340.

Ptomey, L. T., Washburn, R. A., Mayo, M. S., Greene, J. L., Lee, R. H., Szabo-Reed, A. N., Honas, J. J., Sherman, J. R., & Donnelly, J. E. (2018). Remote delivery of weight management for adults with intellectual and developmental disabilities: Rationale and design for a 24month randomized trial *Contemp Clin Trials*, *73*, 16-26.

Rafols, J. P., De La Cruz, J., Ubach, A. G., & Segarra, M. (2014). Personalized online monitoring using e-health technology for postoperative follow up after bariatric surgery. A randomized controlled trial [Conference Abstract]. *Obes Surg*, *24*(8), 1313-1314. https://doi.org/10.1007/s11695-014-1292-0

Rashad, N. M., Sayed, S. E., Sherif, M. H., & Sitohy, M. Z. (2019). Effect of a 24-week weight management program on serum leptin level in correlation to anthropometric measures in obese female: A randomized controlled clinical trial. *Diabetes Metab Syndr*, *13*(3), 2230-2235. https://doi.org/10.1016/j.dsx.2019.05.027

Raynor, H. A., Li, F., & Cardoso, C. (2018). Daily pattern of energy distribution and weight loss. *Physiol Behav*, *192*, 167-172. https://doi.org/10.1016/j.physbeh.2018.02.036

Raynor, H. A., Thomas, J. G., Cardoso, C. C., Wojtanowski, A. C., & Foster, G. D. (2019). Examining the pattern of new foods and beverages consumed during obesity treatment to inform strategies for self-monitoring intake [Article]. *Appetite*, *132*, 147-153. https://doi.org/10.1016/j.appet.2018.10.018

Recio-Rodriguez, J. I., Gómez-Marcos, M. A., Agudo-Conde, C., Ramirez, I., Gonzalez-Viejo, N., Gomez-Arranz, A., Salcedo-Aguilar, F., Rodriguez-Sanchez, E., Alonso-Domínguez, R., Sánchez-Aguadero, N., Gonzalez-Sanchez, J., & Garcia-Ortiz, L. (2018). EVIDENT 3 Study: A randomized, controlled clinical trial to reduce inactivity and caloric intake in sedentary and overweight or obese people using a smartphone application: Study protocol [Article]. *Medicine (United States)*, *97*(2). https://doi.org/10.1097/MD.0000000000009633

Reichard, A., Saunders, M. D., Saunders, R. R., Donnelly, J. E., Lauer, E., Sullivan, D. K., & Ptomey, L. (2015). A comparison of two weight management programs for adults with mobility impairments. *Disabil Health J*, *8*(1), 61-69. https://doi.org/10.1016/j.dhjo.2014.08.002

Rejeski, W. J., Ambrosius, W. T., Burdette, J. H., Walkup, M. P., & Marsh, A. P. (2017). Community Weight Loss to Combat Obesity and Disability in At-Risk Older Adults. *J Gerontol A Biol Sci Med Sci*, *72*(11), 1547-1553. https://doi.org/10.1093/gerona/glw252

Rieder, J., Khan, U. I., Heo, M., Mossavar-Rahmani, Y., Blank, A. E., Strauss, T., Viswanathan, N., & Wylie-Rosett, J. (2013). Evaluation of a community-based weight management program for predominantly severely obese, difficult-to-reach, inner-city minority adolescents. *Child Obes*, *9*(4), 292-304. https://doi.org/10.1089/chi.2012.0147

Rimmer, J. H., Wang, E., Pellegrini, C. A., Lullo, C., & Gerber, B. S. (2013). Telehealth weight management intervention for adults with physical disabilities: a randomized controlled trial [Article]. *American journal of physical medicine & rehabilitation / Association of Academic Physiatrists*, *92*(12), 1084-1094. https://doi.org/10.1097/PHM.0b013e31829e780e

Ritzwoller, D. P., Glasgow, R. E., Sukhanova, A. Y., Bennett, G. G., Warner, E. T., Greaney, M. L., Askew, S., Goldman, J., Emmons, K. M., & Colditz, G. A. (2013). Economic analyses of the Be Fit Be Well program: a weight loss program for community health centers. *J Gen Intern Med*, *28*(12), 1581-1588. https://doi.org/10.1007/s11606-013-2492-3

Rock, C. L., Flatt, S. W., Pakiz, B., Taylor, K. S., Leone, A. F., Brelje, K., Heath, D. D., Quintana, E. L., & Sherwood, N. E. (2014). Weight loss, glycemic control, and cardiovascular disease risk factors in response to differential diet composition in a weight loss program in type 2 diabetes: a randomized controlled trial. *Diabetes Care*, *37*(6), 1573-1580. https://doi.org/10.2337/dc13-2900

Romero-Moraleda, B., Peinado Lozano, A. B., Morencos Martínez, E., López-Plaza, B., Gómez Candela, C., & Calderón Montero, F. J. (2015). Lipid profile response to weight loss program in overweight and obese patient is related with gender and age. *Nutr Hosp*, *31*(6), 2455-2464. https://doi.org/10.3305/nh.2015.31.6.8926

Rosas, L. G., Lv, N., Azar, K. M. J., Xiao, L., Hooker, S. P., Lewis, M. A., Zavella, P., Venditti, E. M., & Ma, J. (2018). HOMBRE: A randomized controlled trial to compare two approaches to weight loss for overweight and obese Latino men (Hombres con Opciones para Mejorar el Bienestar y bajar el Riesgo de Enfermedades cronicas; men with choices to improve well-being and decrease chronic disease risk). *Contemp Clin Trials*, *68*, 23-34.

Rubinstein, A., Miranda, J. J., Beratarrechea, A., Diez-Canseco, F., Kanter, R., Gutierrez, L., Bernabé-Ortiz, A., Irazola, V., Fernandez, A., Letona, P., Martínez, H., Ramirez-Zea, M., Alasino, A. A., Cuesta, L. L., Moscoso, B. N. B., Surichaqui, J. E., Estrada, L. P., Ramírez, C. M., de la Cruz, G. R., Drago, J. C. S., Loayza, J. A. Z., Carrara, C., Giardini, G., Guevara, J., Juárez, A. M., Salguero, J., Lewitan, D., & Urtasún, M. (2016). Effectiveness of an mHealth intervention to improve the cardiometabolic profile of people with prehypertension in low-resource urban settings in Latin America: A randomised controlled trial [Article]. *The Lancet Diabetes and Endocrinology*, *4*(1), 52-63. https://doi.org/10.1016/S2213-8587(15)00381-2

Russell-Minda, E., Jutai, J. W., Bradley, K., Chudyk, A., & Petrella, R. J. (2009). Health technologies for monitoring diabetes and reducing cardiovascular complications: A systematic review [Conference Abstract]. *Journal of Diabetes*, *1*, A206. https://doi.org/10.1111/j.1753-0407.2009.00020.x

Sakakibara, B. M., Eng, J. J., Benavente, O., Barr, S. I., Silverberg, N. D., Goldsmith, C. H., Yao, J., & Lear, S. A. (2014). A telehealth intervention to promote healthy lifestyles after stroke: the stroke coach protocol [Conference Abstract]. *Stroke*, *45*(12), e285. https://doi.org/10.1161/01.str.0000455918.04147.bf

Sakane, N., Kotani, K., Suganuma, A., Takahashi, K., Sato, J., Suzuki, S., Izumi, K., Kato, M., Noda, M., Nirengi, S., & Kuzuya, H. (2019). Prevention of Metabolic Syndrome by Telephone-Delivered Lifestyle Intervention in a Real-World Setting: Sub-Analysis of a Cluster-Randomized Trial [Article]. *Metabolic Syndrome and Related Disorders*, *17*(7), 355-361. https://doi.org/10.1089/met.2018.0130

Samuel-Hodge, C. D., Garcia, B. A., Johnston, L. F., Gizlice, Z., Ni, A., Cai, J., Kraschnewski, J. L., Gustafson, A. A., Norwood, A. F., Glasgow, R. E., Gold, A. D., Graham, J. W., Evenson, K. R., Trost, S., & Keyserling, T. C. (2013). Translation of a behavioral weight loss intervention for mid-life, low-income women in local health departments. *Obesity (Silver Spring)*, *21*(9), 1764-1773. https://doi.org/10.1002/oby.20317

Sangster, J., Furber, S., Allman-Farinelli, M., Phongsavan, P., Redfern, J., Haas, M., Church, J., Mark, A., & Bauman, A. (2015). Effectiveness of a pedometer-based telephone coaching program on weight and physical activity for people referred to a cardiac rehabilitation program: a randomized controlled trial. *J Cardiopulm Rehabil Prev*, *35*(2), 124-129. https://doi.org/10.1097/hcr.0000000000000082

Santos, I., Mata, J., Silva, M. N., Sardinha, L. B., & Teixeira, P. J. (2015). Predicting long-term weight loss maintenance in previously overweight women: a signal detection approach. *Obesity (Silver Spring)*, *23*(5), 957-964. https://doi.org/10.1002/oby.21082

Sasai, H., Ueda, K., Tsujimoto, T., Kobayashi, H., Sanbongi, C., Ikegami, S., & Nakata, Y. (2017). Dose-ranging pilot randomized trial of amino acid mixture combined with physical activity promotion for reducing abdominal fat in overweight adults [Article]. *Diabetes, Metabolic Syndrome and Obesity: Targets and Therapy*, *10*, 297-309. https://doi.org/10.2147/DMSO.S138084

Schierberl Scherr, A. E., McClure Brenchley, K. J., & Gorin, A. A. (2013). Examining a ripple effect: do spouses' behavior changes predict each other's weight loss? *J Obes*, *2013*, 297268. https://doi.org/10.1155/2013/297268

Schultz, A. T., Markowitz, J. T., Cousineau, T. M., Franko, D. L., & Laffel, L. M. (2013). Mobile health (mHealth) intervention called BodiMojo using text messaging aimed at healthy lifestyles for youth with diabetes (DM): A pilot Randomized Controlled Trial (RCT) [Conference Abstract]. *Diabetes*, *62*, A342. https://doi.org/10.2337/db13-859-1394

Scott, H. A., Gibson, P. G., Garg, M. L., Pretto, J. J., Morgan, P. J., Callister, R., & Wood, L. G. (2015). Determinants of weight loss success utilizing a meal replacement plan and/or exercise, in overweight and obese adults with asthma. *Respirology*, *20*(2), 243-250. https://doi.org/10.1111/resp.12423

Senarath, U., Katulanda, P., Fernando, D. N., Kalupahana, N. S., Partheepan, K., Jayawardena, R., Katulanda, G., & Dibley, M. J. (2019). mHealth nutrition and lifestyle intervention (mHENAL) to reduce cardiovascular disease risk in a middle-aged, overweight and obese population in Sri Lanka: Study protocol for a randomized controlled trial [Article]. *Contemporary Clinical Trials Communications*, *16*. https://doi.org/10.1016/j.conctc.2019.100453

Sevick, M. A., Woolf, K., Mattoo, A., Katz, S. D., Li, H., St-Jules, D. E., Jagannathan, R., Hu, L., Pompeii, M. L., Ganguzza, L., Li, Z., Sierra, A., Williams, S. K., & Goldfarb, D. S. (2018). The Healthy Hearts and Kidneys (HHK) study: Design of a 2 × 2 RCT of technology-supported self-monitoring and social cognitive theory-based counseling to engage overweight people with diabetes and chronic kidney disease in multiple lifestyle changes [Article]. *Contemp Clin Trials*, *64*, 265-273. https://doi.org/10.1016/j.cct.2017.08.020

Shapiro, J. R., Koro, T., Doran, N., Thompson, S., Sallis, J. F., Calfas, K., & Patrick, K. (2012). Text4Diet: A randomized controlled study using text messaging for weight loss behaviors [Article]. *Prev Med*, *55*(5), 412-417. https://doi.org/10.1016/j.ypmed.2012.08.011

Shaw, R. J., Bosworth, H. B., Silva, S. S., Lipkus, I. M., Davis, L. L., Sha, R. S., & Johnson, C. M. (2013). Mobile health messages help sustain recent weight loss *American Journal of Medicine*, *126*(11), 1002-1009.

Sherry, A., Geraghty, A. A., Ainscough, K. M., Kennelly, M., Lindsay, K. L., & McAuliffe, F. M. (2018). Inflammation and metabolic health in pregnant women with overweight and obesity, and the impact of a lifestyle intervention [Conference Abstract]. *Obes Facts*, *11*, 242. https://doi.org/10.1159/000489691

Sherwood, N. E., Jeffery, R. W., Pronk, N. P., Boucher, J. L., Hanson, A., Boyle, R., Brelje, K., Hase, K., & Chen, V. (2006). Mail and phone interventions for weight loss in a managed-care setting: weigh-to-be 2-year outcomes. *Int J Obes (Lond)*, *30*(10), 1565-1573. https://doi.org/10.1038/sj.ijo.0803295

Shikany, J. M., Thomas, A. S., Beasley, T. M., Lewis, C. E., & Allison, D. B. (2013). Randomized controlled trial of the Medifast 5 & 1 Plan for weight loss. *Int J Obes (Lond)*, *37*(12), 1571-1578. https://doi.org/10.1038/ijo.2013.43

Shin, D. W., Joh, H. K., Yun, J. M., Kwon, H. T., Lee, H., Min, H., Shin, J. H., Chung, W. J., Park, J. H., & Cho, B. (2016). Design and baseline characteristics of participants in the Enhancing Physical Activity and Reducing Obesity through Smartcare and Financial Incentives (EPAROSFI): A pilot randomized controlled trial [Article]. *Contemp Clin Trials*, *47*, 115-122. https://doi.org/10.1016/j.cct.2015.12.019

Shrewsbury, V. A., O'Connor, J., Steinbeck, K. S., Stevenson, K., Lee, A., Hill, A. J., Kohn, M. R., Shah, S., Torvaldsen, S., & Baur, L. A. (2009). A randomised controlled trial of a community-based healthy lifestyle program for overweight and obese adolescents: The Loozit® study protocol [Article]. *BMC Public Health*, *9*. https://doi.org/10.1186/1471-2458-9-119

Sidhu, M. S., Daley, A., & Jolly, K. (2016). Evaluation of a text supported weight maintenance programme 'Lighten Up Plus' following a weight reduction programme: randomised controlled trial. *Int J Behav Nutr Phys Act*, *13*, 19. https://doi.org/10.1186/s12966-016-0346-1

Silina, V., Tessma, M. K., Senkane, S., Krievina, G., & Bahs, G. (2017). Text messaging (SMS) as a tool to facilitate weight loss and prevent metabolic deterioration in clinically healthy overweight and obese subjects: a randomised controlled trial [Randomized Controlled Trial]. *Scand J Prim Health Care*, *35*(3), 262-270. http://ovidsp.ovid.com/ovidweb.cgi?T=JS&CSC=Y&NEWS=N&PAGE=fulltext&D=med14&AN=28812403

Sit, J. W. H., Chair, S. Y., Hui, S. S. C., Choi, K. C., Chan, A. W. K., Wong, E. M. L., & Cheng, H. Y. (2016). A smartphone-based exercise adherence intervention for people with metabolic syndrome: A feasibility pilot study [Conference Abstract]. *The Lancet*, *388*(SPEC.ISS 1), 64. http://www.embase.com/search/results?subaction=viewrecord&from=export&id=L621461943

Skau, J. K. H., Nordin, A. B. A., Cheah, J. C. H., Ali, R., Zainal, R., Aris, T., Ali, Z. M., Matzen, P., Biesma, R., Aagaard-Hansen, J., Hanson, M. A., & Norris, S. A. (2016). A complex behavioural change intervention to reduce the risk of diabetes and prediabetes in the pre-conception period in Malaysia: Study protocol for a randomised controlled trial [Article]. *Trials*, *17*(1). https://doi.org/10.1186/s13063-016-1345-x

Small, L., Bonds-McClain, D., Melnyk, B., Vaughan, L., & Gannon, A. M. (2014). The preliminary effects of a primary care-based randomized treatment trial with overweight and obese young children and their parents. *J Pediatr Health Care*, *28*(3), 198-207. https://doi.org/10.1016/j.pedhc.2013.01.003

Smith, J. J., Morgan, P. J., Plotnikoff, R. C., Dally, K. A., Salmon, J., Okely, A. D., Finn, T. L., Babic, M. J., Skinner, G., & Lubans, D. R. (2014). Rationale and study protocol for the 'Active Teen Leaders Avoiding Screen-time' (ATLAS) group randomized controlled trial: An obesity prevention intervention for adolescent boys from schools in low-income communities [Article]. *Contemp Clin Trials*, *37*(1), 106-119. https://doi.org/10.1016/j.cct.2013.11.008

Smith, J. J., Morgan, P. J., Plotnikoff, R. C., Dally, K. A., Salmon, J., Okely, A. D., Finn, T. L., & Lubans, D. R. (2014). Smart-phone obesity prevention trial for adolescent boys in low-income communities: The ATLAS RCT [Article]. *Pediatrics*, *134*(3), e723-e731. https://doi.org/10.1542/peds.2014-1012

Smith, L. P., Hua, J., Seto, E., Du, S., Zang, J., Zou, S., Popkin, B. M., & Mendez, M. A. (2014). Development and validity of a 3-day smartphone assisted 24-hour recall to assess beverage consumption in a Chinese population: a randomized cross-over study [Article]. *Asia Pac J Clin Nutr*, *23*(4), 678-690. https://doi.org/10.6133/apjcn.2014.23.4.10

Sniehotta, F. F., Evans, E. H., Sainsbury, K., Adamson, A., Batterham, A., Becker, F., Brown, H., Dombrowski, S. U., Jackson, D., Howell, D., Ladha, K., McColl, E., Olivier, P., Rothman, A. J., Steel, A., Vale, L., Vieira, R., White, M., Wright, P., & Araújo-Soares, V. (2019). Behavioural intervention for weight loss maintenance versus standard weight advice in adults with obesity: A randomised controlled trial in the UK (NULevel trial) [Article]. *PLoS Med*, *16*(5). https://doi.org/10.1371/journal.pmed.1002793

Soureti, A., Murray, P., Cobain, M., Chinapaw, M., van Mechelen, W., & Hurling, R. (2011). Exploratory study of web-based planning and mobile text reminders in an overweight population. *J Med Internet Res*, *13*(4), e118. https://doi.org/10.2196/jmir.1773

Spadaro, K. C., Davis, K. K., Sereika, S. M., Gibbs, B. B., Jakicic, J. M., & Cohen, S. M. (2017). Effect of mindfulness meditation on short-term weight loss and eating behaviors in overweight and obese adults: A randomized controlled trial. *J Complement Integr Med*, *15*(2). https://doi.org/10.1515/jcim-2016-0048

Spring, B., Duncan, J. M., Janke, E. A., Kozak, A. T., McFadden, H. G., Demott, A., Pictor, A., Epstein, L. H., Siddique, J., Pellegrini, C. A., Buscemi, J., & Hedeker, D. (2013). Integrating technology into standard weight loss treatment a randomized controlled trial [Article]. *JAMA Intern Med*, *173*(2), 105-111. https://doi.org/10.1001/jamainternmed.2013.1221

Staiano, A. E., Beyl, R. A., Guan, W., Hendrick, C. A., Hsia, D. S., & Newton, R. L. (2018). Home-based exergaming among children with overweight and obesity: a randomized clinical trial [Article]. *Pediatr Obes*, *13*(11), 724-733. https://doi.org/10.1111/ijpo.12438

Steegers-Theunissen, R. P. M. (2018). Preconceptional personalised mHealth lifestyle coaching:\rFirst results of a randomized controlled trial in couples undergoing IVF/ICSI treatment [Conference Abstract]. *Human Reproduction*, *33*, i74-i75. https://doi.org/10.1093/humrep/33.Supplement_1.1

Stein, R. I., Strickland, J. R., Tabak, R. G., Dale, A. M., Colditz, G. A., & Evanoff, B. A. (2019). Design of a randomized trial testing a multi-level weight-control intervention to reduce obesity and related health conditions in low-income workers [Article]. *Contemp Clin Trials*, *79*, 89-97. https://doi.org/10.1016/j.cct.2019.01.011

Steinberg, D. M., Levine, E. L., Askew, S., Foley, P., & Bennett, G. G. (2013). Daily text messaging for weight control among racial and ethnic minority women: randomized controlled pilot study [Article]. *J Med Internet Res*, *15*(11), e244. http://www.embase.com/search/results?subaction=viewrecord&from=export&id=L563067773

Steinberg, D. M., Levine, E. L., Lane, I., Askew, S., Foley, P. B., Puleo, E., & Bennett, G. G. (2014). Adherence to self-monitoring via interactive voice response technology in an eHealth intervention targeting weight gain prevention among Black women: randomized controlled trial [Article]. *J Med Internet Res*, *16*(4), e114. https://doi.org/10.2196/jmir.2996

Steinberg, D. M., Tate, D. F., Bennett, G. G., Ennett, S., Samuel-Hodge, C., & Ward, D. S. (2013). The efficacy of a daily self-weighing weight loss intervention using smart scales and e-mail [Article]. *Obesity*, *21*(9), 1789-1797. https://doi.org/10.1002/oby.20396

Stewart, T., Beyl, R., Switzer, M., Friedl, K., Young, A., Ryan, D., & Williamson, D. (2017). HEALTH (Healthy Eating, Activity, Lifestyle Training Headquarters) internet/mobile weight management program for the U.S. Army: Outcomes and future directions [Conference Abstract]. *Journal of Science and Medicine in Sport*, *20*, S34-S35. https://doi.org/10.1016/j.jsams.2017.09.104

Stomby, A., Simonyte, K., Mellberg, C., Ryberg, M., Stimson, R. H., Larsson, C., Lindahl, B., Andrew, R., Walker, B. R., & Olsson, T. (2015). Diet-induced weight loss has chronic tissue-specific effects on glucocorticoid metabolism in overweight postmenopausal women. *Int J Obes (Lond)*, *39*(5), 814-819. https://doi.org/10.1038/ijo.2014.188

Stuart, K. L., Wyld, B., Bastiaans, K., Stocks, N., Brinkworth, G., Mohr, P., & Noakes, M. (2014). A telephone-supported cardiovascular lifestyle programme (CLIP) for lipid reduction and weight loss in general practice patients: a randomised controlled pilot trial. *Public Health Nutr*, *17*(3), 640-647. https://doi.org/10.1017/s1368980013000220

Stuckey, M. I., Shapiro, S., Gill, D. P., & Petrella, R. J. (2013). A lifestyle intervention supported by mobile health technologies to improve the cardiometabolic risk profile of individuals at risk for cardiovascular disease and type 2 diabetes: study rationale and protocol [Article]. *BMC Public Health*, *13*, 1051. https://doi.org/10.1186/1471-2458-13-1051

Stumm, G., Blaik, A., Kropf, S., Westphal, S., Hantke, T. K., & Luley, C. (2016). Long-Term Follow-Up of the Telemonitoring Weight-Reduction Program "Active Body Control" *J Diabetes Res*, *2016*, 3798729.

Sukumar, N., Dallosso, H., Saravanan, P., Yates, T., Telling, C., Shorthose, K., Northern, A., Schreder, S., Brough, C., Gray, L. J., Davies, M. J., & Khunti, K. (2018). Baby Steps - A structured group education programme with accompanying mobile web application designed to promote physical activity in women with a history of gestational diabetes: Study protocol for a randomised controlled trial [Article]. *Trials*, *19*(1). https://doi.org/10.1186/s13063-018-3067-8

Suso-Ribera, C., Mesas, A., Medel, J., Server, A., Márquez, E., Castilla, D., Zaragozá, I., & García-Palacios, A. (2018). Improving pain treatment with a smartphone app: Study protocol for a randomized controlled trial [Article]. *Trials*, *19*(1). https://doi.org/10.1186/s13063-018-2539-1

Sutherland, R., Brown, A., Nathan, N., Janssen, L., Reynolds, R., Walton, A., Hudson, N., Chooi, A., Yoong, S., Wiggers, J., Bailey, A., Evans, N., Gillham, K., Oldmeadow, C., Searles, A., Reeves, P., Rissel, C., Davies, M., Reilly, K., Cohen, B., McCallum, T., & Wolfenden, L. (2019). Protocol for an effectiveness- implementation hybrid trial to assess the effectiveness and cost-effectiveness of an m-health intervention to decrease the consumption of discretionary foods packed in school lunchboxes: the 'SWAP IT' trial [Article]. *BMC Public Health*, *19*(1), 1510. https://doi.org/10.1186/s12889-019-7725-x

Sutherland, R., Nathan, N., Brown, A., Yoong, S., Finch, M., Lecathelinais, C., Reynolds, R., Walton, A., Janssen, L., Desmet, C., Gillham, K., Herrmann, V., Hall, A., Wiggers, J., & Wolfenden, L. (2019). A randomized controlled trial to assess the potential efficacy, feasibility and acceptability of an m-health intervention targeting parents of school aged children to improve the nutritional quality of foods packed in the lunchbox 'SWAP IT' [Article]. *International Journal of Behavioral Nutrition and Physical Activity*, *16*(1). https://doi.org/10.1186/s12966-019-0812-7

Svetkey, L. P., Clark, J. M., Funk, K., Corsino, L., Batch, B. C., Hollis, J. F., Appel, L. J., Brantley, P. J., Loria, C. M., Champagne, C. M., Vollmer, W. M., & Stevens, V. J. (2014). Greater weight loss with increasing age in the weight loss maintenance trial. *Obesity (Silver Spring)*, *22*(1), 39-44. https://doi.org/10.1002/oby.20506

Sweeney, A. M., Wilson, D. K., Loncar, H., & Brown, A. (2019). Secondary benefits of the families improving together (FIT) for weight loss trial on cognitive and social factors in African American adolescents. *Int J Behav Nutr Phys Act*, *16*(1), 47. https://doi.org/10.1186/s12966-019-0806-5

Swencionis, C., Wylie-Rosett, J., Lent, M. R., Ginsberg, M., Cimino, C., Wassertheil-Smoller, S., Caban, A., & Segal-Isaacson, C. J. (2013). Weight change, psychological well-being, and vitality in adults participating in a cognitive-behavioral weight loss program. *Health Psychol*, *32*(4), 439-446. https://doi.org/10.1037/a0029186

Swift, D. L., Johannsen, N. M., Lavie, C. J., Earnest, C. P., Blair, S. N., & Church, T. S. (2016). Effects of clinically significant weight loss with exercise training on insulin resistance and cardiometabolic adaptations. *Obesity (Silver Spring)*, *24*(4), 812-819. https://doi.org/10.1002/oby.21404

Tanaka, M., Adachi, Y., Adachi, K., & Sato, C. (2010). Effects of a non-face-to-face behavioral weight-control program among Japanese overweight males: a randomized controlled trial *Int J Behav Med*, *17*(1), 17-24.

http://vp9py7xf3h.search.serialssolutions.com/?sid=OVID:medline&id=pmid:19685190&id=doi:10.1007%2Fs12529-009-9057-1&issn=1070-5503&isbn=&volume=17&issue=1&spage=17&pages=17-24&date=2010&title=International+Journal+of+Behavioral+Medicine&atitle=Effects+of+a+non-face-to-face+behavioral+weight-control+program+among+Japanese+overweight+males%3A+a+randomized+controlled+trial.&aulast=Tanaka&pid=%3Cauthor%3ETanaka+M%3BAdachi+Y%3BAdachi+K%3BSato+C%3C%2Fauthor%3E%3CAN%3E19685190%3C%2FAN%3E%3CDT%3EJournal+Article%3C%2FDT%3E

Tate, D. F., Valle, C. G., Crane, M. M., Nezami, B. T., Samuel-Hodge, C. D., Hatley, K. E., Diamond, M., & Polzien, K. (2017). Randomized trial comparing group size of periodic in-person sessions in a remotely delivered weight loss intervention. *Int J Behav Nutr Phys Act*, *14*(1), 144. https://doi.org/10.1186/s12966-017-0599-3

Tene, L., Shelef, I., Schwarzfuchs, D., Gepner, Y., Yaskolka Meir, A., Tsaban, G., Zelicha, H., Bilitzky, A., Komy, O., Cohen, N., Bril, N., Rein, M., Serfaty, D., Kenigsbuch, S., Chassidim, Y., Sarusy, B., Ceglarek, U., Stumvoll, M., Blüher, M., Thiery, J., Stampfer, M. J., Rudich, A., & Shai, I. (2018). The effect of long-term weight-loss intervention strategies on the dynamics of pancreatic-fat and morphology: An MRI RCT study. *Clin Nutr ESPEN*, *24*, 82-89. https://doi.org/10.1016/j.clnesp.2018.01.008

Teong, X. T., Liu, K., Hutchison, A. T., Liu, B., Feinle-Bisset, C., Wittert, G. A., Lange, K., Vincent, A. D., & Heilbronn, L. K. (2020). Rationale and protocol for a randomized controlled trial comparing daily calorie restriction versus intermittent fasting to improve glycaemia in individuals at increased risk of developing type 2 diabetes [Article in Press]. *Obesity Research and Clinical Practice*. https://doi.org/10.1016/j.orcp.2020.01.005

Theissing, J., Deck, R., & Raspe, H. (2013). [Liveonline aftercare in patients with abdominal obesity in cardio-diabetological rehabilitation: findings of a randomized controlled study]. *Rehabilitation (Stuttg)*, *52*(3), 153-154. https://doi.org/10.1055/s-0033-1345190 (Liveonline-Nachbetreuung bei Patienten mit abdominaler Adipositas in der kardio-diabetologischen Rehabilitation: Ergebnisse einer randomisierten, kontrollierten Studie.)

Thomas, J. G., Raynor, H. A., Bond, D. S., Luke, A. K., Cardoso, C. C., Foster, G. D., & Wing, R. R. (2017). Weight loss in Weight Watchers Online with and without an activity tracking device compared to control: A randomized trial [Randomized Controlled Trial]. *Obesity*, *25*(6), 1014-1021. http://ovidsp.ovid.com/ovidweb.cgi?T=JS&CSC=Y&NEWS=N&PAGE=fulltext&D=med14&AN=28437597

http://vp9py7xf3h.search.serialssolutions.com/?sid=OVID:medline&id=pmid:28437597&id=doi:10.1002%2Foby.21846&issn=1930-7381&isbn=&volume=25&issue=6&spage=1014&pages=1014-1021&date=2017&title=Obesity&atitle=Weight+loss+in+Weight+Watchers+Online+with+and+without+an+activity+tracking+device+compared+to+control%3A+A+randomized+trial.&aulast=Thomas&pid=%3Cauthor%3EThomas+JG%3BRaynor+HA%3BBond+DS%3BLuke+AK%3BCardoso+CC%3BFoster+GD%3BWing+RR%3C%2Fauthor%3E%3CAN%3E28437597%3C%2FAN%3E%3CDT%3EJournal+Article%3C%2FDT%3E

Timpel, P., Cesena, F. H. Y., da Silva Costa, C., Soldatelli, M. D., Gois, E., Castrillon, E., Díaz, L. J. J., Repetto, G. M., Hagos, F., Castillo Yermenos, R. E., Pacheco-Barrios, K., Musallam, W., Braid, Z., Khidir, N., Romo Guardado, M., & Roepke, R. M. L. (2018). Efficacy of gamification-based smartphone application for weight loss in overweight and obese adolescents: study protocol for a phase II randomized controlled trial [Article]. *Therapeutic Advances in Endocrinology and Metabolism*, *9*(6), 167-176. https://doi.org/10.1177/2042018818770938

Trief, P. M., Fisher, L., Sandberg, J., Cibula, D. A., Dimmock, J., Hessler, D. M., Forken, P., & Weinstock, R. S. (2016). Health and psychosocial outcomes of a telephonic couples behavior change intervention in patients with poorly controlled type 2 diabetes: A randomized clinical trial [Article]. *Diabetes Care*, *39*(12), 2165-2173. https://doi.org/10.2337/dc16-0035

Truby, H., Edwards, B. A., O'Driscoll, D. M., Young, A., Ghazi, L., Bristow, C., Roem, K., Bonham, M. P., Murgia, C., Day, K., Haines, T. P., & Hamilton, G. S. (2019). Sleeping Well Trial: Increasing the effectiveness of treatment with continuous positive airway pressure using a weight management program in overweight adults with obstructive sleep apnoea-A stepped wedge randomised trial protocol [Article]. *Nutrition & dietetics: the journal of the Dietitians Association of Australia*, *76*(1), 110-117. https://doi.org/10.1111/1747-0080.12435

Turner-McGrievy, G. M., Beets, M. W., Moore, J. B., Kaczynski, A. T., Barr-Anderson, D. J., & Tate, D. F. (2013). Comparison of traditional versus mobile app self-monitoring of physical activity and dietary intake among overweight adults participating in an mHealth weight loss program

Turner-McGrievy, G. M., Davidson, C. R., & Wilcox, S. (2014). Does the type of weight loss diet affect who participates in a behavioral weight loss intervention? A comparison of participants for a plant-based diet versus a standard diet trial [Article]. *Appetite*, *73*, 156-162. https://doi.org/10.1016/j.appet.2013.11.008

Turner-McGrievy, G. M., & Tate, D. F. (2013). Weight loss social support in 140 characters or less: Use of an online social network in a remotely delivered weight loss intervention [Article]. *Transl Behav Med*, *3*(3), 287-294. https://doi.org/10.1007/s13142-012-0183-y

Turner-McGrievy, G. M., & Tate, D. F. (2014). Are we sure that Mobile Health is really mobile? An examination of mobile device use during two remotely-delivered weight loss interventions [Article]. *Int J Med Inform*, *83*(5), 313-319. https://doi.org/10.1016/j.ijmedinf.2014.01.002

Tyson, C. C., Appel, L. J., Vollmer, W. M., Jerome, G. J., Brantley, P. J., Hollis, J. F., Stevens, V. J., Ard, J. D., Patel, U. D., & Svetkey, L. P. (2013). Impact of 5-year weight change on blood pressure: results from the Weight Loss Maintenance trial. *J Clin Hypertens (Greenwich)*, *15*(7), 458-464. https://doi.org/10.1111/jch.12108

Unick, J. L., Hogan, P. E., Neiberg, R. H., Cheskin, L. J., Dutton, G. R., Evans-Hudnall, G., Jeffery, R., Kitabchi, A. E., Nelson, J. A., Pi-Sunyer, F. X., West, D. S., & Wing, R. R. (2014). Evaluation of early weight loss thresholds for identifying nonresponders to an intensive lifestyle intervention. *Obesity (Silver Spring)*, *22*(7), 1608-1616. https://doi.org/10.1002/oby.20777

van Grieken, A., Vlasblom, E., Wang, L., Beltman, M., Boere-Boonekamp, M. M., L'Hoir, M. P., & Raat, H. (2017). Personalized Web-Based Advice in Combination With Well-Child Visits to Prevent Overweight in Young Children: Cluster Randomized Controlled Trial. *J Med Internet Res*, *19*(7), e268. https://doi.org/10.2196/jmir.7115

Van Horn, L., Peaceman, A., Kwasny, M., Vincent, E., Fought, A., Josefson, J., Spring, B., Neff, L. M., & Gernhofer, N. (2018). Dietary Approaches to Stop Hypertension Diet and Activity to Limit Gestational Weight: Maternal Offspring Metabolics Family Intervention Trial, a Technology Enhanced Randomized Trial [Article]. *Am J Prev Med*, *55*(5), 603-614. https://doi.org/10.1016/j.amepre.2018.06.015

VanWormer, J. J., Martinez, A. M., Cosentino, D., & Pronk, N. P. (2010). Satisfaction with a weight loss program: what matters? *Am J Health Promot*, *24*(4), 238-245. https://doi.org/10.4278/ajhp.080613-QUAN-92

Varnfield, M., Alcorn, J., Parker, S., & Karunanithi, M. (2016). Translation of home-based m-health cardiac rehabilitation in real practice [Conference Abstract]. *European Heart Journal*, *37*, 826-827. https://doi.org/10.1093/eurheartj/ehw433

Varnfield, M., Karunanithi, M., Lee, C. K., Honeyman, E., Arnold, D., Ding, H., Smith, C., & Walters, D. L. (2014). Smartphone-based home care model improved use of cardiac rehabilitation in postmyocardial infarction patients: Results from a randomised controlled trial [Article in Press]. *Heart*. https://doi.org/10.1136/heartjnl-2014-305783

Ventura Marra, M., Lilly, C. L., Nelson, K. R., Woofter, D. R., & Malone, J. (2019). A Pilot Randomized Controlled Trial of a Telenutrition Weight Loss Intervention in Middle-Aged and Older Men with Multiple Risk Factors for Cardiovascular Disease. *Nutrients*, *11*(2). https://doi.org/10.3390/nu11020229

Versteegden, D., Scheerhoorn, J., Berghuis, K., Said, M., Aarts, M., Van Hout, G., & Nienhuijs, S. (2019). The value of an ehealth platform in bariatrics: A retrospective study [Conference Abstract]. *Obes Surg*, *29*(5), 389. https://doi.org/10.1007/s11695-019-04101-1

Versteegden, D., Van Himbeeck, M., Jf, S., De Zoete, J., Van Montfort, G., & Nienhuijs, S. (2019). A randomized controlled trial for assessing the value of ehealth in bariatric care: 1-year-bepatient trial results [Conference Abstract]. *Obes Surg*, *29*(5), 776. https://doi.org/10.1007/s11695-019-04101-1

Versteegden, D. P. A., Van Himbeeck, M. J. J., & Nienhuijs, S. W. (2018). Assessing the value of eHealth for bariatric surgery (BePatient trial): Study protocol for a randomized controlled trial [Article]. *Trials*, *19*(1). https://doi.org/10.1186/s13063-018-3020-x

Vidmar, A. P., Salvy, S. J., Pretlow, R., Mittelman, S. D., Wee, C. P., Fink, C., Steven Fox, D., & Raymond, J. K. (2019). An addiction-based mobile health weight loss intervention: protocol of a randomized controlled trial [Article]. *Contemp Clin Trials*, *78*, 11-19. https://doi.org/10.1016/j.cct.2019.01.008

Vinter, C. A., Jensen, D. M., Ovesen, P., Beck-Nielsen, H., Tanvig, M., Lamont, R. F., & Jørgensen, J. S. (2014). Postpartum weight retention and breastfeeding among obese women from the randomized controlled Lifestyle in Pregnancy (LiP) trial. *Acta Obstet Gynecol Scand*, *93*(8), 794-801. https://doi.org/10.1111/aogs.12429

Voils, C. I., Levine, E., Gierisch, J. M., Pendergast, J., Hale, S. L., McVay, M. A., Reed, S. D., Yancy, W. S., Bennett, G., Strawbridge, E. M., White, A. C., & Shaw, R. J. (2018). Study protocol for Log2Lose: A feasibility randomized controlled trial to evaluate financial incentives for dietary self-monitoring and interim weight loss in adults with obesity [Article]. *Contemp Clin Trials*, *65*, 116-122. https://doi.org/10.1016/j.cct.2017.12.007

Voils, C. I., Olsen, M. K., Gierisch, J. M., McVay, M. A., Grubber, J. M., Gaillard, L., Bolton, J., Maciejewski, M. L., Strawbridge, E., & Yancy, W. S., Jr. (2017). Maintenance of Weight Loss After Initiation of Nutrition Training: A Randomized Trial. *Ann Intern Med*, *166*(7), 463-471. https://doi.org/10.7326/m16-2160

Volkova, E., Neal, B., Rayner, M., Swinburn, B., Eyles, H., Jiang, Y., Michie, J., & Mhurchu, C. N. (2014). Effects of interpretive front-of-pack nutrition labels on food purchases: Starlight randomised controlled trial [Conference Abstract]. *Obesity Research and Clinical Practice*, *8*, 110-111. https://doi.org/org/10.1016/j.orcp.2014.10.199

Wadden, T. A., Neiberg, R. H., Wing, R. R., Clark, J. M., Delahanty, L. M., Hill, J. O., Krakoff, J., Otto, A., Ryan, D. H., & Vitolins, M. Z. (2011). Four-year weight losses in the Look AHEAD study: factors associated with long-term success. *Obesity (Silver Spring)*, *19*(10), 1987-1998. https://doi.org/10.1038/oby.2011.230

Wagner, B., Nagl, M., Dölemeyer, R., Klinitzke, G., Steinig, J., Hilbert, A., & Kersting, A. (2016). Randomized Controlled Trial of an Internet-Based Cognitive-Behavioral Treatment Program for Binge-Eating Disorder [Article]. *Behav Ther*, *47*(4), 500-514. https://doi.org/10.1016/j.beth.2016.01.006

Wang, Y., Lombard, C., Hussain, S. M., Harrison, C., Kozica, S., Brady, S. R. E., Teede, H., & Cicuttini, F. M. (2018). Effect of a low-intensity, self-management lifestyle intervention on knee pain in community-based young to middle-aged rural women: a cluster randomised controlled trial [Randomized Controlled Trial

Warschburger, P., & Zitzmann, J. (2019). Does an Age-Specific Treatment Program Augment the Efficacy of a Cognitive-Behavioral Weight Loss Program in Adolescence and Young Adulthood? Results from a Controlled Study. *Nutrients*, *11*(9). https://doi.org/10.3390/nu11092053

Webber, K. H., & Rose, S. A. (2013). A pilot Internet-based behavioral weight loss intervention with or without commercially available portion-controlled foods. *Obesity (Silver Spring)*, *21*(9), E354-359. https://doi.org/10.1002/oby.20331

Weinstock, R. S., Brooks, G., Palmas, W., Morin, P. C., Teresi, J. A., Eimicke, J. P., Silver, S., Izquierdo, R., Goland, R., & Shea, S. (2011). Lessened decline in physical activity and impairment of older adults with diabetes with telemedicine and pedometer use: results from the IDEATel study [Randomized Controlled Trial

Werk, L. N., Hossain, J., Martinez, A., Abatemarco, A., Carlo, V., Barnini, N., McCahan, S., Pennington, C., Phan, T. L., Bunnell, T., & Hassink, S. G. (2019). Extending obesity care beyond the office doors using telemedicine health coaches. A pilot test randomized trial [Conference Abstract]. *Pediatrics*, *144*(2). https://doi.org/10.1542/peds.144.2-MeetingAbstract.219

West, D. S., Bursac, Z., Cornell, C. E., Felix, H. C., Fausett, J. K., Krukowski, R. A., Lensing, S., Love, S. J., Prewitt, T. E., & Beck, C. (2011). Lay health educators translate a weight-loss intervention in senior centers: a randomized controlled trial. *Am J Prev Med*, *41*(4), 385-391. https://doi.org/10.1016/j.amepre.2011.06.041

West, D. S., Stansbury, M., Krukowski, R. A., & Harvey, J. (2019). Enhancing group-based internet obesity treatment: A pilot RCT comparing video and text-based chat [Article]. *Obesity Science and Practice*, *5*(6), 513-520. https://doi.org/10.1002/osp4.371

Whittemore, R., Vilar-Compte, M., De La Cerda, S., Delvy, R., Jeon, S., Burrola-Méndez, S. I., Lozano-Marrufo, A. A., & Perez-Escamilla, R. (2019). Yo puedo! A self-management group and mHealth program for low-income adults with type 2 diabetes in Mexico City [Conference Abstract]. *Diabetes*, *68*. https://doi.org/10.2337/db19-339-OR

Widmer, R. J., Allison, T. G., Lennon, R., Lopez-Jimenez, F., Lerman, L. O., & Lerman, A. (2017). Digital health intervention during cardiac rehabilitation: A randomized controlled trial [Article]. *American Heart Journal*, *188*, 65-72. https://doi.org/10.1016/j.ahj.2017.02.016

Wilczynska, M., Lubans, D. R., Cohen, K. E., Smith, J. J., Robards, S. L., & Plotnikoff, R. C. (2016). Rationale and study protocol for the 'eCoFit' randomized controlled trial: Integrating smartphone technology, social support and the outdoor physical environment to improve health-related fitness among adults at risk of, or diagnosed with, Type 2 Diabetes [Randomized Controlled Trial]. *Contemp Clin Trials*, *49*, 116-125. http://ovidsp.ovid.com/ovidweb.cgi?T=JS&CSC=Y&NEWS=N&PAGE=fulltext&D=med13&AN=27370229

Willcox, J. C., Campbell, K. J., McCarthy, E. A., Wilkinson, S. A., Lappas, M., Ball, K., Fjeldsoe, B., Griffiths, A., Whittaker, R., Maddison, R., Shub, A., Pidd, D., Fraser, E., Moshonas, N., & Crawford, D. A. (2015). Testing the feasibility of a mobile technology intervention promoting healthy gestational weight gain in pregnant women (txt4two) - study protocol for a randomised controlled trial [Randomized Controlled Trial

Research Support, Non-U.S. Gov't]. *Trials [Electronic Resource]*, *16*, 209. http://ovidsp.ovid.com/ovidweb.cgi?T=JS&CSC=Y&NEWS=N&PAGE=fulltext&D=med12&AN=25947578

http://vp9py7xf3h.search.serialssolutions.com/?sid=OVID:medline&id=pmid:25947578&id=doi:10.1186%2Fs13063-015-0730-1&issn=1745-6215&isbn=&volume=16&issue=&spage=209&pages=209&date=2015&title=Trials+%5BElectronic+Resource%5D&atitle=Testing+the+feasibility+of+a+mobile+technology+intervention+promoting+healthy+gestational+weight+gain+in+pregnant+women+%28txt4two%29+-+study+protocol+for+a+randomised+controlled+trial.&aulast=Willcox&pid=%3Cauthor%3EWillcox+JC%3BCampbell+KJ%3BMcCarthy+EA%3BWilkinson+SA%3BLappas+M%3BBall+K%3BFjeldsoe+B%3BGriffiths+A%3BWhittaker+R%3BMaddison+R%3BShub+A%3BPidd+D%3BFraser+E%3BMoshonas+N%3BCrawford+DA%3C%2Fauthor%3E%3CAN%3E25947578%3C%2FAN%3E%3CDT%3EJournal+Article%3C%2FDT%3E

Willcox, J. C., Wilkinson, S. A., Lappas, M., Ball, K., Crawford, D., McCarthy, E. A., Fjeldsoe, B., Whittaker, R., Maddison, R., & Campbell, K. J. (2017). A mobile health intervention promoting healthy gestational weight gain for women entering pregnancy at a high body mass index: the txt4two pilot randomised controlled trial [Article]. *BJOG: An International Journal of Obstetrics and Gynaecology*, *124*(11), 1718-1728. https://doi.org/10.1111/1471-0528.14552

Williams, A., Wiggers, J., O'Brien, K. M., Wolfenden, L., Yoong, S. L., Hodder, R. K., Lee, H., Robson, E. K., McAuley, J. H., Haskins, R., Kamper, S. J., Risseli, C., & Williams, C. M. (2018). Effectiveness of a healthy lifestyle intervention for chronic low back pain: A randomised controlled trial [Article]. *Pain*, *159*(6), 1137-1146. https://doi.org/10.1097/j.pain.0000000000001198

Wipfli, B., Hanson, G., Anger, K., Elliot, D. L., Bodner, T., Stevens, V., & Olson, R. (2019). Process Evaluation of a Mobile Weight Loss Intervention for Truck Drivers [Article]. *Safety and Health at Work*, *10*(1), 95-102. https://doi.org/10.1016/j.shaw.2018.08.002

Wister, A., Loewen, N., Kennedy-Symonds, H., McGowan, B., McCoy, B., & Singer, J. (2007). One-year follow-up of a therapeutic lifestyle intervention targeting cardiovascular disease risk [Randomized Controlled Trial

Research Support, Non-U.S. Gov't]. *CMAJ Canadian Medical Association Journal*, *177*(8), 859-865. http://ovidsp.ovid.com/ovidweb.cgi?T=JS&CSC=Y&NEWS=N&PAGE=fulltext&D=med6&AN=17923653

Wong, E. M. L., Leung, D. Y. P., Wang, Q., & Leung, A. Y. M. (2020). A nurse-led lifestyle intervention using mobile application versus booklet for adults with metabolic syndrome-Protocol for a randomized controlled trial [Article]. *Journal of advanced nursing*, *76*(1), 364-372. https://doi.org/10.1111/jan.14241

Wongrochananan, S., Jiamjarasrangsi, W., Tuicomepee, A., & Buranarach, M. (2013). The effectiveness of interactive multi-modality intervention on self-management support of type 2 diabetic patients in Thailand: A clusterrandomized controlled trial [Conference Abstract]. *Journal of Diabetes*, *5*, 151-152. https://doi.org/10.1111/1753-0407.12032

Wu, L., Forbes, A., & While, A. (2010). Patients' experience of a telephone booster intervention to support weight management in Type 2 diabetes and its acceptability. *J Telemed Telecare*, *16*(4), 221-223. https://doi.org/10.1258/jtt.2010.004016

Xu, X., Leahey, T. M., Boguszewski, K., Krupel, K., Mailloux, K. A., & Wing, R. R. (2017). Self-Expansion is Associated with Better Adherence and Obesity Treatment Outcomes in Adults. *Ann Behav Med*, *51*(1), 13-17. https://doi.org/10.1007/s12160-016-9823-7

Yancy, W. S., Jr., Shaw, P. A., Wesby, L., Hilbert, V., Yang, L., Zhu, J., Troxel, A., Huffman, D., Foster, G. D., Wojtanowski, A. C., & Volpp, K. G. (2018). Financial incentive strategies for maintenance of weight loss: results from an internet-based randomized controlled trial. *Nutr Diabetes*, *8*(1), 33. https://doi.org/10.1038/s41387-018-0036-y

Yang, Z., Yu, Z., Jiang, Y., Bai, Y., Miller-Kovach, K., Zhao, W., Foster, G. D., & Chen, C. (2016). Evaluation of a community-based behavioral weight loss program in Chinese adults: A randomized controlled trial. *Obesity (Silver Spring)*, *24*(7), 1464-1470. https://doi.org/10.1002/oby.21527

Yank, V., Stafford, R. S., Rosas, L. G., & Ma, J. (2013). Baseline reach and adoption characteristics in a randomized controlled trial of two weight loss interventions translated into primary care: a structured report of real-world applicability. *Contemp Clin Trials*, *34*(1), 126-135. https://doi.org/10.1016/j.cct.2012.10.007

Yank, V., Xiao, L., Wilson, S. R., Stafford, R. S., Rosas, L. G., & Ma, J. (2014). Short-term weight loss patterns, baseline predictors, and longer-term follow-up within a randomized controlled trial. *Obesity (Silver Spring)*, *22*(1), 45-51. https://doi.org/10.1002/oby.20510

Yoo, H. J., Park, M. S., Kim, T. N., Yang, S. J., Cho, G. J., Hwang, T. G., Baik, S. H., Choi, D. S., Park, G. H., & Choi, K. M. (2009). A Ubiquitous Chronic Disease Care system using cellular phones and the internet [Article]. *Diabetic Medicine*, *26*(6), 628-635. https://doi.org/10.1111/j.1464-5491.2009.02732.x

Young, M. D., Callister, R., Collins, C. E., Plotnikoff, R. C., Aguiar, E. J., & Morgan, P. J. (2017). Efficacy of a gender-tailored intervention to prevent weight regain in men over 3 years: A weight loss maintenance RCT. *Obesity (Silver Spring)*, *25*(1), 56-65. https://doi.org/10.1002/oby.21696

Zang, J., Song, J., Wang, Z., Yao, C., Ma, J., Huang, C., Zhu, Z., Smith, L. P., Du, S., Hua, J., Seto, E., Popkin, B. M., & Zou, S. (2015). Acceptability and feasibility of smartphone-assisted 24 h recalls in the Chinese population [Article]. *Public Health Nutr*, *18*(18), 3272-3277. https://doi.org/10.1017/S1368980015000907

Zwickert, K., Rieger, E., Swinbourne, J., Manns, C., McAulay, C., Gibson, A. A., Sainsbury, A., & Caterson, I. D. (2016). High or low intensity text-messaging combined with group treatment equally promote weight loss maintenance in obese adults [Comparative Study

Randomized Controlled Trial]. *Obes Res Clin Pract*, *10*(6), 680-691. http://ovidsp.ovid.com/ovidweb.cgi?T=JS&CSC=Y&NEWS=N&PAGE=fulltext&D=med13&AN=26992569
