# Supplementary material for: The Effectiveness of Combining Nonmobile Interventions With the Use of Smartphone Apps With Various Features for Weight Loss: Systematic Review and Meta-analysis
Source: JMIR Mhealth Uhealth. 2022 Apr 8;10(4):e35479. doi: 10.2196/35479 (PMC9034427; doi:10.2196/35479)

Appendix 5: Funnel Plots

Funnel Plot for 3 months with at least one app


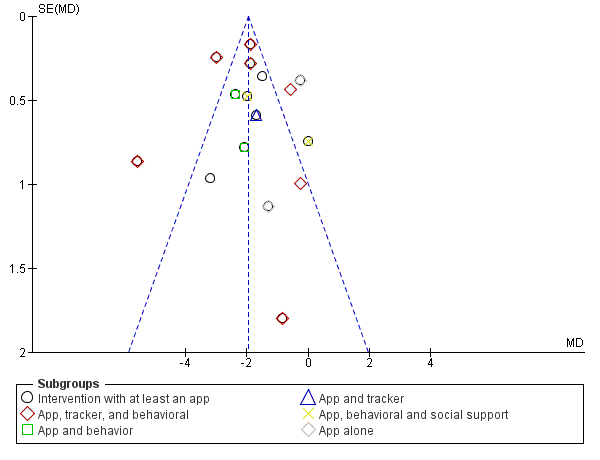


Funnel Plot for 6 months with at least one app


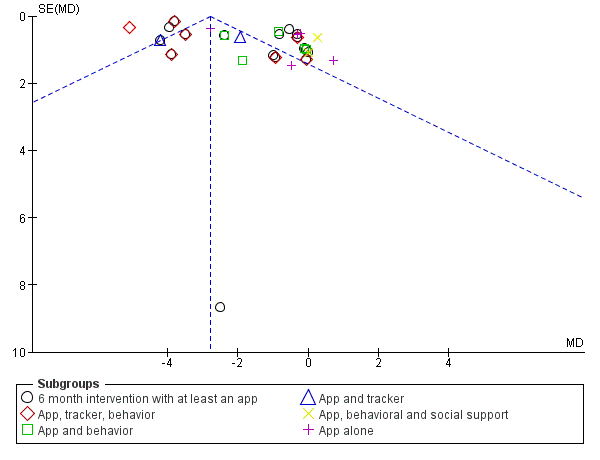

Supplement: Multimedia Appendix 3 [file mhealth_v10i4e35479_app3.docx]
