# Supplementary material for: The Effectiveness of Combining Nonmobile Interventions With the Use of Smartphone Apps With Various Features for Weight Loss: Systematic Review and Meta-analysis
Source: JMIR Mhealth Uhealth. 2022 Apr 8;10(4):e35479. doi: 10.2196/35479 (PMC9034427; doi:10.2196/35479)
Supplement: Multimedia Appendix 4 [file mhealth_v10i4e35479_app4.docx]

Appendix 3: Summary of the components of the intervention and control arms of the included studies.

|  |  | Interventions descriptions | | | | | | |
| --- | --- | --- | --- | --- | --- | --- | --- | --- |
|  | First author (year) | Mobile App | Trackers | Social support | Behavioral therapy/advice | Feedback | Meal replacement | Financial incentives |
| 1 | Bender (2017) | + | + | + | + |  |  |  |
| 2 | Fukuoka (2015) | + | + |  | + |  |  |  |
| 3 | Whitelock (2019) | + |  |  | + |  |  |  |
| 4 | Vaz (2018) | + | + |  |  |  |  |  |
| 5 | Thompson Felty (2017) | + |  |  |  |  |  |  |
| 6 |  | + |  |  |  |  |  |  |
| 7 |  | + |  |  |  | + |  |  |
| 8 | Rogers (2016) | + | + |  | + |  |  |  |
| 9 | Svetky (2015) | + | + |  |  |  |  |  |
| 10 |  | + | + | + | + |  |  |  |
| 11 | Thomas (2019) | + |  |  | + | + |  |  |
| 12 | Brindal (2013) | + |  |  |  |  | + |  |
| 13 | Laing (2014) | + |  |  |  |  |  |  |
| 14 | Spring (2017) | + | + |  | + | + |  |  |
| 15 | Shin (2017) | + | + |  | + |  |  |  |
| 16 |  | + | + |  | + |  |  |  |
| 17 | Ross (2016) | + | + |  | + |  |  |  |
| 18 |  | + | + |  | + |  |  |  |
| 19 | Gilmore (2017) | + | + |  |  |  |  |  |
| 20 | Tanaka (2018) | + |  |  |  |  |  |  |
| 21 | Allen (2013) | + |  |  |  |  |  |  |
| 22 |  | + |  |  | + |  |  |  |
| 23 |  | + |  |  | + |  |  |  |
| 24 | Stephens (2017) | + |  |  | + | + |  |  |
| 25 | Hales (2016) | + |  |  | + |  |  |  |
| 26 |  | + |  |  | + |  |  |  |
| 27 | Hartman (2016) | + | + |  | + |  |  |  |
| 28 | Haufe (2019) | + | + |  | + |  |  |  |
| 29 | Turner (2017) | + | + |  | + |  |  |  |
| 30 | Jospe (2017) | + |  |  |  |  |  |  |
| 31 | Burke (2017) | + |  |  |  |  |  |  |
| 32 |  | + |  | + | + |  |  |  |
| 33 |  | + |  | + |  |  |  |  |
| 34 | Lee (2019) | + |  |  |  |  |  |  |
| 35 |  | + |  |  | + |  |  |  |
| 36 | Tuner (2011) | + |  | + | + |  |  |  |
| 37 | Monroe (2019) | + | + | + | + | + |  |  |
| 38 |  | + | + |  | + | + |  |  |
| 39 | Choi (2019) | + |  |  | + |  |  |  |
| 40 | Evangelista (2018) | + | + |  | + | + |  |  |
| 41 |  | + | + |  | + |  |  |  |
| 42 | Kurtzman (2018) | + | + | + |  | + |  | + |
| 43 |  | + | + | + |  |  |  | + |
| 44 |  | + | + | + |  |  |  |  |
| 45 | Carter (2013) | + |  | + |  |  |  |  |
| 46 | Duncan (2020) | + | + |  | + |  |  |  |
| 47 |  | + | + |  | + |  |  |  |
| 48 | Lim (2021) | + |  |  | + |  |  |  |
| 49 | Ahn (2020) | + |  |  |  |  |  |  |
| 50 | Lugones-Sanchez (2020) | + | + |  | + |  |  |  |

|  |  | Control description | | | | | | | |
| --- | --- | --- | --- | --- | --- | --- | --- | --- | --- |
|  | First author  (year) | Trackers | Behavioral therapy/advice | Feedback | Self-monitoring | Social support | Meal replacement | Financial incentives | Usual care/waitlist |
| 1 | Bender (2017) | + |  |  |  |  |  |  | + |
| 2 | Fukuoka (2015) | + | + |  |  |  |  |  |  |
| 3 | Whitelock (2019) |  | + |  |  |  |  |  |  |
| 4 | Vaz ( 2018) |  | + |  |  |  |  |  |  |
| 5 | Rogers (2016) | + | + |  | + |  |  |  |  |
| 6 |  |  | + | + | + |  |  |  |  |
| 7 | Svetky (2015) |  | + |  |  |  |  |  |  |
| 8 | Thomas (2019) |  | + | + | + |  |  |  |  |
| 9 |  |  | + | + | + |  |  |  |  |
| 10 | Brindal (2013) |  |  |  |  |  | + |  |  |
| 11 | Laing (2014) |  |  |  |  |  |  |  | + |
| 12 | Spring (2017) |  | + | + | + |  |  |  |  |
| 13 |  |  | + |  | + |  |  |  |  |
| 14 | Shin (2017) |  | + |  |  |  |  |  |  |
| 15 | Ross (2016) | + | + |  | + |  |  |  |  |
| 16 | Gilmore (2017) |  |  |  |  |  |  |  | + |
| 17 | Tanaka ( 2018) |  |  |  |  |  |  |  | + |
| 18 | Allen (2013) |  | + |  |  |  |  |  |  |
| 19 | Stephens (2017) |  | + |  |  |  |  |  |  |
| 20 | Hartman (2016) |  | + |  |  |  |  |  |  |
| 21 | Haufe (2019) |  |  |  |  |  |  |  | + |
| 22 | Turner (2017) | + | + |  |  |  |  |  |  |
| 23 | Jospe (2017) |  |  |  | + | + |  |  |  |
| 24 |  |  | + |  | + |  |  |  |  |
| 25 |  |  | + |  |  |  |  |  |  |
| 26 |  |  | + |  |  |  |  |  |  |
| 27 | Lee ( 2019) |  |  |  |  |  |  |  | + |
| 28 | Tuner ( 2011) |  | + |  | + |  |  |  |  |
| 29 | Monroe (2019) |  |  |  | + |  |  |  |  |
| 30 | Choi ( 2019) |  | + |  |  |  |  |  |  |
| 31 | Carter (2013) |  |  |  | + | + |  |  |  |
| 32 | Duncan (2020) |  |  |  |  |  |  |  | + |
| 33 | Lim (2021) |  |  |  |  |  |  |  | + |
| 34 | Ahn (2020) |  |  |  |  |  |  |  |  |
| 35 | Lugones-Sanchez (2020) |  | + |  |  |  |  |  |  |
