# Supplementary material for: The Effectiveness of Combining Nonmobile Interventions With the Use of Smartphone Apps With Various Features for Weight Loss: Systematic Review and Meta-analysis
Source: JMIR Mhealth Uhealth. 2022 Apr 8;10(4):e35479. doi: 10.2196/35479 (PMC9034427; doi:10.2196/35479)
Supplement: Multimedia Appendix 5 [file mhealth_v10i4e35479_app5.docx]

Appendix 4: Summary of the adherence measures

| **Author,**  **Reference**  **country** | **Adherence themes** | **Definition of adherence** |
| --- | --- | --- |
| Bender (2017)  USA | Weight self-monitoring | Logging weight at least once per week |
|  | Dietary self-monitoring | Logging food/calorie intake at least once per week |
|  | Interaction with app | Wearing the Fitbit at least 5 days per week |
| Fukuoka (2015)  USA | Interaction with app | Wearing the pedometer for least 8 hours per day and 4 days per week. |
| Whitelock (2019)  USA | Interaction with app | Accessing the application.  Completing entries immediately after taking the meal  photograph with fewer than 0.5 gallery views per day. |
|  | Dietary self-monitoring | Recording 4 diary entries per day on at least half of the trial days. |
| Thompson felty (2017)  USA | Dietary self-monitoring | Number of days that the app was used for diet tracking divided by 56 |
| Roger (2016)  USA | Dietary self-monitoring | The number of days any intake was recorded. |
|  | Physical activity self-monitoring | The number of days any activity was recorded. |
| SvetkEy (2015)  USA | Weight self-monitoring | The mean time of self-weighing per week |
|  | Interaction with the app | Number of interactions with apps components in time per person per day. |
| Mameli (2018)  Italy | Interaction with the app | Wearing the WristBand from awakening to bedtime and using the APP for at least 5 days per week. |
| Thomas (2019)  USA | Weight self-monitoring | Rates of weight self-monitoring in days |
|  | Dietary self-monitoring | Recording either three or more separate eating events or intake equaling 50% or more of the calorie goal for the day. |
| Brindal (2013)  Australia | Interaction with the app | Assessing the number of prompts sent and  completed prior to the subsequent prompt |
|  | Weight self-monitoring | The number of response to morning prompts to enter bodyweight |
|  | Dietary self-monitoring | The number Completed prompts asking to enter  meals prior to the evening prompt |
| Laing (2014)  USA | Interaction with the app | Frequency of app logins over time (Each time a participant opened the app counted as a  “login). |
| Spring (2017)  USA | Dietary self-monitoring | Percent of days reporting energy intake of >1,000 calories |
|  | Physical activity self-monitoring | Percent of days when any physical activity was detected on the accelerometer |
|  | Weight self-monitoring | Percent of days when a body weight was recorded |
| Ross (2016)  USA | Dietary self-monitoring | The number of days that participants completed food records through the Fitbit app or website |
|  | Weight self-monitoring | The number of days that participants used smartscale to track their weight |
|  | Physical activity self-monitoring | The number of days participants wore the Fitbit to track activity |
| Allen (2013)  USA | Interaction with the app | Ratio of the number of counseling sessions or actual days of logging relative to the possible number of sessions or days |
| Stephens (2017)  USA | Dietary self-monitoring | Number of days of logging in the dietary intake |
|  | Physical activity self-monitoring | Number of days of logging in the physical activity |
| Hales (2016)  USA | Interaction with the app | Reporting the total number of days that they used their tracking apps on weekly basis. |
| Turner (2017)  USA | Dietary self-monitoring | Number of days diet was tracked: tracked any food or beverage on their app for the day or logging any bites with their Bite Counter |
| Jospe (2017)  Newzeland | Dietary self-monitoring | Percentage of days of entering food intake, divided by the recommended number of days, yielding an adherence score from 0 to 100 |
|  | Weight self-monitoring | Percentage of days ofself weighing, divided by the recommended number of days, yielding an adherence score from 0 to 100 |
| Burke (2017)  USA | Dietary self- monitoring | Percentage of days recording ≥ 50% of the prescribed daily calorie intake goal |
| Turner (2011)  USA | Dietary self-monitoring | Number of days users monitored their diet (mean days per week) |
|  | Physical activity self-monitoring | Number of days users monitored their physical activity (mean days per week) |
| Nystrom (2019)  Sweden | Interaction with the app | Number of feedback messages read (max 140) |
|  | Dietary self- monitoring | Number of recordings for fruits, candy, sweetened beverages, and sedentary behavior (max 168) |
| Choi Monroe (2019)  USA | Dietary self-monitoring | Total number of days that participants logged two meals or snacks or more on a given day |
|  | Physical activity self-monitoring | Total number of days that participants registered any steps on their Fitbit account for a given day |
|  | Weight self-monitoring | The number of days they weighed themselves per week |
| Carter (2013)  USA | Dietary self-monitoring | Mean days of dietary recording (complete day is considered as a day with ≥500 and ≤5000 kcal energy recorded) |
